# A New Mechanism for β‐Lactamases: Class D Enzymes Degrade 1β‐Methyl Carbapenems through Lactone Formation

**DOI:** 10.1002/anie.201711308

**Published:** 2018-01-05

**Authors:** Christopher T. Lohans, Emma van Groesen, Kiran Kumar, Catherine L. Tooke, James Spencer, Robert S. Paton, Jürgen Brem, Christopher J. Schofield

**Affiliations:** ^1^ Department of Chemistry University of Oxford Oxford OX1 3TA UK; ^2^ School of Cellular and Molecular Medicine University of Bristol Bristol BS8 1TD UK

**Keywords:** antibiotics, β-lactamases, carbapenems, hydrolases, lactones

## Abstract

β‐Lactamases threaten the clinical use of carbapenems, which are considered antibiotics of last resort. The classical mechanism of serine carbapenemase catalysis proceeds through hydrolysis of an acyl‐enzyme intermediate. We show that class D β‐lactamases also degrade clinically used 1β‐methyl‐substituted carbapenems through the unprecedented formation of a carbapenem‐derived β‐lactone. β‐Lactone formation results from nucleophilic attack of the carbapenem hydroxyethyl side chain on the ester carbonyl of the acyl‐enzyme intermediate. The carbapenem‐derived lactone products inhibit both serine β‐lactamases (particularly class D) and metallo‐β‐lactamases. These results define a new mechanism for the class D carbapenemases, in which a hydrolytic water molecule is not required.

The hydrolysis of carbapenem antibiotics by β‐lactamases (i.e., carbapenemases) represents a major threat to clinically important antibiotics of last resort.^1^, ^2^ The classical mechanism of the serine carbapenemases proceeds through a two‐step pathway involving the formation of an acyl‐enzyme intermediate, which is then hydrolyzed by a nucleophilic water molecule.^3^ All clinically used carbapenem antibiotics have a 6‐hydroxyethyl side chain, the presence of which is proposed to disrupt the hydrolysis step.^4^, ^5^, ^6^ We show that the class D β‐lactamases employ a previously unidentified mechanism for carbapenem degradation in which a β‐lactone is formed (Figure 1 A). We provide evidence that lactone formation occurs through a mechanism in which the hydroxy group of the hydroxyethyl side chain acts as a nucleophile in place of the water molecule required for hydrolysis.

**Figure 1:**
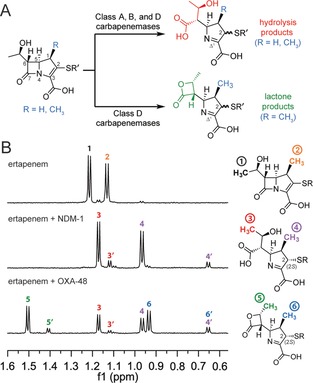
A) Major carbapenem‐derived products formed by β‐lactamases. The C‐2 stereochemistry of the products may depend on the particular enzyme. B) NMR spectra (600 MHz) showing the different product profiles of ertapenem (1 mm) with NDM‐1 (5 μm) or OXA‐48 (5 μm). Note that the minor peaks represent products with an assigned *R* configuration at C‐2 (indicated with a prime symbol; see main text and Figures S2–S5).

While characterizing the carbapenemase activity of the class D serine β‐lactamase (SBL) OXA‐48 (arguably one of the most clinically important carbapenemases)^7^ with the carbapenem ertapenem (Figure S1 in the Supporting Information), we observed two major (and two minor) products by NMR spectroscopy (Figure 1 B). The proton spectrum of one major product was identical to that observed from the hydrolysis of ertapenem by the metallo‐β‐lactamase (MBL) New Delhi MBL‐1 (NDM‐1; Figure 1 B). Further NMR analyses indicated that this corresponds to the anticipated hydrolysis product with the pyrroline ring present as the Δ^1^ tautomer (Table S1–S3 in the Supporting Information); we propose that the configuration at C‐2 is *S* based on NOESY spectra and coupling constant analysis (Figures S2, S3).

Chemical shift assignments for the other major product differed substantially in the region corresponding to the hydroxyethyl side chain (Tables S4, S5). Further analyses led to the proposal that this product contains a β‐lactone, in which the oxygen derived from the hydroxyethyl side chain is covalently bonded to the carbonyl originating from the carbapenem β‐lactam ring (Figure 1 A). The chemical shift assignments for this product resemble those of known β‐lactones (Table S6). Like the major hydrolysis product, the major lactone product is observed as the Δ^1^ tautomer (Tables S4, S5), and is proposed to have an *S* configuration at C‐2 (Figures S4, S5). Notably, β‐lactone formation was observed when ertapenem was incubated with *Escherichia coli* expressing OXA‐48 (Figure S6).

The ertapenem‐derived lactone and hydrolysis products were isolated by HPLC (Figure S7) and characterized by NMR (Figures S8–S15) and MS (Figure S16). The infrared spectrum of the lactone product showed a new peak at 1809 cm^−1^ (Figure S17), which is consistent with a β‐lactone carbonyl.^8^, ^9^ The lactone product also behaved as expected with respect to reaction with nucleophiles (Figure S18, Table S7).

In addition to these major products, lower levels of other species were observed in the reaction mixture (Figure 1 B). Further NMR analyses led to their assignment as stereoisomers of the major lactone and hydrolysis products (Tables S3, S5). The minor products were assigned as having an *R* configuration at C‐2, but are otherwise the same as the 2*S*‐configured lactone and hydrolysis products (Figures S2–S5).

A time‐course analysis monitoring the relative levels of the ertapenem OXA‐48 products suggested that both lactone and hydrolysis products are formed enzymatically (Figure S19). While the assigned 2*S*‐configured lactone product predominates at later time points, the assigned 2*R*‐configured lactone is observed at relatively higher levels at early time points. Incubation of the purified lactone and hydrolysis products in buffered D_2_O showed non‐enzymatic interconversion between the 2*S* and 2*R* diastereomers (Figure S20). Therefore, the greater amount of the 2*S* diastereomers observed in our initial spectra (Figure 1 B) may reflect their relative thermodynamic stabilities, whereas the 2*R* diastereomers may represent a greater proportion of the nascent enzymatic products.

The generality of β‐lactone formation by OXA‐48 was next examined. In addition to ertapenem, β‐lactone formation was observed for meropenem, biapenem, and doripenem (Figures S21‐S24, Tables S8–S16). However, no β‐lactone formation was observed within detection limits for imipenem and panipenem (Figures S25, S26, Tables S17–S20). The product profiles of OXA‐10 and OXA‐23 with these carbapenems are similar (Figures S21–S26). Therefore, within the limits of detection, β‐lactone formation appears to require the presence of a 1β‐methyl substituent. While the MBL carbapenemases NDM‐1, CphA, and L1 demonstrated carbapenemase activity, no β‐lactone formation was observed (Figures S21–S26). Similarly, only hydrolysis products were observed with the class A carbapenemase SFC‐1 (Figures S21–S26).^10^

β‐Lactone formation likely results from intramolecular 4‐*exo*‐trig cyclization^11^ of the hydroxyethyl oxygen onto the ester carbonyl of the acyl‐enzyme intermediate (Figure 2 A). Crystallographic studies of OXA‐1 with doripenem^12^ and of OXA‐58 with a 6α‐hydroxymethyl penicillin^13^ indicate that the catalytically important carbamylated lysine residue interacts with the hydroxyethyl side chain (Figure S27). Furthermore, the hydroxyethyl hydroxy group can adopt an orientation suitable for nucleophilic attack onto the ester carbonyl (i.e., the Bürgi–Dunitz trajectory),^14^ with the carbamylated lysine apparently positioned to act as a general base.^12^, ^13^ Although this conformation of the hydroxyethyl side chain was not observed in related crystal structures (Figure S27), these structures depict enzymes in which the lysine is not carbamylated (e.g., due to low pH or mutations).^5^, ^15^, ^16^

**Figure 2:**
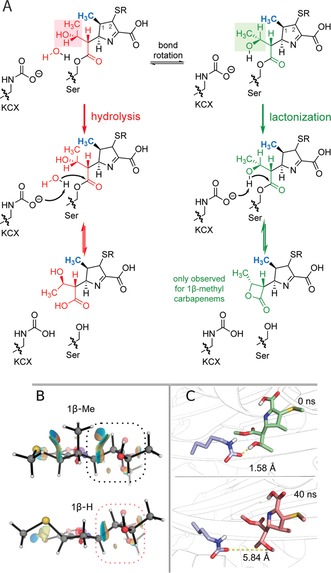
A) Proposed outline mechanisms for carbapenem hydrolysis and lactone formation. KCX labels the carbamylated lysine residue, which acts as a general base. Lactone formation was only observed for carbapenems bearing a 1β‐methyl group (blue). The timing of the imine tautomerization is not defined, but likely occurs (in part) prior to fragmentation of the acyl‐enzyme complex. It is unclear based on the products observed whether a particular configuration at C‐2 predominates. B) DFT non‐covalent interaction isosurfaces showing unfavorable steric interactions between the carbapenem 1β‐Me and hydroxyethyl groups, which are alleviated in the corresponding 1β‐H system. Reduced density gradient isosurface *s*=0.5, *ρ*(**r**) signλ_2_ (e/au) color scale runs from −0.02 (blue) to 0.02 (red). C) Representations of two major conformations of the hydroxyethyl group observed during MD simulation of the OXA‐1 doripenem complex;^12^ the upper conformation appears to be more favorable for β‐lactone formation. Note that the doripenem thioether side chain and serine backbone are represented as methyl groups.

Kinetic studies of OXA enzymes show that the *k*_cat_ values for 1β‐methyl‐substituted carbapenems tend to be 10–100‐fold less than for those with a 1β‐proton.^17^, ^18^ Non‐covalent interaction plots indicate that the 1β‐methyl group may interact sterically with the hydroxyethyl group in the acyl‐enzyme complex, which is largely alleviated in the corresponding 1β‐proton system (Figure 2 B). Molecular dynamics simulations (100 ns) of the acyl‐enzyme complex derived from OXA‐1 and doripenem^12^ also suggest that the conformation of the hydroxyethyl group is influenced by the 1β‐substituent, with the system bearing a 1β‐proton showing more flexibility (Tables S21–S23, Figures S28–S31). Notably, in the MD simulations, the 1β‐methyl system initially alternates between two conformations (Figure 2 C), one of which appears to be consistent with that expected for β‐lactone formation (Figure S27).

We propose that the 1β‐methyl group destabilizes the conformation(s) of the hydroxyethyl side chain required for hydrolysis of the acyl‐enzyme complex. Consequently, the acyl‐enzyme complexes of 1β‐methyl carbapenems with OXA enzymes are more resistant to hydrolysis than are those derived from carbapenems without a 1β‐methyl group. The class D β‐lactamases have apparently, at least in part, overcome this inhibition by catalyzing lactone formation. However, lactone formation may only represent a competitive pathway if hydrolysis is disfavoured (i.e., by the 1β‐methyl group), thus explaining why only hydrolysis is observed for carbapenems without a 1β‐methyl group.

Since the lactone products are isomeric with β‐lactams, it was considered that they may interact with β‐lactamases and penicillin‐binding proteins (PBPs). Indeed, the ertapenem‐derived lactone was hydrolyzed by SBLs with carbapenemase activity (SFC‐1, OXA‐23, and OXA‐48; Figure 3 A); by contrast, no lactone hydrolysis was observed with carbapenemase MBLs (NDM‐1, CphA, and L1; Figure 3 A). Acylation of some SBLs, such as OXA‐10, by the ertapenem‐derived lactone was observed by MS (Figure 3 B, Figure S34). However, the lactone did not show antibiotic activity at the levels tested against *E. coli* (Figure S35), nor was acylation observed for the non‐essential PBP‐5 from *E. coli* (Figure S34).

**Figure 3:**
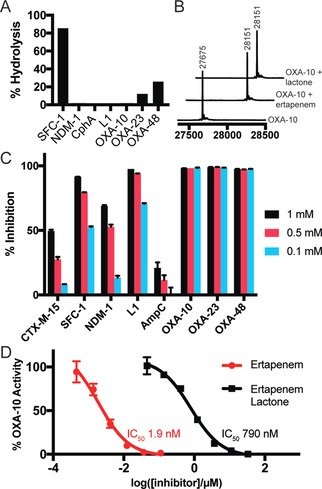
A) Extent of hydrolysis of the ertapenem lactone by a panel of carbapenemases (1 h, 0.5 mm lactone, 5 μm enzyme). B) Mass spectra showing acylation of OXA‐10 (1 μm) by ertapenem (100 μm) and ertapenem lactone (100 μm) after 30 min. C) Inhibitory activity of ertapenem lactone (1 mm, 0.5 mm, 0.1 mm) against a panel of SBLs and MBLs. D) Comparison of the inhibitory activity of ertapenem and ertapenem lactone against OXA‐10.

The ertapenem‐derived lactone inhibited both SBLs and MBLs when tested at high concentrations (Figure 3 C). Class D β‐lactamases were inhibited particularly strongly, with IC_50_ values of 790 nm, 1.2 μm, and 940 nm obtained for OXA‐10, OXA‐23, and OXA‐48, respectively (Figure 3 D, Figure S36). However, the potency of the lactone was relatively low compared to ertapenem (IC_50_ values of 1.9 nm, 65 nm, and 10 nm for OXA‐10, OXA‐23, and OXA‐48, respectively; Figure 3 D, Figures S36, S37). The observed inhibition of some MBLs is interesting, given the lack of clinically used inhibitors for these enzymes.^19^

The degradation of carbapenems through β‐lactone formation represents a new mechanism for the carbapenemases. Carbapenems are potent inhibitors of many PBPs and SBLs, with which they rapidly react to form an acyl‐enzyme complex; this complex is stabilized due to disruption of the “hydrolytic water” by the hydroxyethyl side chain. Thus, the results presented here suggest that, in the case of clinically important 1β‐methyl carbapenems, the class D β‐lactamases may have evolved to overcome this disruption, in part by promoting lactone formation. Although the lactones represent a type of product inhibition, their potency is less than that of the parent carbapenems. It will be of interest to see whether other mechanisms for fragmentation of the acyl‐enzyme ester evolve.

Avibactam, which was recently introduced as the first clinically used non‐β‐lactam‐containing β‐lactamase inhibitor, works through a reversible acylation mechanism.^20^ The observation that carbapenem‐derived lactones acylate serine β‐lactamases suggests that new classes of β‐lactamase inhibitors based on lactones are possible. In this regard, it is notable that the β‐lactone orlistat is an important medicine,^21^ and that lactone antibiotics (e.g., obafluorin)^8^ and inhibitors of serine proteases (and lactam derivatives thereof)^22^, ^23^ have been reported. Carbapenem‐derived β‐lactones thus represent a novel scaffold for β‐lactamase inhibition that, with optimization, may prove comparable in potency to the carbapenems.

## Conflict of interest

The authors declare no conflict of interest.

## Supporting information

As a service to our authors and readers, this journal provides supporting information supplied by the authors. Such materials are peer reviewed and may be re‐organized for online delivery, but are not copy‐edited or typeset. Technical support issues arising from supporting information (other than missing files) should be addressed to the authors.

Supplementary

## References

[1] A. M. Queenan , K. Bush , Clin. Microbiol. Rev. 2007, 20, 440. 17630334 10.1128/CMR.00001-07 PMC1932750

[2] G. Patel , R. A. Bonomo , Front. Microbiol. 2013, 4, 48. 23504089 10.3389/fmicb.2013.00048 PMC3596785

[3] J. H. Jeon , J. H. Lee , J. J. Lee , K. S. Park , A. M. Karim , C. R. Lee , B. C. Jeong , S. H. Lee , Int. J. Mol. Sci. 2015, 16, 9654. 25938965 10.3390/ijms16059654 PMC4463611

[4] L. Maveyraud , L. Mourey , L. P. Kotra , J.-D. Pedelacq , V. Guillet , S. Mobashery , J.-P. Samama , J. Am. Chem. Soc. 1998, 120, 9748.

[5] C. A. Smith , N. T. Antunes , N. K. Stewart , M. Toth , M. Kumarasiri , M. Chang , S. Mobashery , S. B. Vakulenko , Chem. Biol. 2013, 20, 1107. 24012371 10.1016/j.chembiol.2013.07.015 PMC3888872

[6] M. Toth , C. A. Smith , N. T. Antunes , N. K. Stewart , L. Maltz , S. B. Vakulenko , Acta Crystallogr. Sect. D 2017, 73, 692. 10.1107/S2059798317008671 PMC5571744 28777084

[7] L. Poirel , A. Potron , P. Nordmann , J. Antimicrob. Chemother. 2012, 67, 1597. 22499996 10.1093/jac/dks121

[8] A. A. Tymiak , C. A. Culver , M. F. Malley , J. Z. Gougoutas , J. Org. Chem. 1985, 50, 5491.

[9] Y. Pu , F. M. Martin , J. C. Vederas , J. Org. Chem. 1991, 56, 1280.

[10] F. Fonseca , E. I. Chudyk , M. W. van der Kamp , A. Correia , A. J. Mulholland , J. Spencer , J. Am. Chem. Soc. 2012, 134, 18275. 23030300 10.1021/ja304460j

[11] J. Baldwin , CIBA Found. Symp. 1978, 85. 10.1002/9780470720349.ch7 246784

[12] K. D. Schneider , M. E. Karpen , R. A. Bonomo , D. A. Leonard , R. A. Powers , Biochemistry 2009, 48, 11840. 19919101 10.1021/bi901690r PMC2805451

[13] S. Pratap , M. Katiki , P. Gill , P. Kumar , D. Golemi-Kotra , Antimicrob. Agents Chemother. 2016, 60, 75. 10.1128/AAC.01393-15 PMC4704147 26459904

[14] H. B. Burgi , J. D. Dunitz , E. Shefter , J. Am. Chem. Soc. 1973, 95, 5065.

[15] K. D. Schneider , C. J. Ortega , N. A. Renck , R. A. Bonomo , R. A. Powers , D. A. Leonard , J. Mol. Biol. 2011, 406, 583. 21215758 10.1016/j.jmb.2010.12.042 PMC3057435

[16] C. M. June , T. J. Muckenthaler , E. C. Schroder , Z. L. Klamer , Z. Wawrzak , R. A. Powers , A. Szarecka , D. A. Leonard , Protein Sci. 2016, 25, 2152. 27636561 10.1002/pro.3040 PMC5119573

[17] A. M. Queenan , W. Shang , R. Flamm , K. Bush , Antimicrob. Agents Chemother. 2010, 54, 565. 19884379 10.1128/AAC.01004-09 PMC2798497

[18] S. Oueslati , P. Nordmann , L. Poirel , J. Antimicrob. Chemother. 2015, 70, 1059. 25583748 10.1093/jac/dku524

[19] C. Bebrone , Biochem. Pharmacol. 2007, 74, 1686. 17597585 10.1016/j.bcp.2007.05.021

[20] D. E. Ehmann , H. Jahić , P. L. Ross , R. F. Gu , J. Hu , G. Kern , G. K. Walkup , S. L. Fisher , Proc. Natl. Acad. Sci. USA 2012, 109, 11663. 22753474 10.1073/pnas.1205073109 PMC3406822

[21] R. Guerciolini , Int. J. Obes. Relat. Metab. Disord. 1997, 21, S12. 9225172

[22] N. O. Sykes , S. J. Macdonald , M. I. Page , J. Med. Chem. 2002, 45, 2850. 12061887 10.1021/jm0111245

[23] M. E. Migaud , R. C. Wilmouth , G. I. Mills , G. J. Wayne , C. Risley , C. Chambers , S. J. Macdonald , C. J. Schofield , Chem. Commun. 2002, 1274. 10.1039/b111627d 12109112

