# Supplementary material for: A New Mechanism for β‐Lactamases: Class D Enzymes Degrade 1β‐Methyl Carbapenems through Lactone Formation
Source: Angew Chem Int Ed Engl. 2018 Jan 5;57(5):1282–5. doi: 10.1002/anie.201711308 (PMC5817396; doi:10.1002/anie.201711308)
Supplement: Supplementary file 1 — Supplementary [file ANIE-57-1282-s001.pdf]

Angewandte Chemie
Eine Zeitschrift der Gesellschaft Deutscher Chemiker

Supporting Information

A New Mechanism for β-Lactamases: Class D Enzymes Degrade 1β-Methyl Carbapenems through Lactone Formation

*Christopher T. Lohans, Emma van Groesen, Kiran Kumar, Catherine L. Tooke, James Spencer, Robert S. Paton, Jürgen Brem, and Christopher J. Schofield**

anie_201711308_sm_miscellaneous_information.pdf

Supporting Information Contents

Experimental	S3
Fig. S1. Structures of the carbapenems used in this study	S7
Chemical shift assignments	
Ertapenem	
Table S1. Carbapenem	S8
Table S2. (2*S*)-Hydrolysis Product	S9
Table S3. (2*R*)-Hydrolysis Product	S10
Table S4. (2*S*)-Lactone Product	S11
Table S5. (2*R*)-Lactone Product	S12
Fig. S2. Stereochemical analysis of ertapenem hydrolysis products by coupling constants	S13
Fig. S3. NOESY stereochemical analysis of ertapenem hydrolysis products	S14
Table S6. Literature ^{13}C-chemical shift assignments for β-lactones	S15
Fig. S4. Stereochemical analysis of ertapenem lactone products by coupling constants	S16
Fig. S5. NOESY stereochemical analysis of ertapenem lactone products	S17
Fig. S6. Formation of the ertapenem-derived lactones by *E. coli* expressing OXA-48	S18
Fig. S7. HPLC purification of OXA-48 ertapenem products	S19
Fig. S8-S11. NMR spectra of ertapenem lactone products	S20
Fig. S12-S15. NMR spectra of ertapenem hydrolysis products	S24
Fig. S16. Mass spectra of ertapenem OXA-48 products	S28
Fig. S17. Infrared spectra of ertapenem and ertapenem products	S29
Fig. S18. Reaction of the ertapenem-derived lactone with L-cysteine	S30
Table S7. Chemical shift assignments for the L-cysteine adduct of ertapenem	S31
Fig. S19. OXA-48 ertapenem NMR time course	S32
Fig. S20. Exchange of ertapenem product C-2 protons with solvent	S33
Fig. S21-S26. NMR spectra of carbapenem/carbapenemase reaction mixtures	S35
Chemical shift assignments	
Biapenem	
Table S8. Carbapenem	S41
Table S9. Major Lactone Product	S42
Table S10. Major Hydrolysis Product	S43
Doripenem	
Table S11. Carbapenem	S44
Table S12. Major Lactone Product	S45
Table S13. Major Hydrolysis Product	S46
Meropenem	
Table S14. Carbapenem	S47
Table S15. Major Lactone Product	S48
Table S16. Major Hydrolysis Product	S49
Imipenem	
Table S17. Carbapenem	S50
Table S18. Major Hydrolysis Product	S51
Panipenem	
Table S19. Carbapenem	S52
Table S20. Major Hydrolysis Product	S53

Figure S27. Previously reported crystal structures of OXA enzymes and carbapenems S54
Table S21-S23. Cartesian coordinates used in MD simulations S56
Fig. S28-S29. MDS time plot for the doripenem-derived OXA-1 acyl-enzyme complexes S59
Fig. S30-S31. MDS dihedral angle plots for the doripenem-derived OXA-1 acyl-enzyme complexes S61
Fig. S32-S33. MDS root-mean-square deviation plots S63
Fig. S34. Extent of acylation of non-carbapenemase SBLs and PBPs by ertapenem lactone S65
Fig. S35. Antibiotic testing of the ertapenem-derived lactone S66
Fig. S36. OXA enzyme inhibition by ertapenem and the ertapenem-derived lactone S67
Fig. S37. Impact of ertapenem on the activity of SBLs and MBLs S68
Fig. S38. Interactions of carbapenems and carbapenem-derived lactones with β-lactamases S69
Fig. S39-S82. NMR spectra of carbapenems and carbapenem-derived products S70
References S92

Experimental

General

Carbapenems were from Glentham Life Sciences (biapenem, doripenem, ertapenem), Molekula (meropenem, imipenem), and Ontario Chemicals Inc. (panipenem). Infrared spectra were recorded on a Bruker Tensor 27 FT-IR spectrometer. Mass spectra were acquired using a Waters Micromass LCT Premier XE spectrometer fitted with an Acquity UPLC system. All enzymes were expressed and purified as previously described.[1,2]

NMR Spectroscopy

NMR spectra were acquired using a Bruker Avance III 700 MHz spectrometer equipped with a TCI inverse cryoprobe, and a Bruker AVIII HD 600 MHz spectrometer equipped with a BB-F/^{1}H Prodify N_2 cryoprobe. Unless otherwise stated, samples were prepared in 50 mM sodium phosphate, pH 7.5, 10 % D_2O. Water suppression was accomplished by pre-saturation or by excitation sculpting with perfect echo. All spectra were acquired at 298 K.

Chemical shift assignments for intact carbapenems were made using 5 mM samples, on the basis of ^{1}H, COSY, HSQC, and HMBC spectra. Chemical shift assignments for the hydrolysis and lactone products were made using a sample consisting of 2 mM carbapenem with 5 μM OXA-48 (for biapenem, doripenem, meropenem, imipenem, and panipenem) or using 5 mM ertapenem hydrolysis and lactone products (HPLC-purified), based on ^{1}H, COSY, HSQC, and HMBC spectra. The concentrations of enzymes and carbapenems used to test for product formation are as stated in the relevant figures. The enzymatic hydrolysis of the ertapenem-derived lactone was tested following a 1 h incubation of 0.5 mM lactone with 5 μM enzyme in 50 mM sodium phosphate, pH 7.5, and measured by ^{1}H-NMR. Stereochemical analysis of the ertapenem

hydrolysis and lactone products was carried out using a 1D SPFGSE 1H,1H-NOESY pulse sequence employing water pre-saturation and a 300 ms mixing time.

Purification of Ertapanem Lactone and Hydrolysis Products

A mixture of 10 mM ertapenem and 25 μM OXA-48 in 50 mM sodium phosphate, pH 7.5, was incubated at room temperature for 4 h. The enzyme was removed by passing the reaction mixture through an Amicon Ultra-0.5 mL centrifugal filter (3 kDa molecular weight cut-off). The filtered enzymatic products were purified using a JASCO high-pressure liquid chromatography (HPLC) platform equipped with PU-2086 Plus preparative scale pumps. A SunFire™ Prep C18 column (10 x 150 mm, 5 μm particle size; Waters) was used. Mobile phases (A) water with 0.1 % trifluoroacetic acid (TFA), and (B) acetonitrile with 0.1 % TFA were used at an overall flow-rate of 3 mL/min, while elution was monitored at 254 nm. The enzymatic products were separated using a gradient running from 10 % B to 50 % B over 15 min. Collected fractions were frozen on liquid nitrogen and lyophilized. The identities of the eluted peaks were determined by mass spectrometry and NMR spectroscopy.

Inhibition Studies

The impact of ertapenem (1 mM, 0.5 mM, 0.1 mM) and ertapenem lactone (1 mM, 0.5 mM, 0.1 mM) on the hydrolysis of the fluorogenic substrate FC-5[3] (5 μM) was tested with CTX-M-15 (50 pM), SFC-1 (500 pM), New Delhi metallo-β-lactamase-1 (NDM-1; 10 pM), L1 (50 pM), AmpC (500 pM), OXA-10 (1 nM), OXA-23 (12.5 nM), and OXA-48 (12.5 nM).[1,4] Reactions were carried out in 100 mM sodium phosphate, pH 7.5, 0.01 % Triton X-100 (for CTX-M-15, SFC-1, AmpC, OXA-10, OXA-23, and OXA-48), or 50 mM HEPES, pH 7.5, 10 μM $ZnSO_4$, 10 μg/mL BSA, 0.01 % Triton X-100 (for NDM-1 and L1). The enzyme was pre-incubated with ertapenem, or ertapenem lactone, for 10 min prior to the addition of FC-5. Assays were performed

in triplicate using 384-well black µclear® plates (Greiner Bio-One) at 25 °C and measured with a PHERAstar FS microplate reader (BMG Labtech). IC_{50} values were determined using eight different concentrations of ertapenem and ertapenem lactone, and nonlinear regression analysis was performed using Prism 7 (GraphPad).

Computational Methodology

MD simulations were carried out for 100 ns each. Starting structures were taken from PDB 3ISG[5] and used as a template for building the OXA-1:doripenem systems. AMBER12[6] was used with the Amberff12SB force field to define protein partial charges. Hydrogen atom addition was performed with LEaP. Systems were solvated in a 10 Å truncated octahedral box of TIP3P water[7] and were neutralized explicitly with either sodium or chloride counterions. Atomic partial charges for the 1β-Me and 1β-H doripenem-derived ligands and for the carbamylated lysine correspond to the Restrained Electrostatic Potential (RESP)[8] charges shown in Table S21-S23. The final systems were minimized for 1,000 cycles of steepest-descent minimization followed by 1,000 cycles of conjugate-gradient minimization to remove close van der Waals contacts using the *sander* program in AMBER12. Equilibration was achieved using PMEMD to heat the systems to 310 K followed by independent MD simulations performed with a periodic boundary condition at a constant pressure of 1 atm with isotropic molecule-based scaling at a time step of 2.0 fs. All simulations used a dielectric constant of 1.0, Particle Mesh Ewald summation[9] to calculate long-range electrostatic interactions, and bond-length constraints were applied to all bonds to H atoms. Trajectories were saved at 20 ps intervals and visualized using VMD.[10]

Density functional theory (DFT) non-covalent interaction (NCI) plots[11] of the 1β-Me and 1β-H doripenem-derived ligands were generated from their wave function and electron density at the ωb97xd/def2tzvp level of theory. Geometries were extracted from a snapshot taken at 40 ns of

the MD simulation for the OXA-1:1β-Me doripenem system, manually modified to the 1β-H counterpart, and optimized. These plots allowed the qualitative visualization of attractive or repulsive non-covalent interactions mapped onto a reduced density gradient (RDG) isosurface, where red and blue represent repulsive non-bonding and strong attractive interactions, respectively.

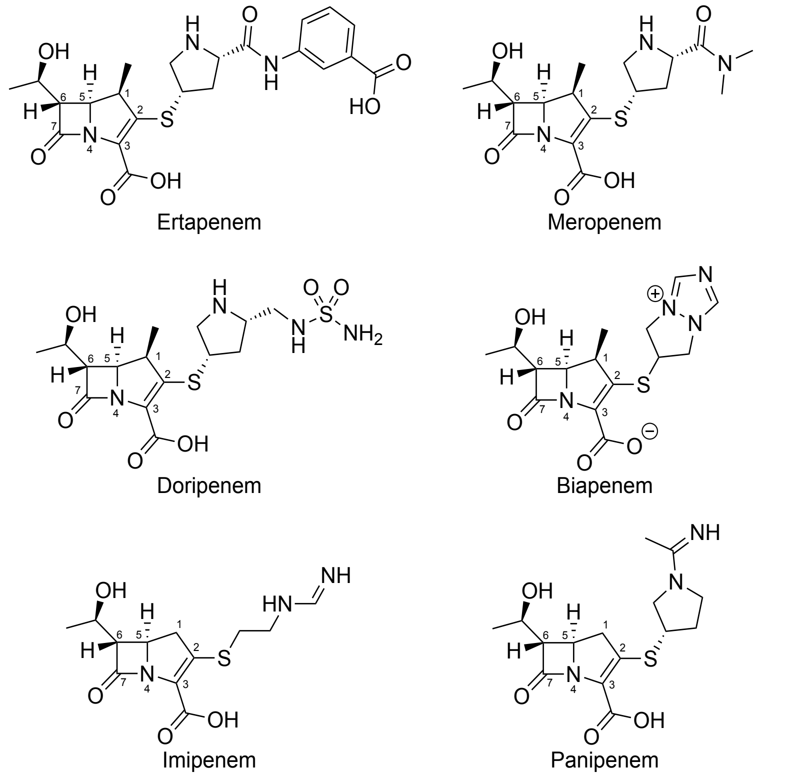

Figure S1. Structures of the carbapenems used in this study. These carbapenems were chosen due to the presence (ertapenem, meropenem, doripenem, biapenem) or absence (imipenem, panipenem) of a 1β-methyl substituent.

Table S1. Chemical shift assignments for ertapenem.

Position	^{13}C (ppm)[a]	^{1}H (ppm)
1	45.54	3.33
2	143.29	
3	134.51	
4		
5	58.82	4.14
6	61.18	3.37
7	179.15	
8	67.98	4.18
9	22.92	1.23
10		
11	18.70	1.14
12	56.52	3.09, 3.45
13	44.67	3.83
14	38.35	1.93, 2.79
15	63.15	4.35
16	174.84	
17		
18	124.92	7.79
19		
20	128.85	7.64
21	131.9	7.43
22	127.43	7.62
23	177.75	

[a]Spectra are shown in Figures S39-42.

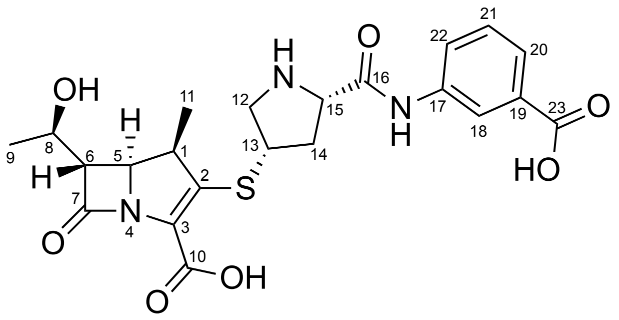

Table S2. Chemical shift assignments for the ertapenem-derived (2*S*)-hydrolysis product.

Position	^{13}C (ppm)[a]	^{1}H (ppm)
1	46.74	2.47
2	59.85	3.83
3	175.43	
4		
5	75.61	4.26
6	58.74	2.54
7	183.00	
8	70.92	3.96
9	23.32	1.19
10		
11	16.63	1.00
12	55.06	3.28, 3.70
13	44.22	3.68
14	39.49	2.02, 2.86
15	62.75	4.41
16	171.69	
17		
18	124.91	7.80
19		
20	128.92	7.65
21	131.99	7.43
22	127.29	7.57
23	177.76	

[a]Spectra are shown in Figures S12-S15.

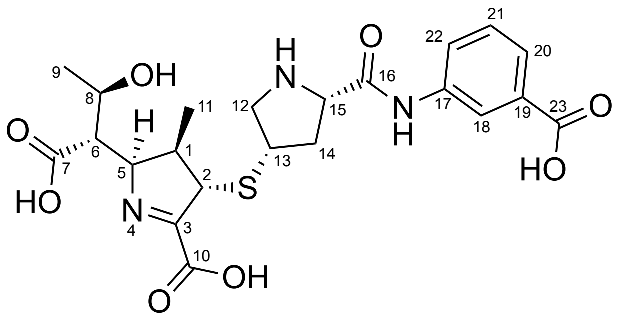

Table S3. Chemical shift assignments for the ertapenem-derived (2*R*)-hydrolysis product.

Position	^{1}H (ppm)[a]
1	2.57
2	4.15
3	
4	
5	3.79
6	2.51
7	
8	3.79
9	1.12
10	
11	0.65
12	3.33, 3.68
13	3.81
14	2.39, 2.87
15	4.52
16	
17	
18	7.82
19	
20	7.63
21	7.43
22	7.57
23	

[a]Spectra are shown in Figures S12-S15.

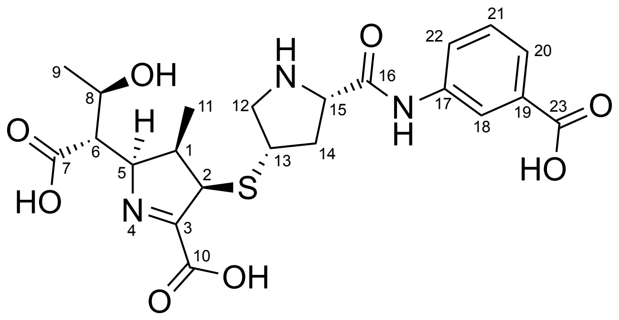

Table S4. Chemical shift assignments for the ertapenem-derived (2*S*)-lactone product.

Position	^{13}C (ppm)[a]	^{1}H (ppm)
1	45.80	2.48
2	59.37	3.89
3	176.32	
4		
5	71.28	4.51
6	55.16	4.03
7	175.64	
8	75.92	4.96
9	17.97	1.52
10		
11	16.68	0.95
12	55.28	3.21, 3.62
13	44.68	3.65
14	39.86	2.01, 2.80
15	62.75	4.33
16	173.57	
17		
18	124.79	7.81
19		
20	128.84	7.65
21	131.98	7.42
22	127.30	7.56
23	177.78	

[a]Spectra are shown in Figures S8-S11.

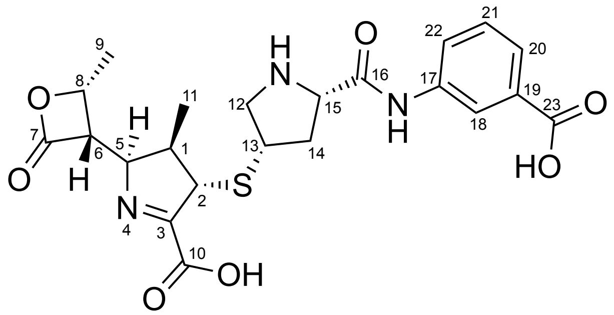

Table S5. Chemical shift assignments for the ertapenem-derived (2*R*)-lactone product.

Position	^{1}H (ppm)[a]
1	2.68
2	4.20
3	
4	
5	4.28
6	3.86
7	
8	4.86
9	1.40
10	
11	0.65
12	3.28, 3.63
13	3.79
14	2.35, 2.84
15	4.45
16	
17	
18	7.81
19	
20	7.65
21	7.42
22	7.56
23	

[a]Spectra are shown in Figures S8-S11.

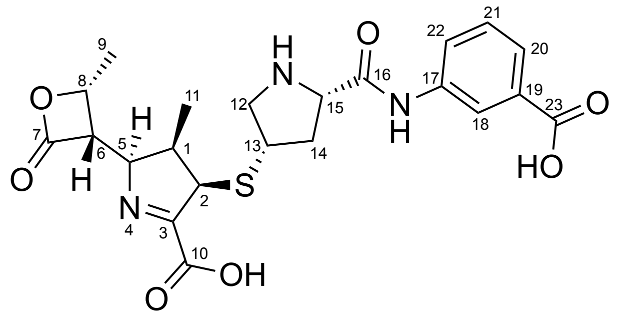

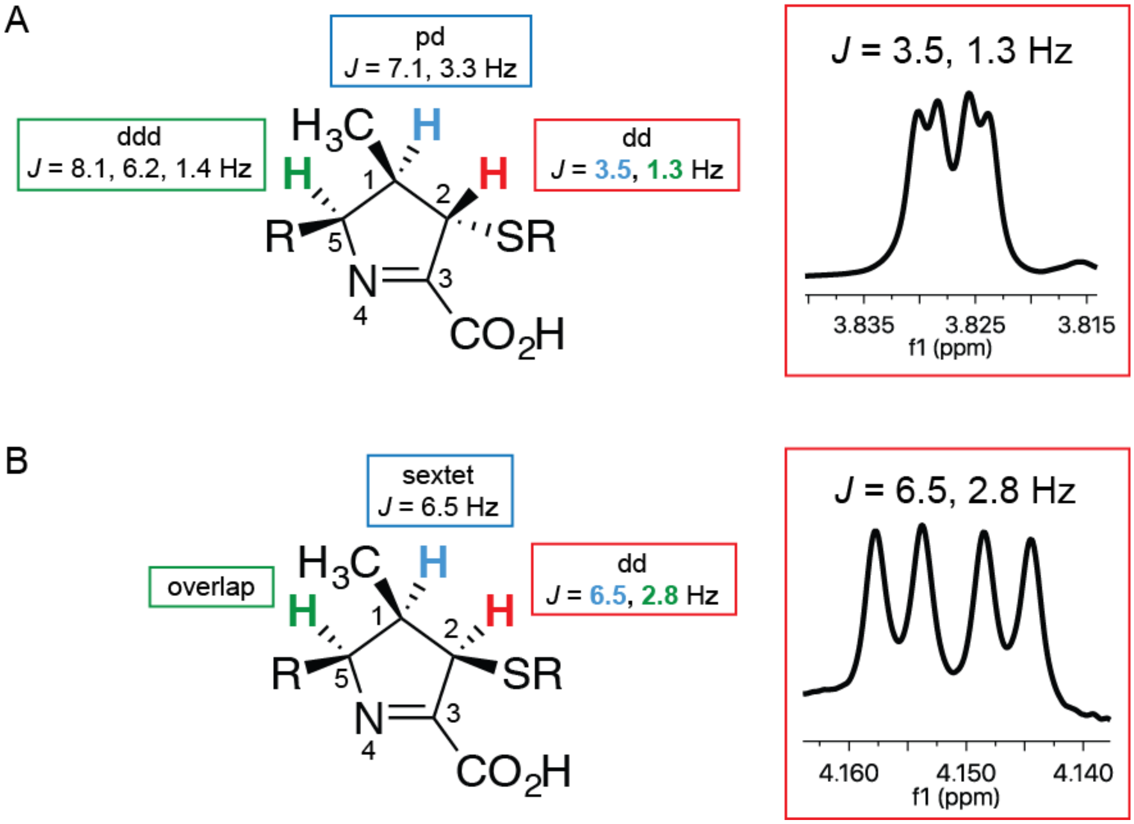

Figure S2. Stereochemical analysis of the observed ertapenem-derived hydrolysis products based on coupling constants. Coupling constants of protons on the pyrroline ring of (A) the (2*S*)-ertapenem hydrolysis product and (B) the (2*R*)-ertapenem hydrolysis product. The proposed stereochemical assignments are supported by the observed coupling constants between the protons at C-1 and C-2. The *trans* orientation of these two protons in the (2*S*)-diastereomer is consistent with the small coupling constant (3.5 Hz), while the *cis* orientation in the (2*R*)-diastereomer is supported by the larger coupling constant (6.5 Hz). While the coupling pattern of the proton at C-5 of the ertapenem-derived (2*R*)-hydrolysis product could not be resolved, long-range coupling was observed between this proton and the proton at C-2 by COSY. The C-2 and C-5 protons of the ertapenem-derived (2*S*)-hydrolysis product also show long-range coupling (*J* 1.3 Hz).

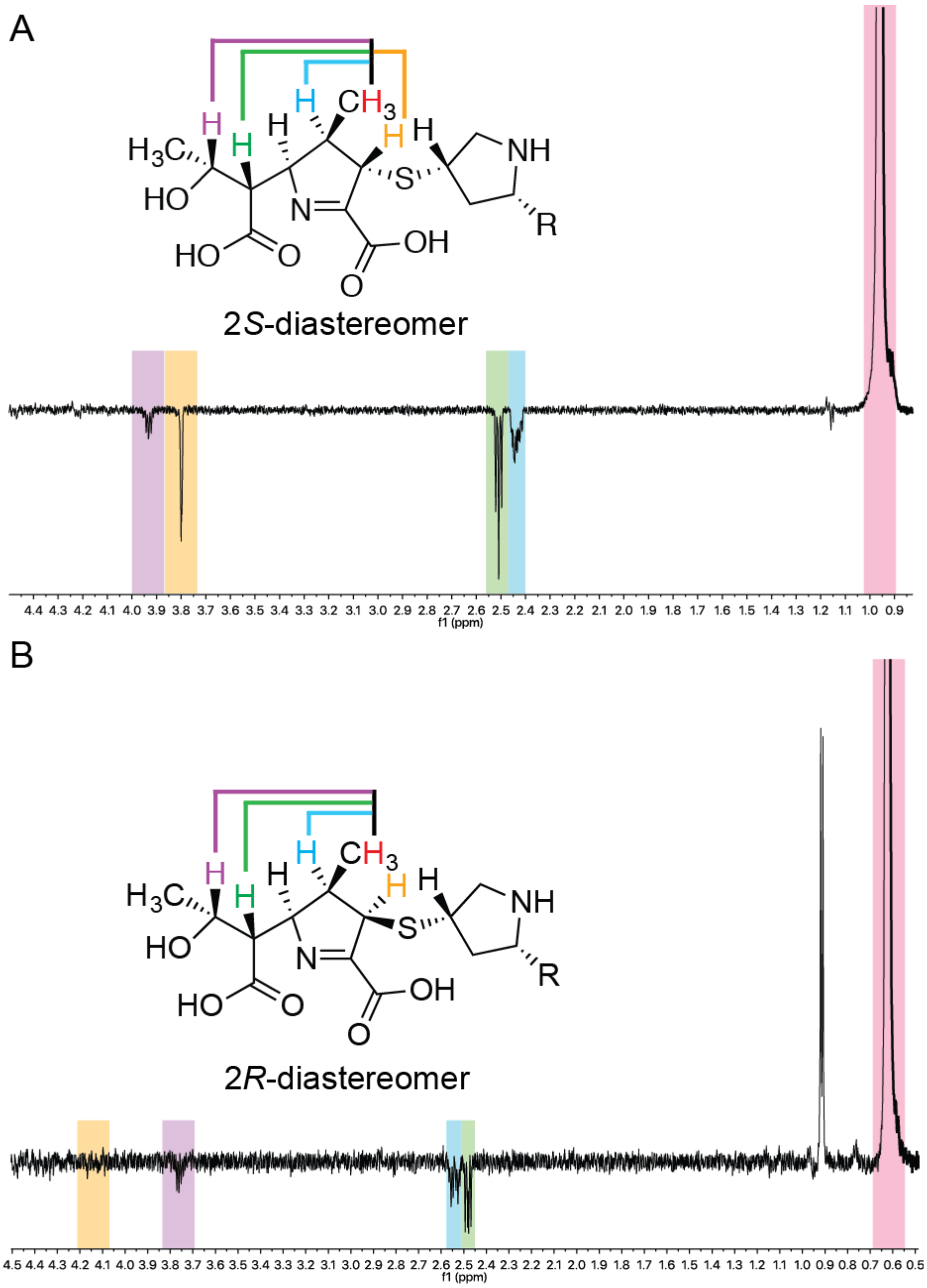

Figure S3. NOESY stereochemical analysis of the observed ertapenem-derived hydrolysis products. 1D-NOESY spectra (700 MHz) with selective irradiation of the 1β-methyl group of the assigned ertapenem-derived (A) (2*S*)- and (B) (2*R*)-hydrolysis products. The results imply that the 1β-methyl group (red) is in close proximity to the C-2 proton (yellow) in the ertapenem-derived (2*S*)-hydrolysis product, as shown in panel A; however, no NOE was observed between these protons in the ertapenem-derived (2*R*)-hydrolysis product, as shown in panel B. Spectra were measured with a 10 mM sample of the HPLC-purified ertapenem hydrolysis product [5:1 mixture of (2*S*)- and (2*R*)-diastereomers] in 50 mM sodium phosphate, pH 7.5, 10 % D_2O.

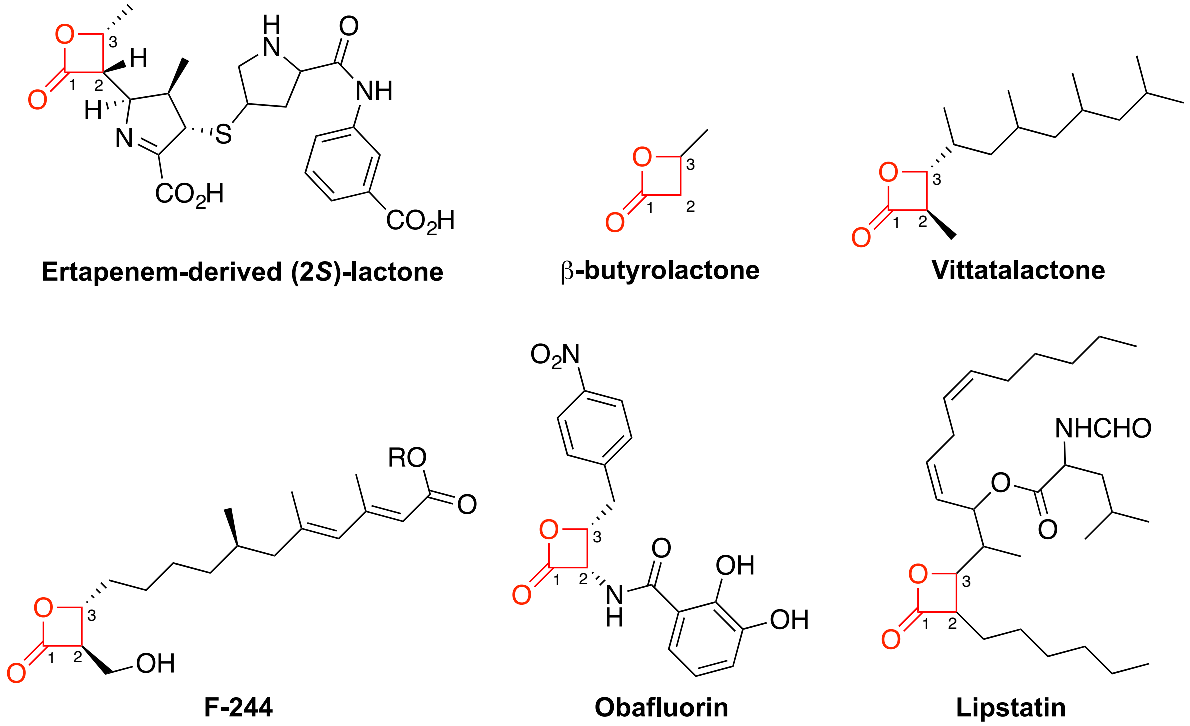

Table S6. Literature ^{13}C-chemical shift assignments for β-lactones.

Position	Ertapenem-derived (2*S*)-lactone[a]	β-Butyrolactone[a]	Vittatalactone[12]
	^{13}C (ppm)	^{13}C (ppm)	^{13}C (ppm)
1	175.64	175.63	172.1
2	55.16	45.91	48.9
3	75.92	73.56	83.8

Position	F-244[13]	Obafluorin[14]	Lipstatin[15]
	^{13}C (ppm)	^{13}C (ppm)	^{13}C (ppm)
1	169.7	169.25	170.83
2	58.6	58.45	57.17
3	74.9	76.81	74.90

[a]Chemical shifts assigned in this work

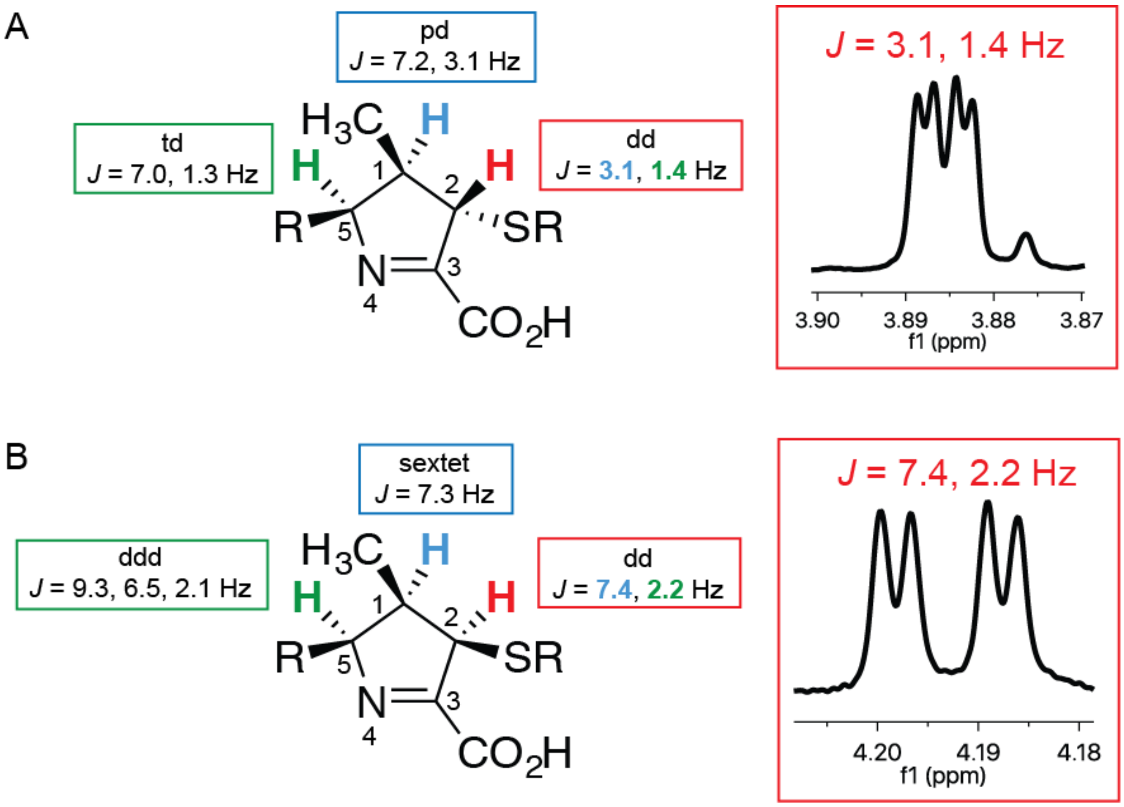

Figure S4. Stereochemical analysis of the observed ertapenem-derived lactone products based on coupling constants. Coupling constants of protons on the pyrroline ring of (A) the (2*S*)-ertapenem lactone product and (B) the (2*R*)-ertapenem lactone product. The proposed stereochemical assignments are supported by the coupling constants between the protons at C-1 and C-2. The *trans* orientation of these two protons in the (2*S*)-diastereomer is supported by the small coupling constant (3.1 Hz), while the *cis* orientation in the (2*R*)-diastereomer is supported by the larger coupling constant (7.4 Hz). Long-range coupling [*J* 1.4 Hz and 2.2 Hz for the (2*S*)- and (2*R*)-diastereomers, respectively] is observed between the protons at C-2 and C-5, as also observed by COSY.

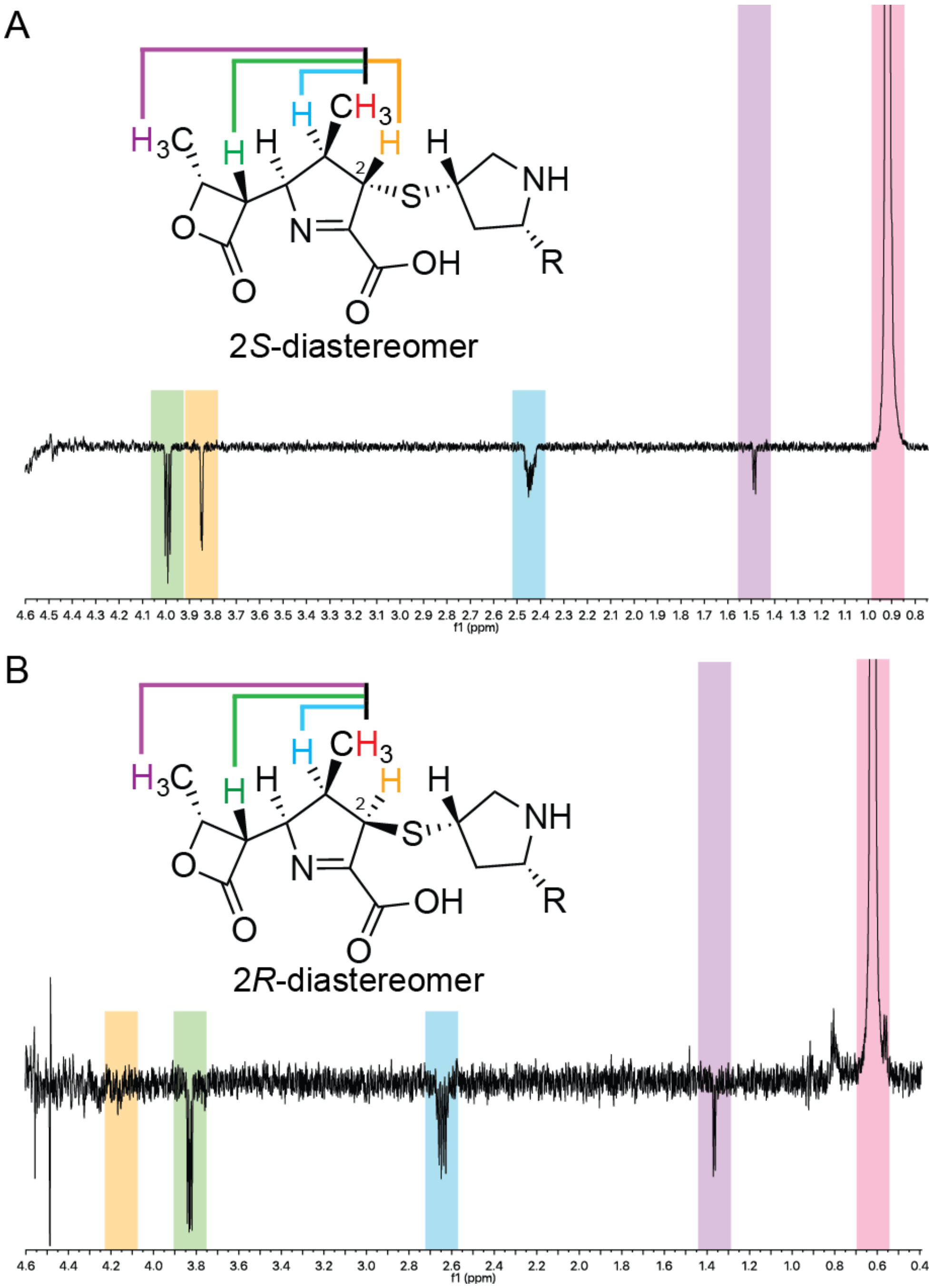

Figure S5. NOESY stereochemical analysis of the observed ertapenem-derived lactone products. 1D-NOESY spectra (700 MHz) with selective irradiation of the 1β-methyl group of the ertapenem-derived (A) (2*S*)- and (B) (2*R*)-lactone products. The results imply that the 1β-methyl group (red) is in close proximity to the C-2 proton (yellow) in the ertapenem-derived (2*S*)-lactone, as shown in panel A; however, no NOE was observed between these protons in the ertapenem-derived (2*R*)-derived lactone, as shown in in panel B. Spectra were measured with a 10 mM sample of purified ertapenem lactone [5:1 mixture of (2*S*)- and (2*R*)-diastereomers] in 50 mM sodium phosphate, pH 7.5, 10 % D_2O.

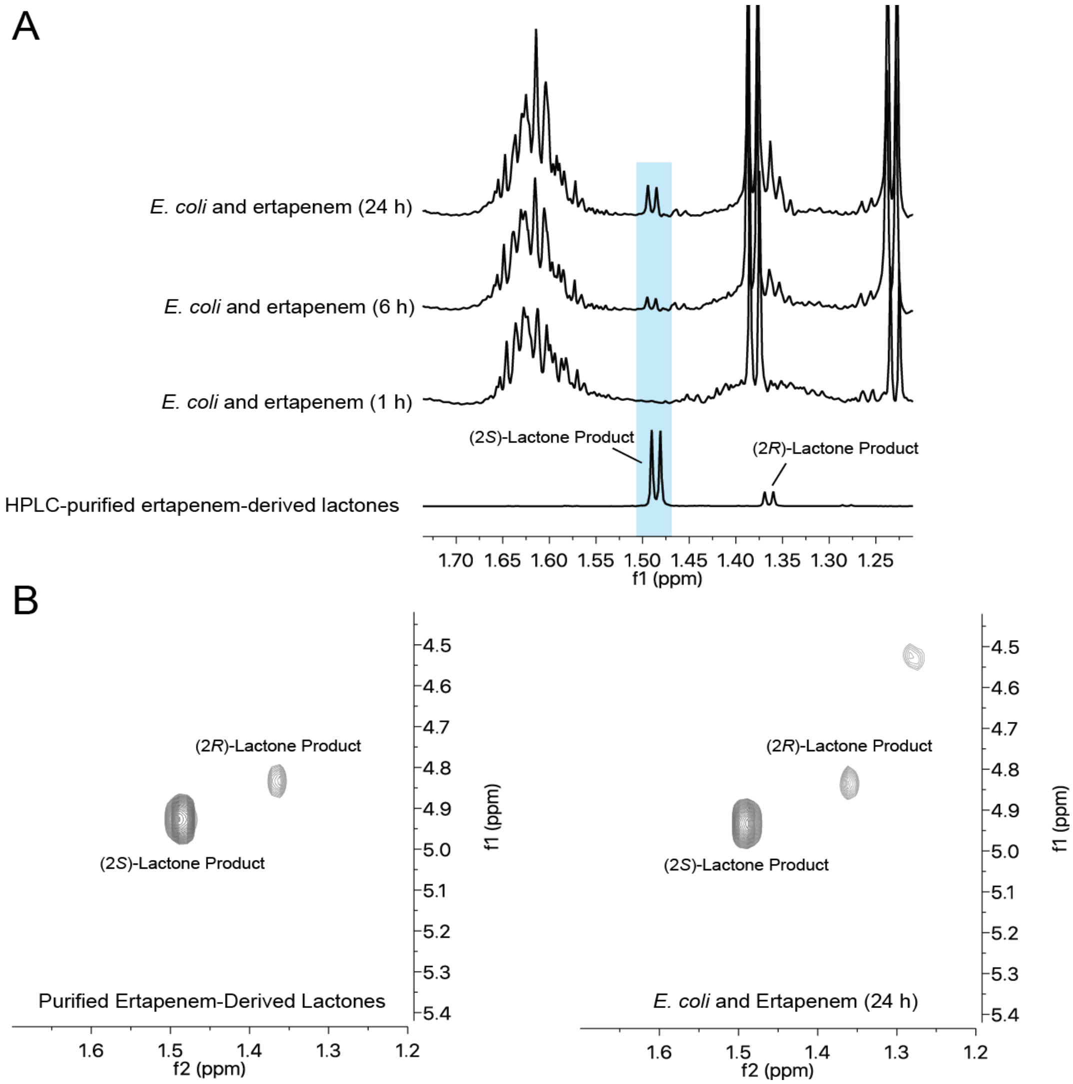

Figure S6. Formation of the ertapenem-derived lactones by *E. coli* expressing OXA-48. (A) ^{1}H NMR (700 MHz) time course monitoring the formation of the ertapenem-derived lactones by a culture of *Escherichia coli* BL21(DE3) expressing OXA-48 in the presence of 2 mM ertapenem, as compared to a sample of HPLC-purified ertapenem-derived lactones. (B) COSY spectra (700 MHz) comparing a culture of *E. coli* expressing OXA-48 treated with ertapenem (after 24 h) to an HPLC-purified sample of the ertapenem-derived lactones. *E. coli* BL21(DE3) transformed with pNIC28-OXA-48 was grown to an optical density of 0.6 in 2TY media supplemented with 50 μg/mL kanamycin, protein expression was induced with 0.5 mM isopropyl β-D-1-thiogalactopyranoside, and the culture was incubated at 18 °C, 180 rpm overnight. The culture was harvested, and the cell pellet was resuspended in a volume of fresh 2TY media corresponding to the original culture volume. The NMR sample consisted of the resuspended culture with 2 mM ertapenem in 2TY with 10 % D_2O. It is unclear whether the lactone products are formed within the *E. coli* cell (as the OXA-48 construct used is anticipated to produce protein, at least predominantly, in the cytoplasm), or results, in part, from the 'leakage' of OXA-48 into the culture medium (via cell lysis or otherwise).

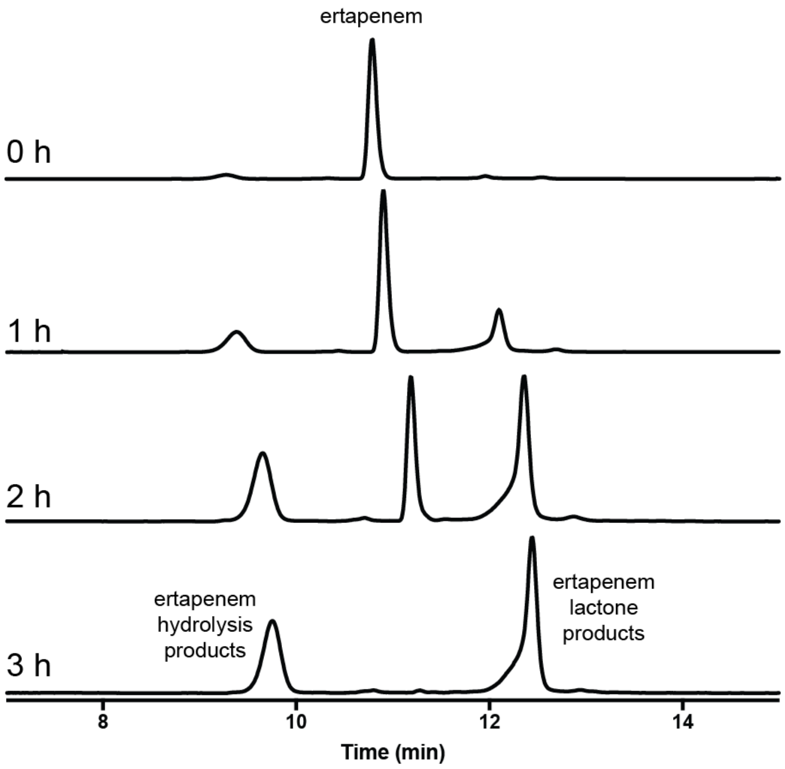

Figure S7. HPLC purification of the ertapenem-derived lactone and hydrolysis products. Samples from a mixture of 1 µM OXA-48 and 1 mM ertapenem (in 50 mM sodium phosphate, pH 7.5) were removed at the time points indicated, passed through an Amicon Ultra 0.5 mL centrifugal filter (3 kDa cut-off), and injected onto a C18 semi-preparative HPLC column. Further details are provided in the Experimental section. Note that the isolated hydrolysis and lactone products showed the presence of both (2*S*)- and (2*R*)-diastereomers by NMR spectroscopy (Figure S8, S12).

NMR spectra of ertapenem lactone products

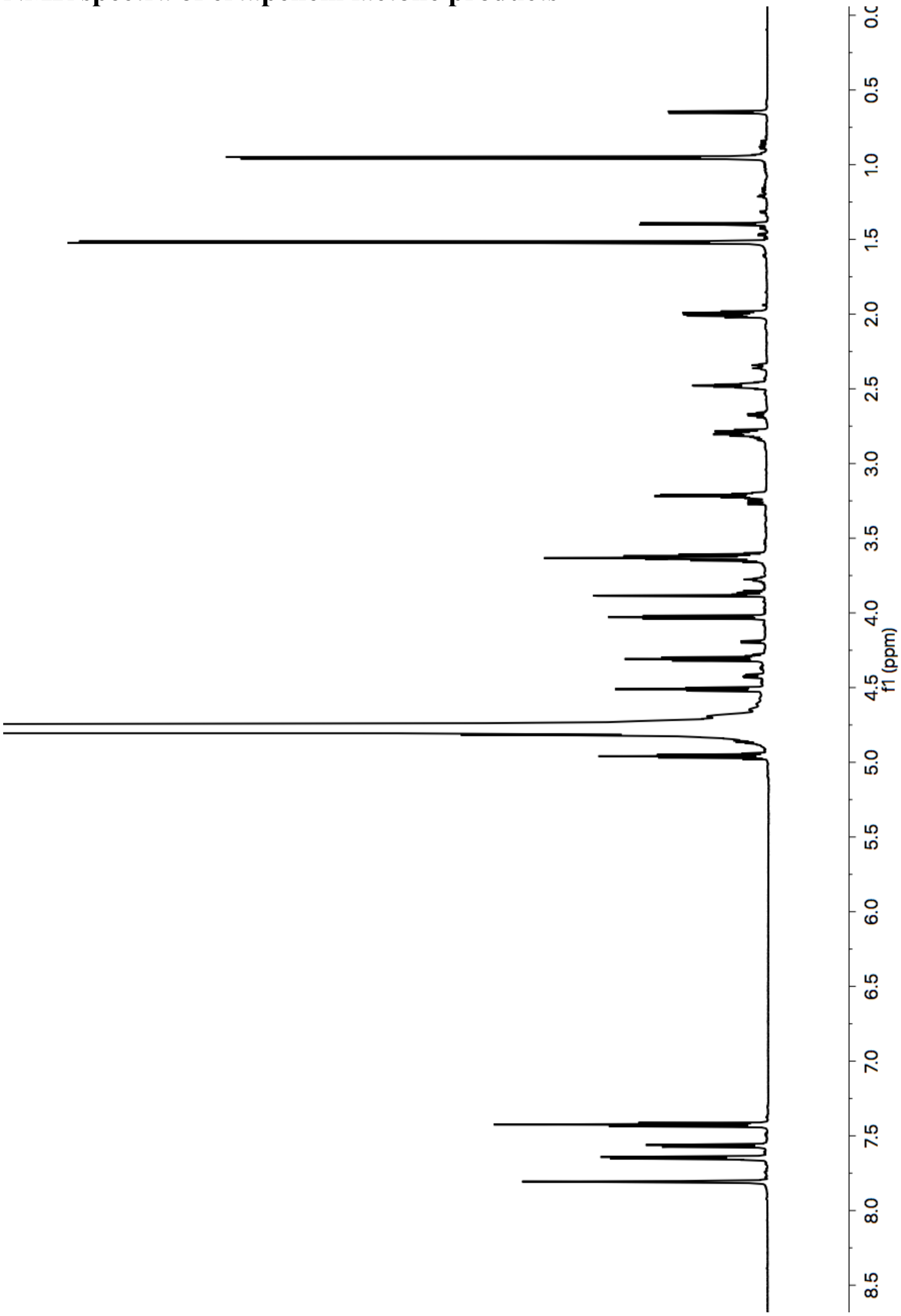

Figure S8. ^{1}H NMR spectrum of the ertapenem-derived lactone products. Sample consisted of 5 mM ertapenem lactone products (HPLC purified following treatment of ertapenem with OXA-48) in 50 mM sodium phosphate, pH 7.5, 10 % D_2O, and was measured on a 700 MHz NMR spectrometer. Note that the (2*S*)- and (2*R*)-diastereomers are both present, in an approximately 5:1 ratio (see Figure S4).

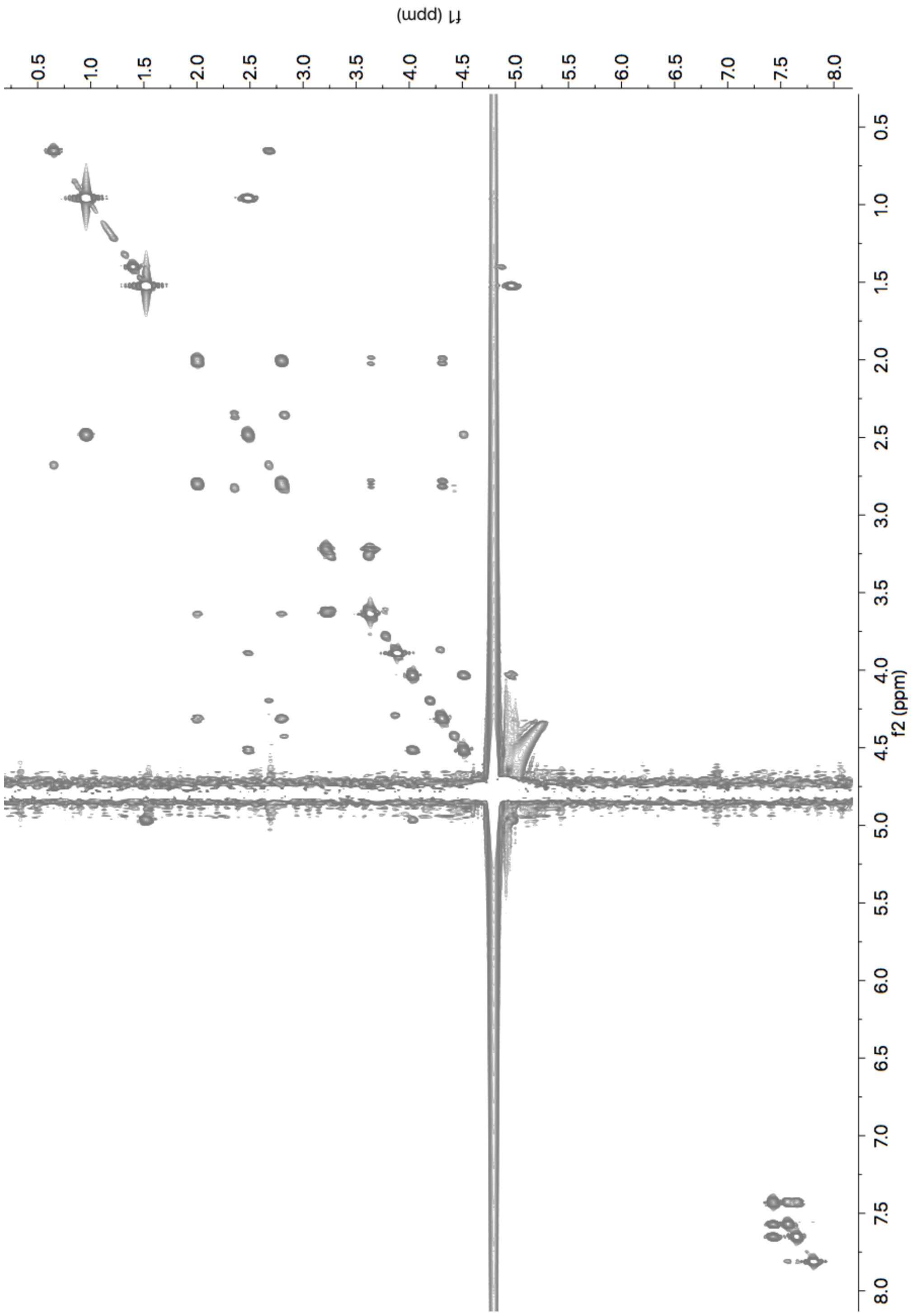

Figure S9. COSY spectrum of the ertapenem-derived lactone products. Sample consisted of 5 mM ertapenem lactone products (HPLC purified following treatment of ertapenem with OXA-48) in 50 mM sodium phosphate, pH 7.5, 10 % D_2O, and was measured on a 700 MHz NMR spectrometer. Note that the (2*S*)- and (2*R*)-diastereomers are both present, in an approximately 5:1 ratio (see Figure S4).

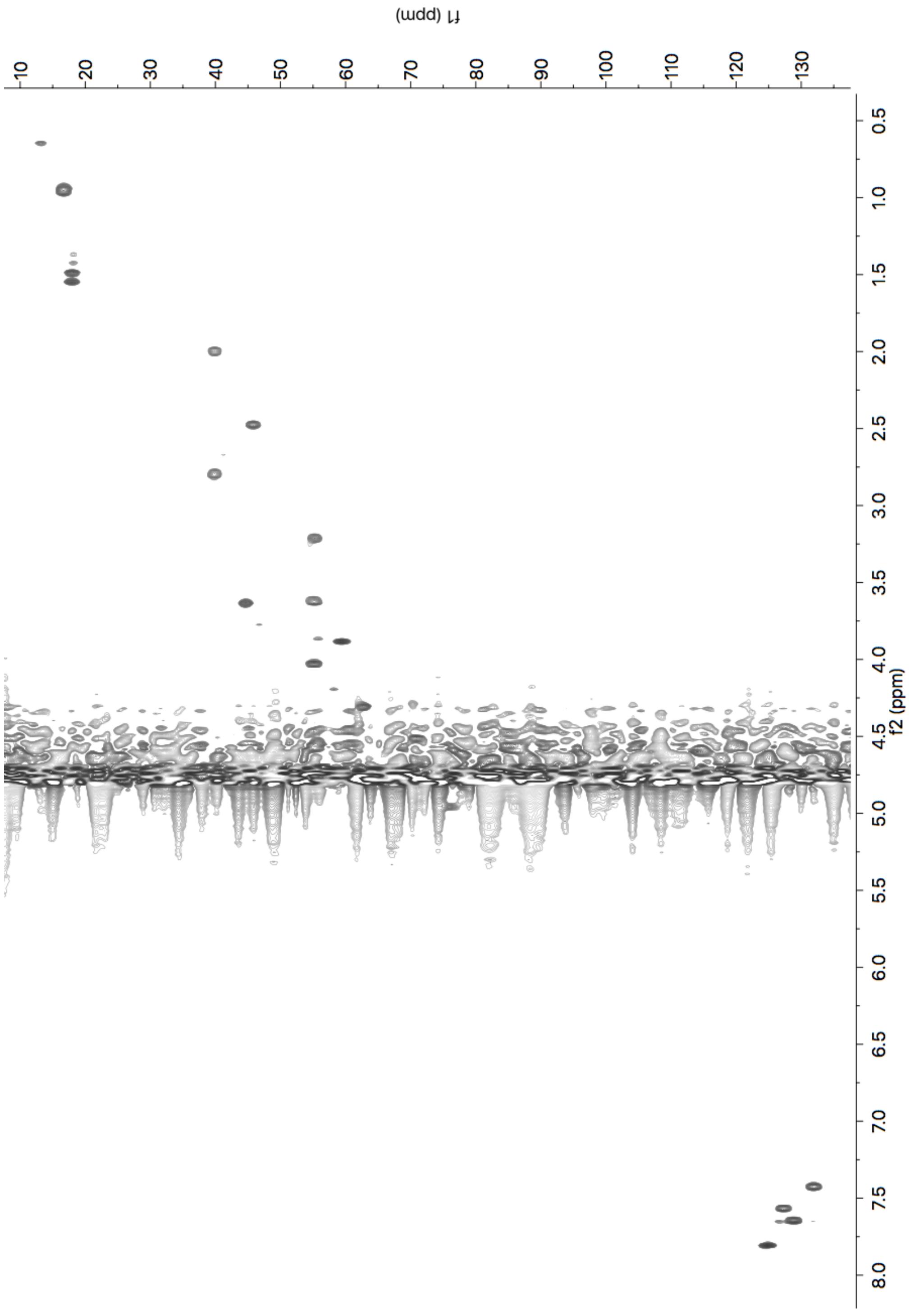

Figure S10. ^{1}H-^{13}C HSQC spectrum of the ertapenem-derived lactone products. Sample consisted of 5 mM ertapenem lactone products (HPLC purified following treatment of ertapenem with OXA-48) in 50 mM sodium phosphate, pH 7.5, 10 % D_2O, and was measured on a 700 MHz NMR spectrometer. Note that the (2*S*)- and (2*R*)-diastereomers are both present, in an approximately 5:1 ratio (see Figure S4).

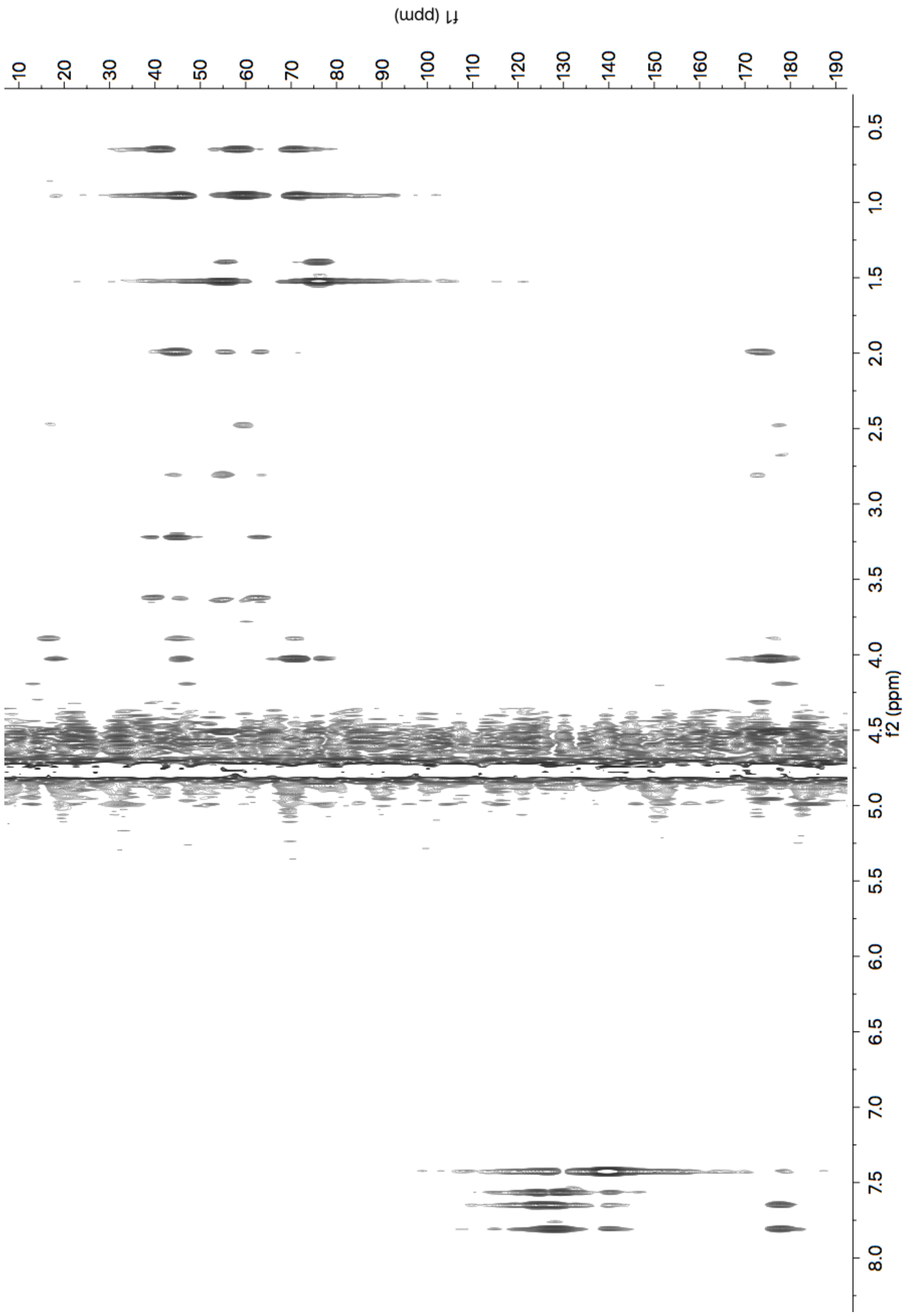

Figure S11. ^{1}H-^{13}C HMBC spectrum of the ertapenem-derived lactone products. Sample consisted of 5 mM ertapenem lactone products (HPLC purified following treatment of ertapenem with OXA-48) in 50 mM sodium phosphate, pH 7.5, 10 % D_2O, and was measured on a 700 MHz NMR spectrometer. Note that the (2*S*)- and (2*R*)-diastereomers are both present, in an approximately 5:1 ratio (see Figure S4).

NMR spectra of ertapenem hydrolysis products

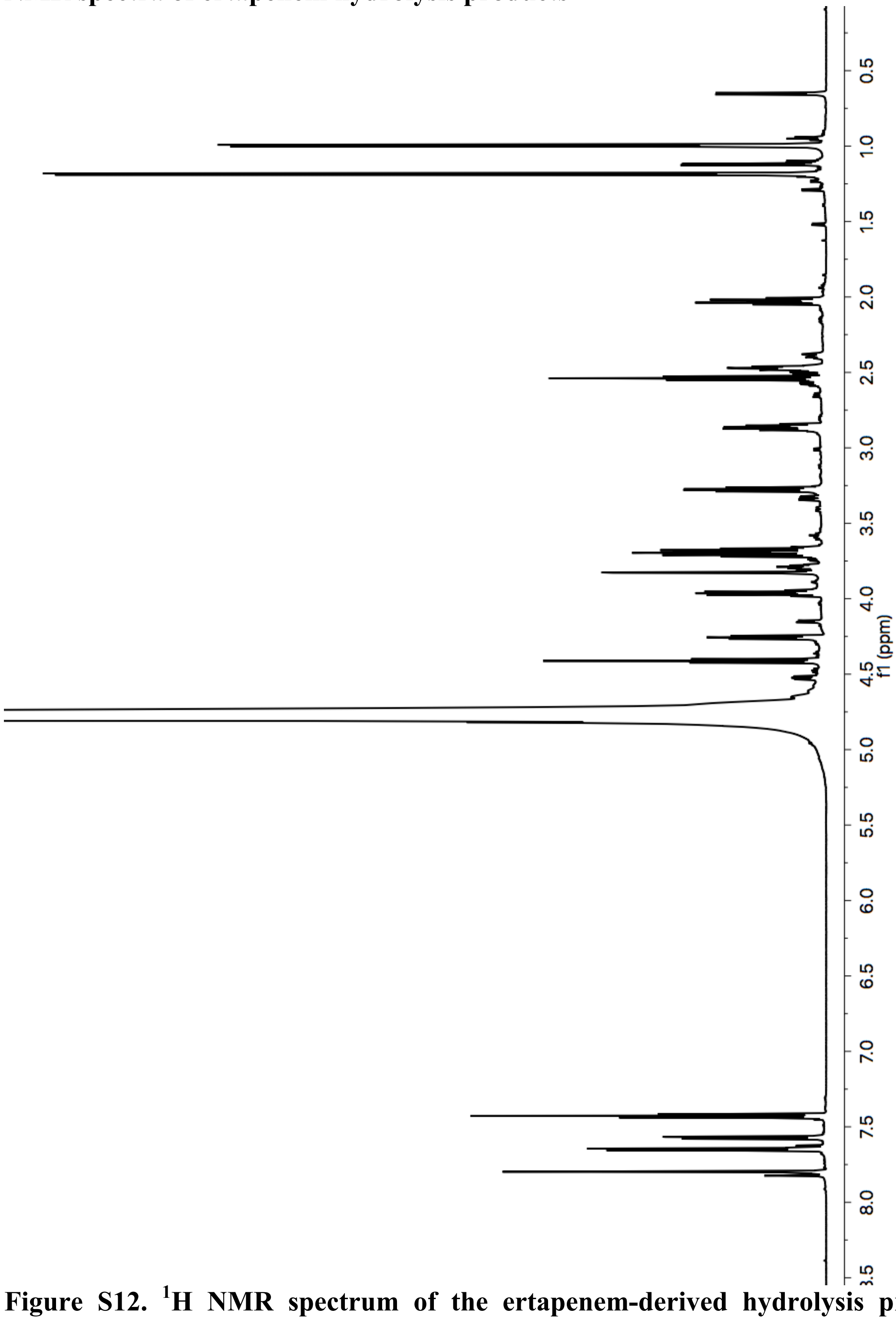

Figure S12. ^{1}H NMR spectrum of the ertapenem-derived hydrolysis products. Sample consisted of 5 mM ertapenem hydrolysis products (HPLC purified following treatment of ertapenem with OXA-48) in 50 mM sodium phosphate, pH 7.5, 10 % D_2O, and was measured on a 700 MHz NMR spectrometer. Note that the (2*S*)- and (2*R*)-diastereomers are both present, in an approximately 5:1 ratio (see Figure S2).

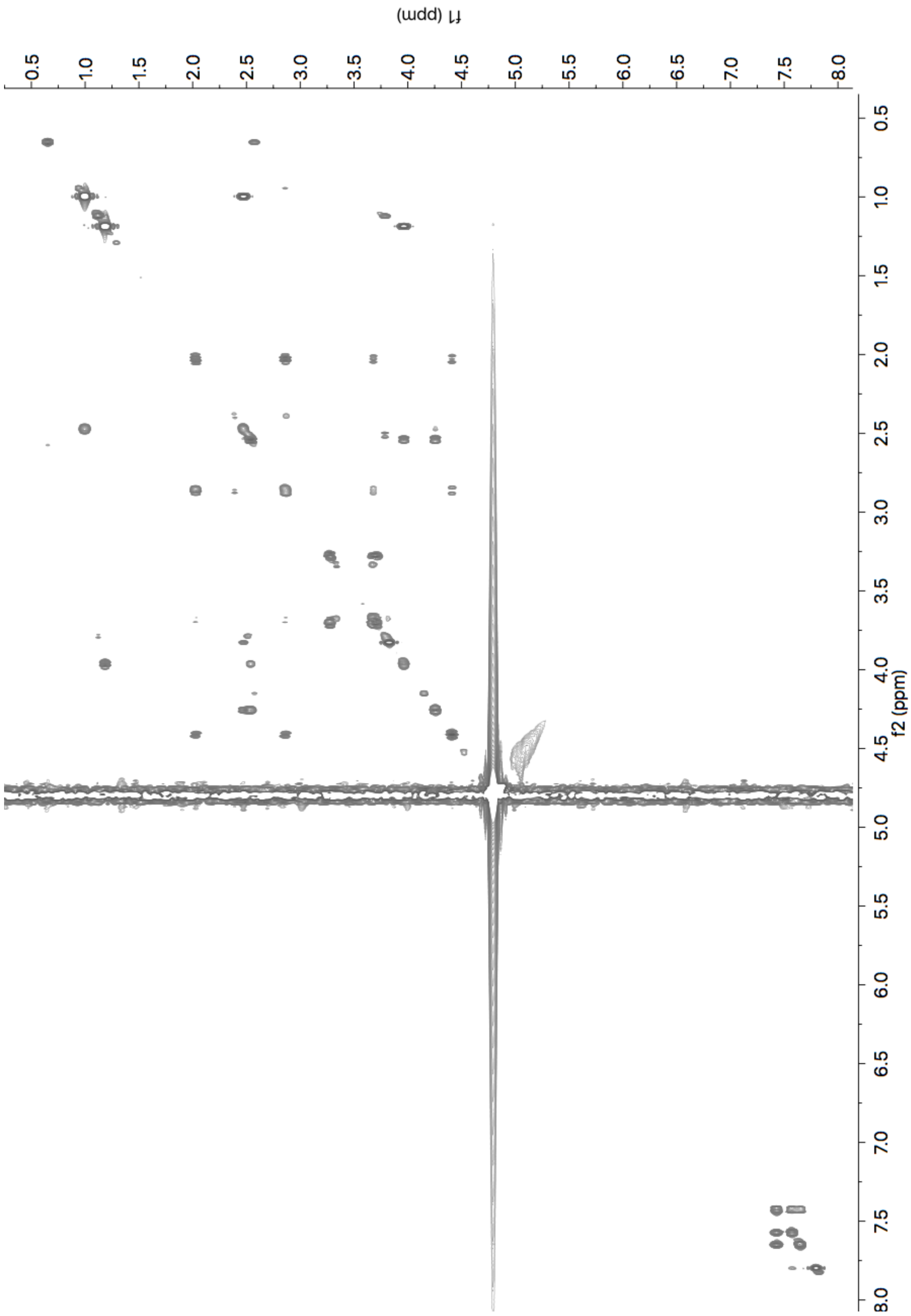

Figure S13. COSY spectrum of the ertapenem-derived hydrolysis products. Sample consisted of 5 mM ertapenem hydrolysis products (HPLC purified following treatment of ertapenem with OXA-48) in 50 mM sodium phosphate, pH 7.5, 10 % D_2O, and was measured on a 700 MHz NMR spectrometer. Note that the (2*S*)- and (2*R*)-diastereomers are both present, in an approximately 5:1 ratio (see Figure S2).

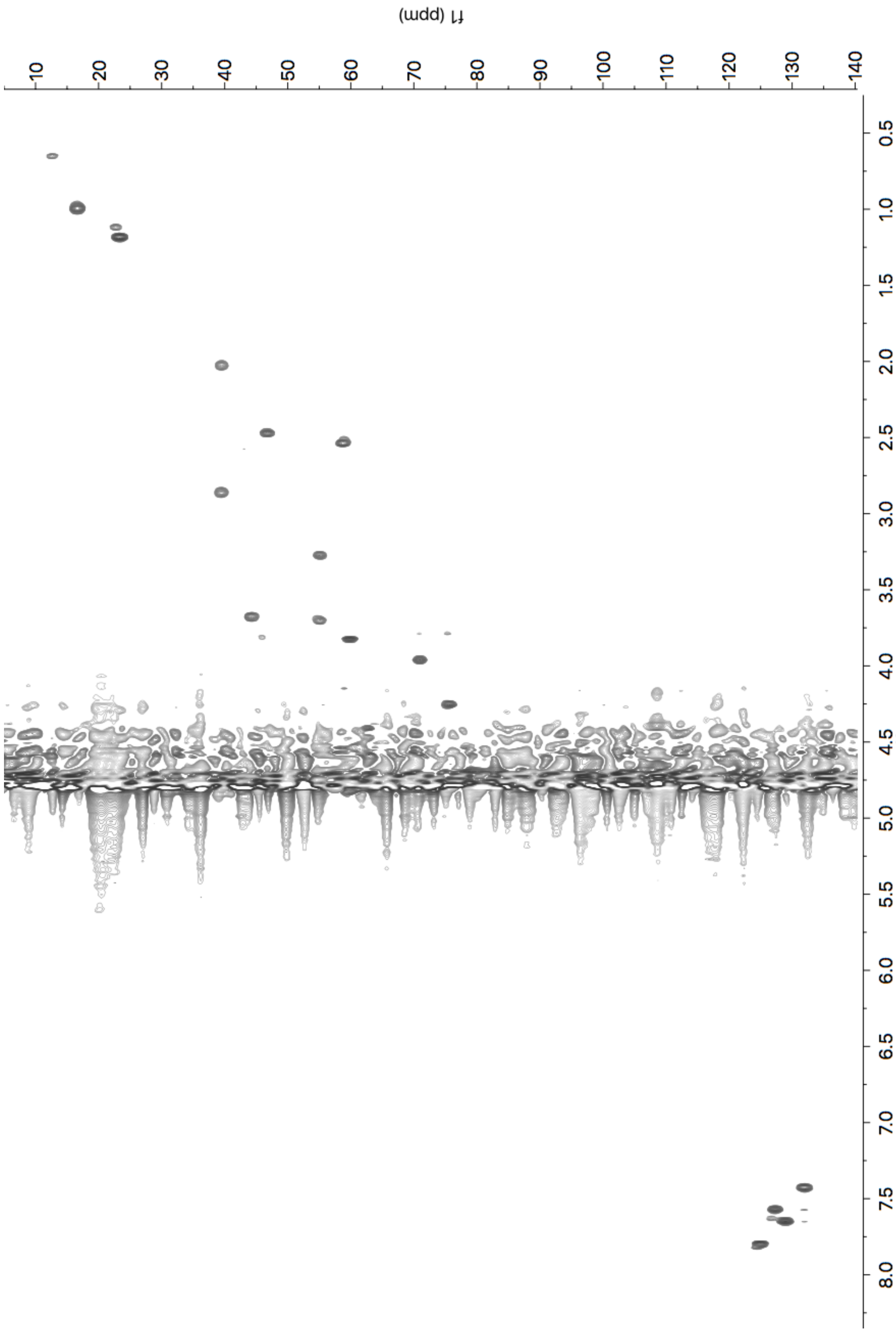

Figure S14. ^{1}H-^{13}C HSQC spectrum of the ertapenem-derived hydrolysis products. Sample consisted of 5 mM ertapenem hydrolysis products (HPLC purified following treatment of ertapenem with OXA-48) in 50 mM sodium phosphate, pH 7.5, 10 % D_2O, and was measured on a 700 MHz NMR spectrometer. Note that the (2*S*)- and (2*R*)-diastereomers are both present, in an approximately 5:1 ratio (see Figure S2).

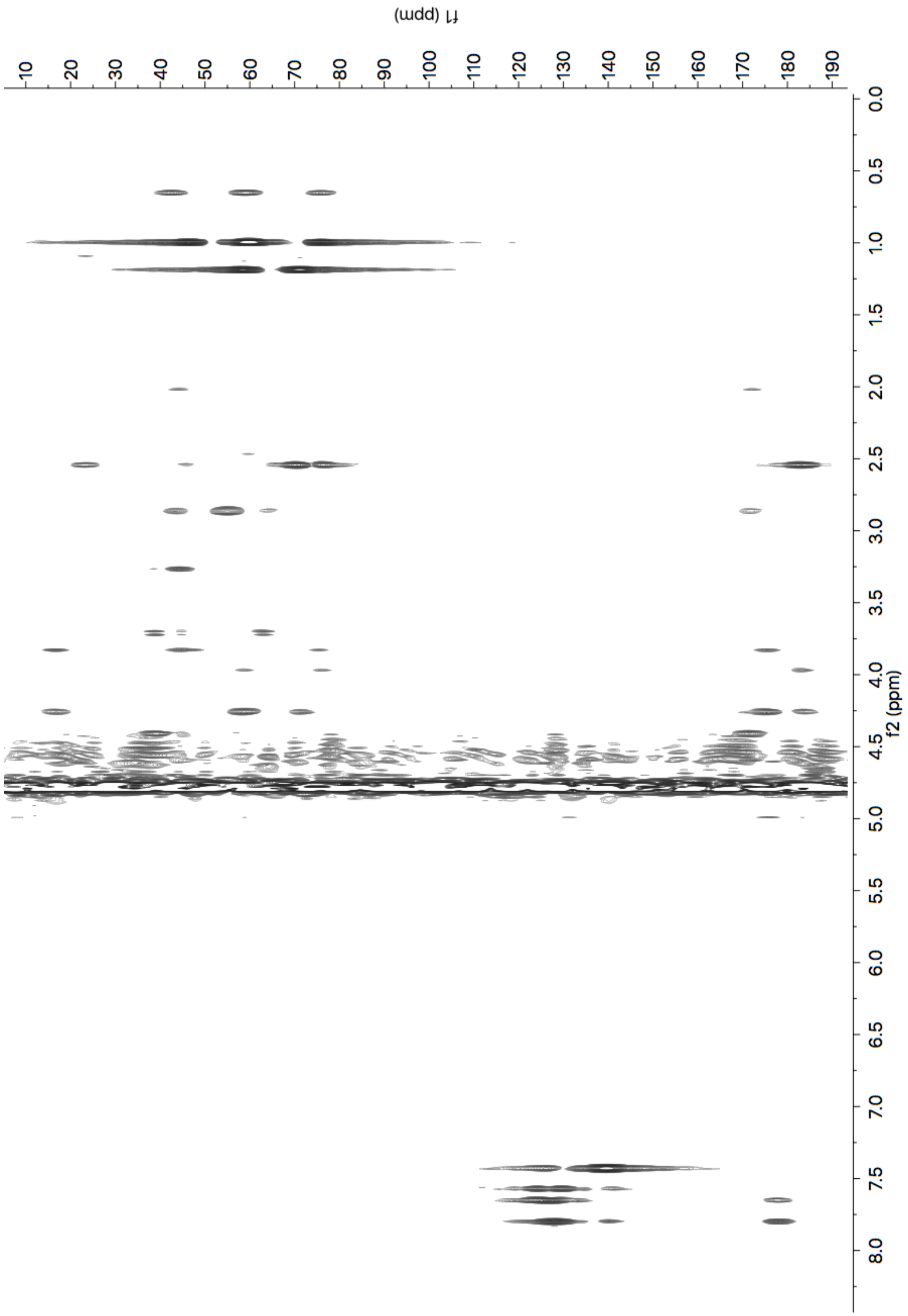

Figure S15. ^{1}H-^{13}C HMBC spectrum of the ertapenem-derived hydrolysis products. Sample consisted of 5 mM ertapenem hydrolysis products (HPLC purified following treatment of ertapenem with OXA-48) in 50 mM sodium phosphate, pH 7.5, 10 % D_2O, and was measured on a 700 MHz NMR spectrometer. Note that the (2*S*)- and (2*R*)-diastereomers are both present, in approximately a 5:1 ratio (see Figure S2).

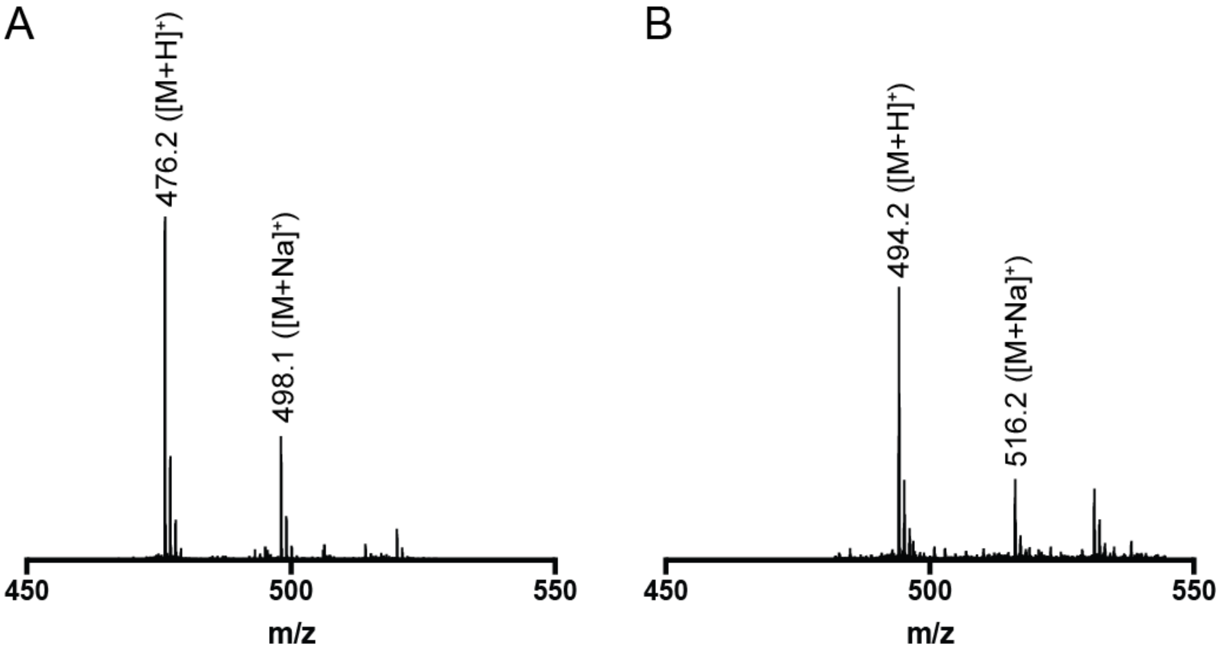

Figure S16. Mass spectra of the ertapenem-derived lactone and hydrolysis products. ESI mass spectra of HPLC-purified samples (Figure S7) of the ertapenem-derived (A) lactone and (B) hydrolysis products. Calculated mass of the (unprotonated) ertapenem-derived lactone: 475.1 Da. Calculated mass of the (unprotonated) ertapenem hydrolysis product: 493.2 Da. Compound structures are shown in Figure 1.

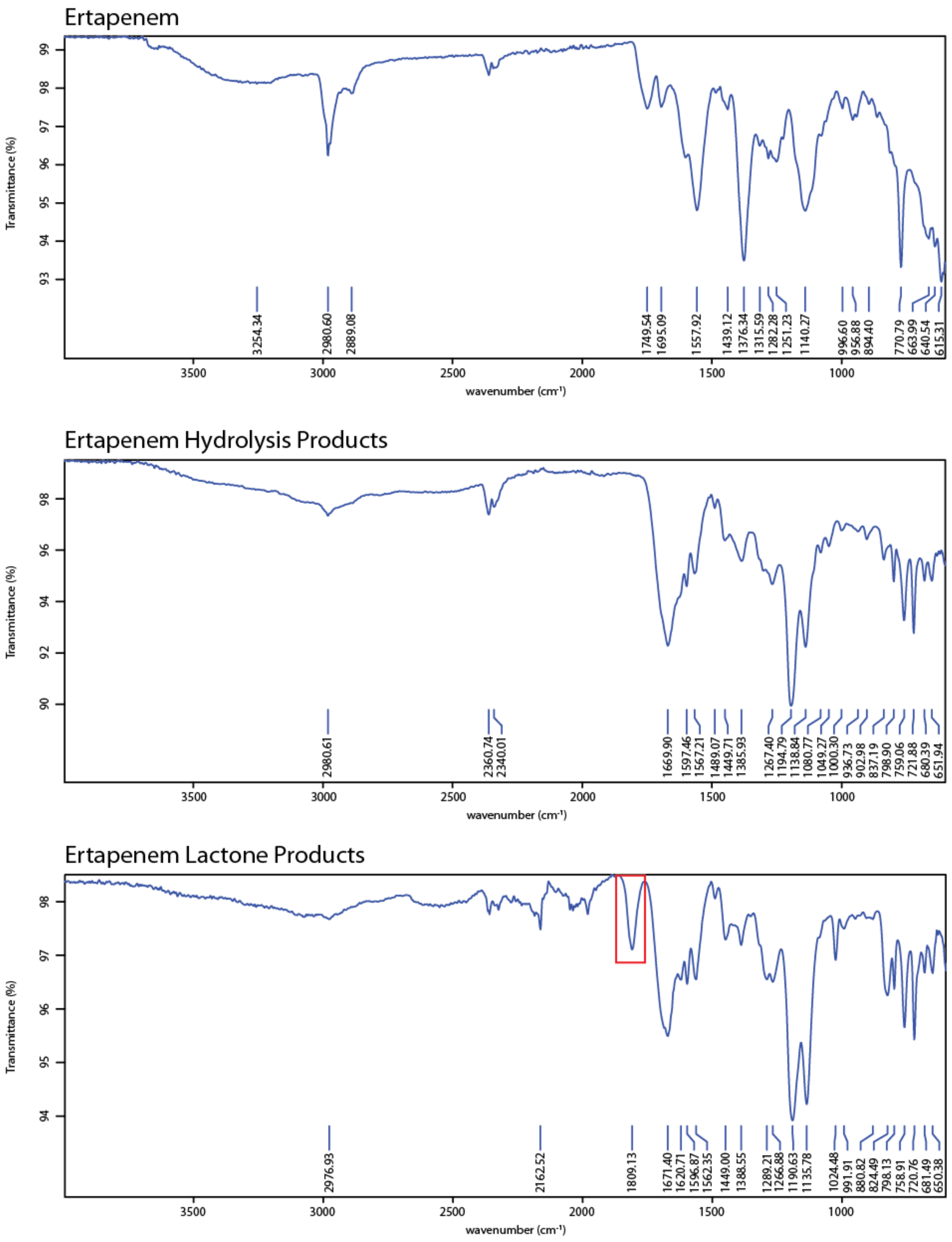

Figure S17. Infrared spectra of ertapenem and ertapenem products. The infrared spectra were measured using the HPLC-purified products of ertapenem and OXA-48. The peak at 1809.13 cm^{-1} (indicated in the red box) is in a similar range as the carbonyl stretch observed for other β-lactones.[14,16]

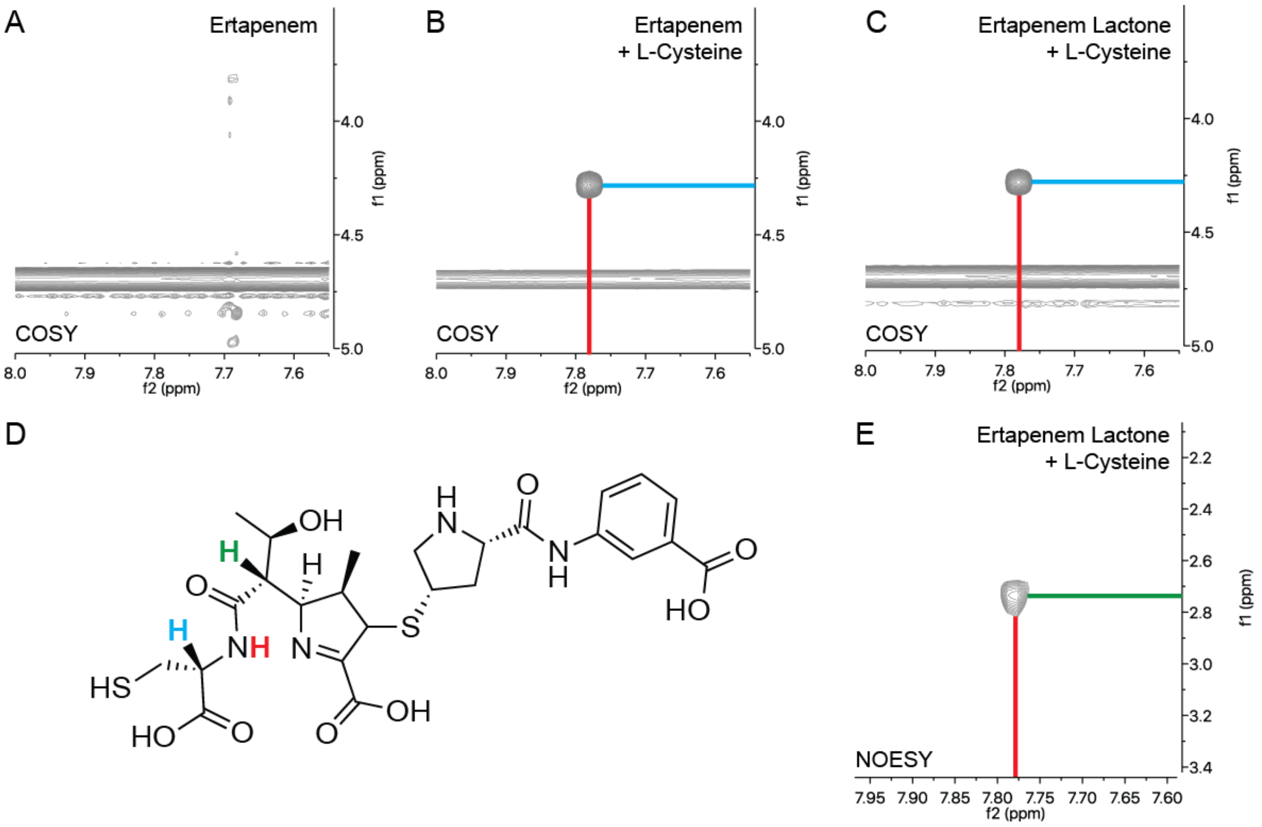

Figure S18. Reaction of the ertapenem-derived lactone with L-cysteine. Partial COSY spectra (700 MHz) of (A) ertapenem, (B) a mixture of ertapenem and L-cysteine, and (C) a mixture of OXA-48, ertapenem, and L-cysteine (full consumption of ertapenem was confirmed prior to the addition of L-cysteine). When L-cysteine was added to ertapenem or the ertapenem-derived lactone, a peak corresponding to an amide proton appeared at 7.8 ppm (red), which showed a correlation to the cysteine α-proton at 4.25 ppm (blue) via COSY. (D) Proposed structure of the cysteine ertapenem adduct (which likely results from the intramolecular S- to N-acyl shift of the initial thioester product).[17] (E) NOESY spectrum showing a correlation between the cysteine amide proton (red) and the α-proton of the amide (2.7 ppm; green). This α-proton showed correlations to the hydroxyethyl side chain and the pyrroline ring via a TOCSY experiment. Chemical shift assignments for the adduct are provided in Table S7.

Table S7. Chemical shift assignments for the product derived from the reaction of L-cysteine with ertapenem.

Position	^{1}H (ppm)
1	2.51
2	3.86
3	
4	
5	4.30
6	2.77
7	
8	4.05
9	1.20
10	
11	0.99
12	3.13, 3.55
13	3.57
14	1.94, 2.77
15	4.22
16	
17	
18	7.79
19	
20	7.64
21	7.42
22	7.57
23	
24	7.78
25	4.28
26	
27	2.76, 2.83

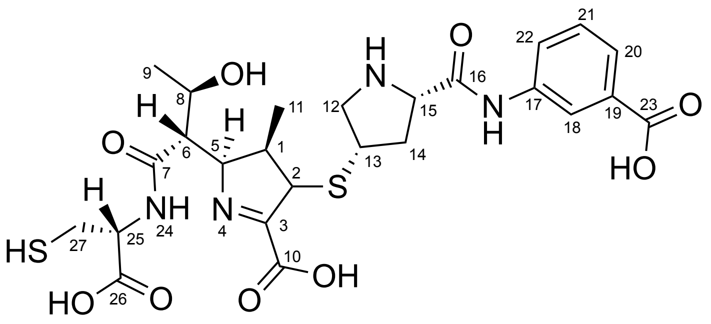

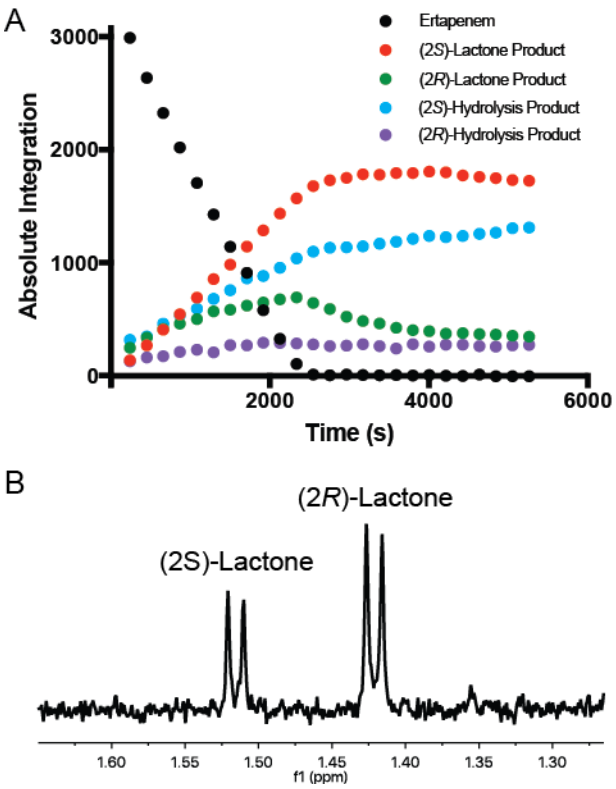

Figure S19. OXA-48 ertapenem NMR time course. (A) A mixture of OXA-48 (5 µM) and ertapenem (1 mM) was prepared in 50 mM sodium phosphate, pH 7.5, 10 % D_2O. Product formation was monitored by NMR (600 MHz), with proton spectra (32 scans) acquired every 3.5 min for 90 min. Note that the OXA-23 ertapenem time course appears similar to that of OXA-48, while the degradation of ertapenem by OXA-10 occurs much more slowly. (B) After mixing OXA-48 (10 µM) with ertapenem (1 mM), a 1H NMR spectrum was acquired as quickly as possible (after approx. 3 min). These data raise the possibility that the (2*R*)-lactone product is formed first, and that the (2*S*)-lactone product (which predominates at later time points) may be formed non-enzymatically.

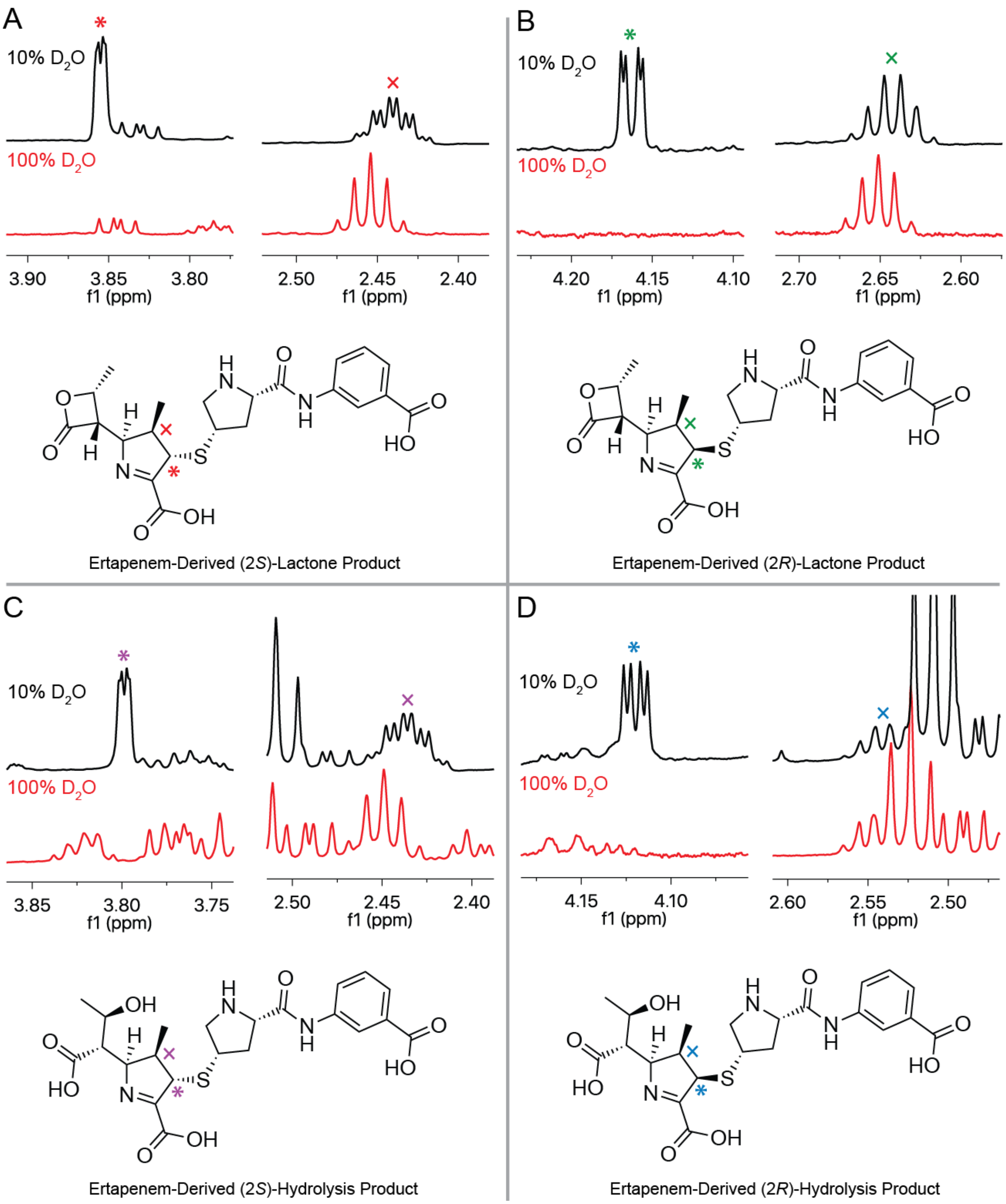

Figure S20. Exchange of ertapenem product C-2 protons with solvent. NMR spectra (700 MHz) of the HPLC-purified (A, B) ertapenem-derived lactone products and the (C, D) ertapenem-derived hydrolysis products. Spectra were measured in 50 mM phosphate buffer, pH 7.5, either with 10 % D_2O (black) or ~100 % D_2O. The proton on C-2 (indicated with *) is lost in ~100 %

D_2O, while the coupling pattern of the proton on C-3 (indicated with ×) indicates the loss of the signal for an adjacent proton.

NMR spectra of carbapenem/carbapenemase reaction mixtures

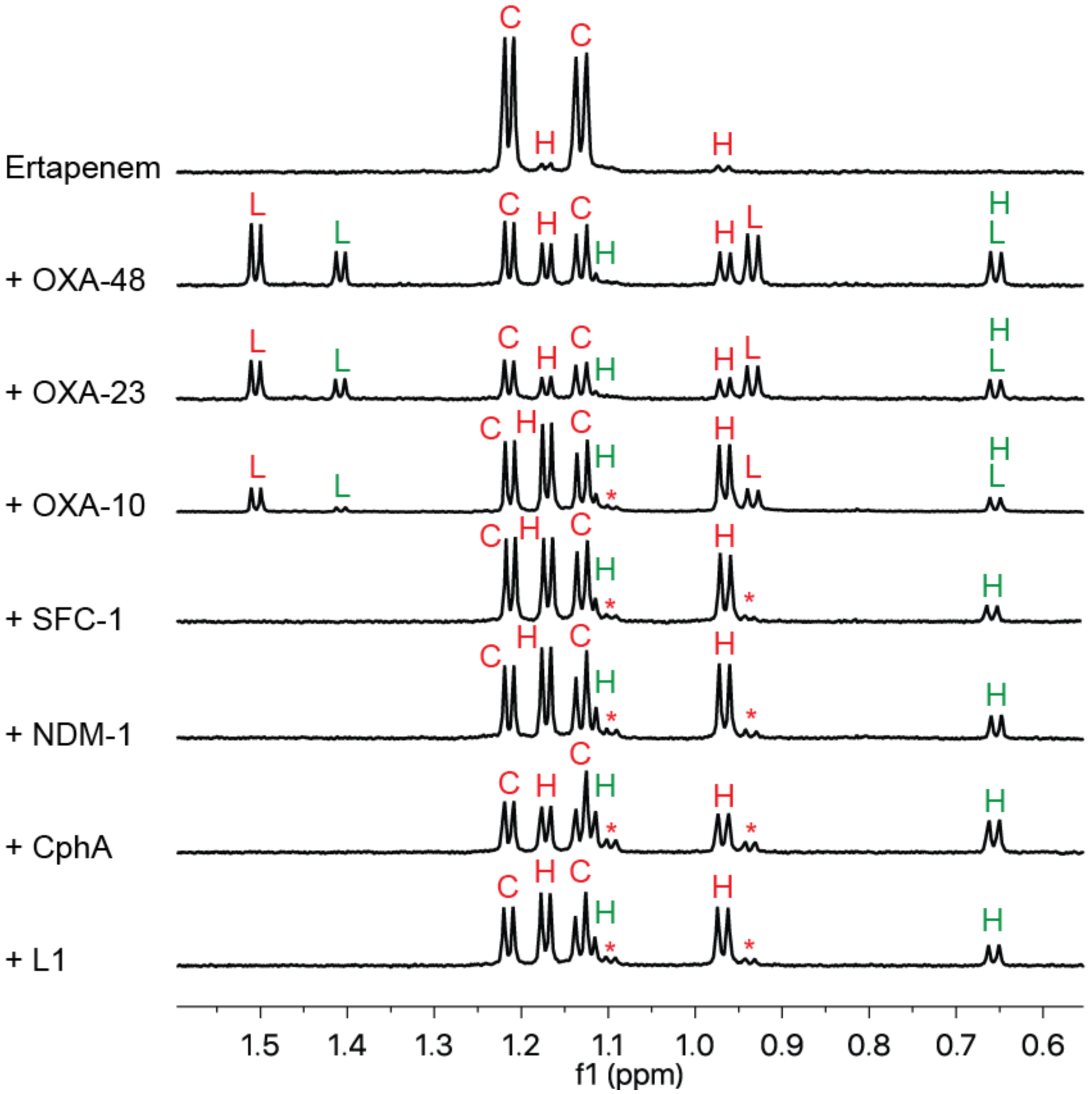

Figure S21. Product profile of ertapenem with carbapenemases. ^{1}H-NMR spectra (600 MHz) showing the products formed from ertapenem (1 mM) with OXA-48 (5 µM), OXA-23 (5 µM), OXA-10 (10 µM), SFC-1 (50 nM), NDM-1 (5 µM), CphA (250 nM), and L1 (100 nM). Reactions were carried out in 50 mM sodium phosphate, pH 7.5, 10 % D_2O. The major carbapenem (C), lactone (L), and hydrolysis (H) products are indicated with red letters (see Tables S1, S2, S4 for structures), minor lactone (L) and hydrolysis (H) products are indicated with green letters (see Tables S3 and S5 for structures), and uncharacterised peaks are indicated with asterisks. Signals corresponding to the ertapenem-derived lactone products were only observed for OXA-10, OXA-23, and OXA-48. In some cases, we observed minor resonances in the methyl group region of the spectrum, which we could not assign due to their low levels. These may reflect enamine tautomeric forms of the assigned imine forms of the hydrolysis and/or lactone products. The spectra were acquired after 30 min (OXA-48), 28 min (OXA-23), 8 h (OXA-10), 22 min (SFC-1), 10 min (NDM-1), 5 min (CphA), and 10 min (L1). Note that the ratios of stereoisomers observed are time-dependent (see Figure S20).

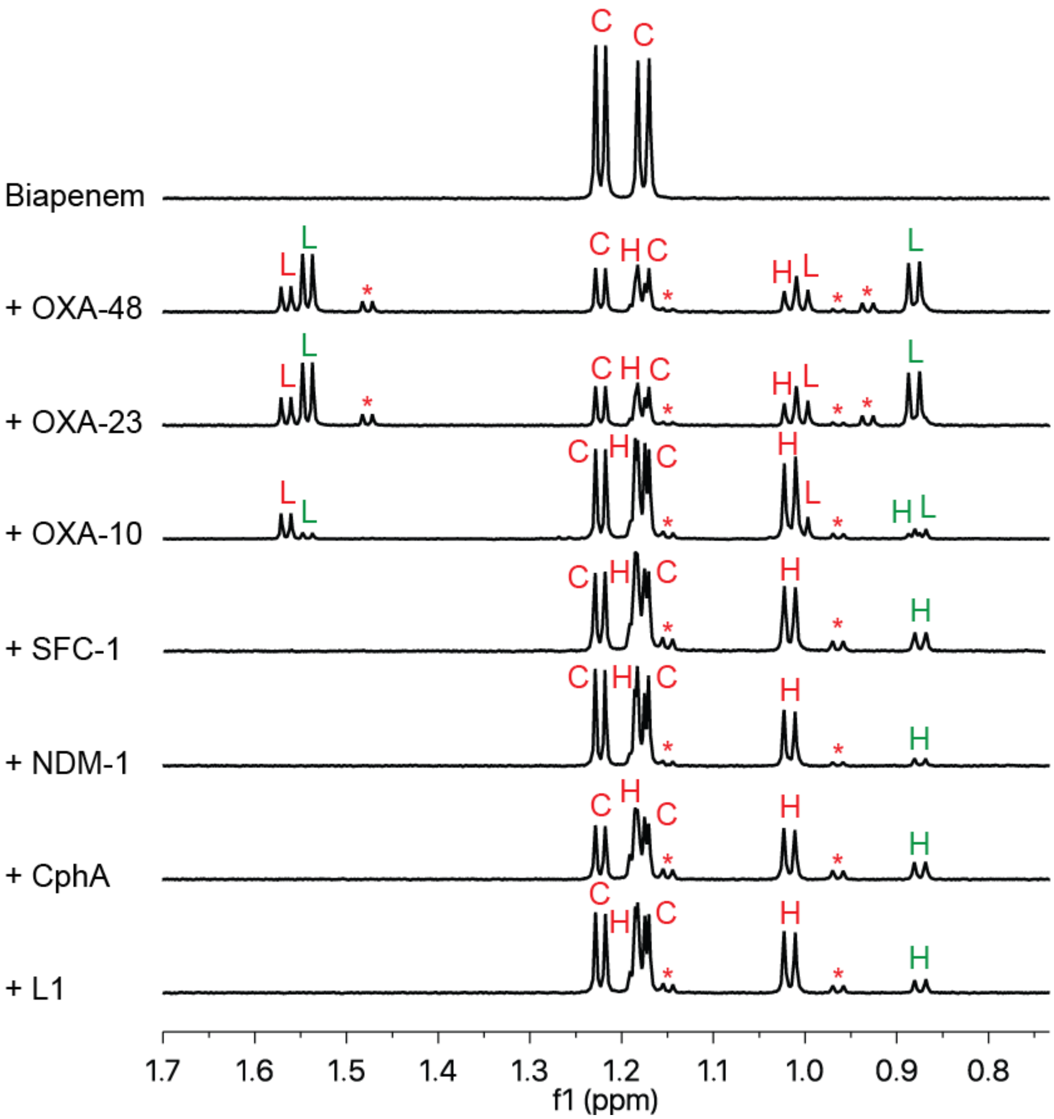

Figure S22. Product profile of biapenem with carbapenemases. ^{1}H-NMR spectra (600 MHz) showing the products formed from biapenem (1 mM) with OXA-48 (5 µM), OXA-23 (5 µM), OXA-10 (10 µM), SFC-1 (250 nM), NDM-1 (5 µM), CphA (25 nM), and L1 (200 nM). Reactions were carried out in 50 mM sodium phosphate, pH 7.5, 10 % D_2O. The major carbapenem (C), lactone (L), and hydrolysis (H) products are indicated with red letters (see Tables S8-S10 for structures), minor lactone (L) and hydrolysis (H) products are indicated with green letters, and uncharacterised peaks are indicated with asterisks. Signals corresponding to the biapenem-derived lactone products were only observed for OXA-10, OXA-23, and OXA-48. The spectra were acquired after 15 min (OXA-48), 15 min (OXA-23), 2 h (OXA-10), 8 min (SFC-1), 38 min (NDM-1), 5 min (CphA), and 7 min (L1). Note that the ratios of stereoisomers observed are time-dependent (see Figure S20).

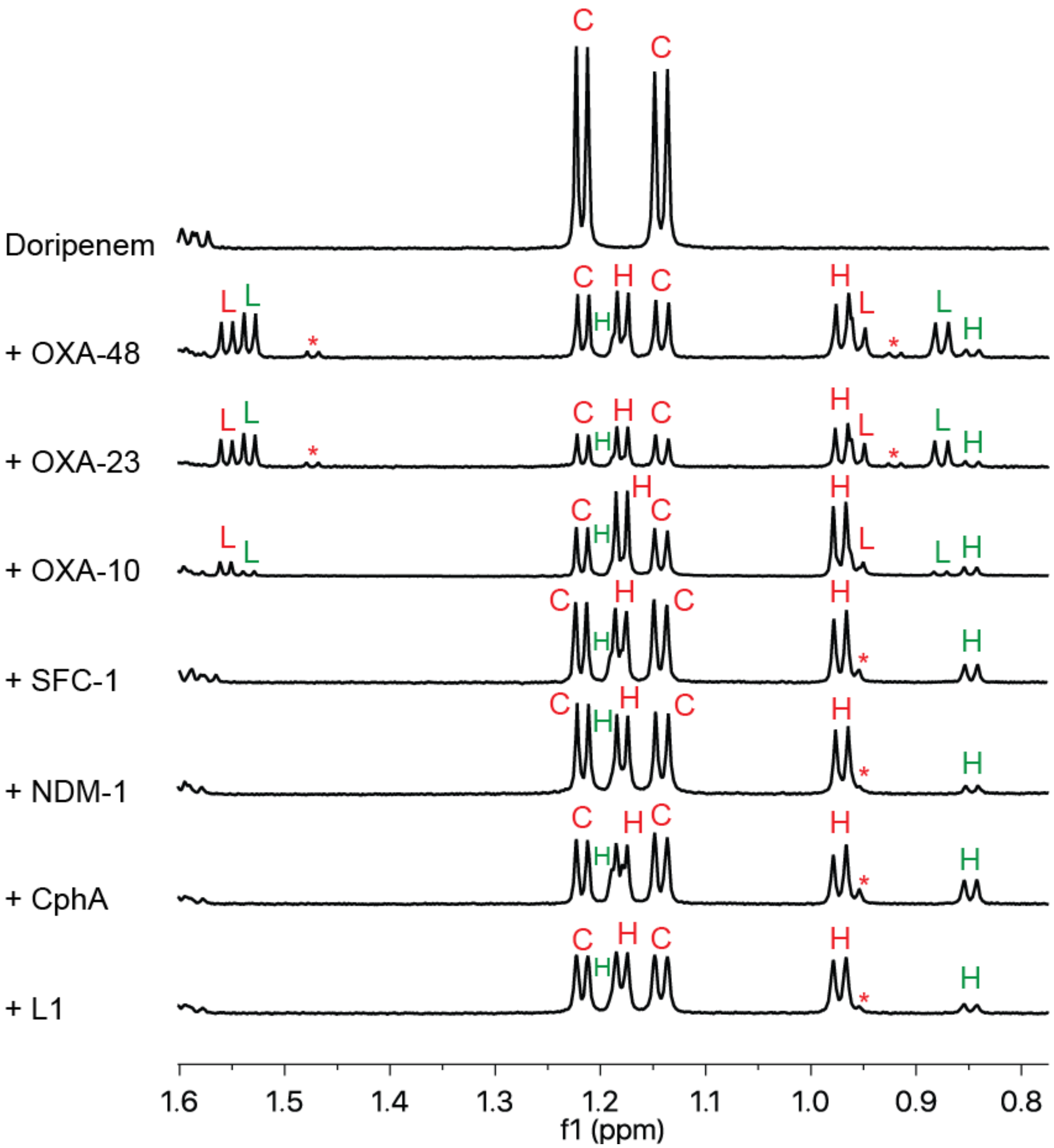

Figure S23. Product profile of doripenem with carbapenemases. ^{1}H-NMR spectra (600 MHz) showing the products formed from doripenem (1 mM) with OXA-48 (5 µM), OXA-23 (5 µM), OXA-10 (10 µM), SFC-1 (500 nM), NDM-1 (5 µM), CphA (25 nM), and L1 (400 nM). Reactions were carried out in 50 mM sodium phosphate, pH 7.5, 10 % D_2O. The major carbapenem (C), lactone (L), and hydrolysis (H) products are indicated with red letters (see Tables S11-S13 for structures), minor lactone (L) and hydrolysis (H) products are indicated with green letters, and uncharacterised peaks are indicated with asterisks. Signals corresponding to the doripenem-derived lactone products were only observed for OXA-10, OXA-23, and OXA-48. The spectra were acquired after 50 min (OXA-48), 50 min (OXA-23), 2.5 h (OXA-10), 10 min (SFC-1), 38 min (NDM-1), 5 min (CphA), and 10 min (L1). Note that the ratios of stereoisomers observed are time-dependent (see Figure S20).

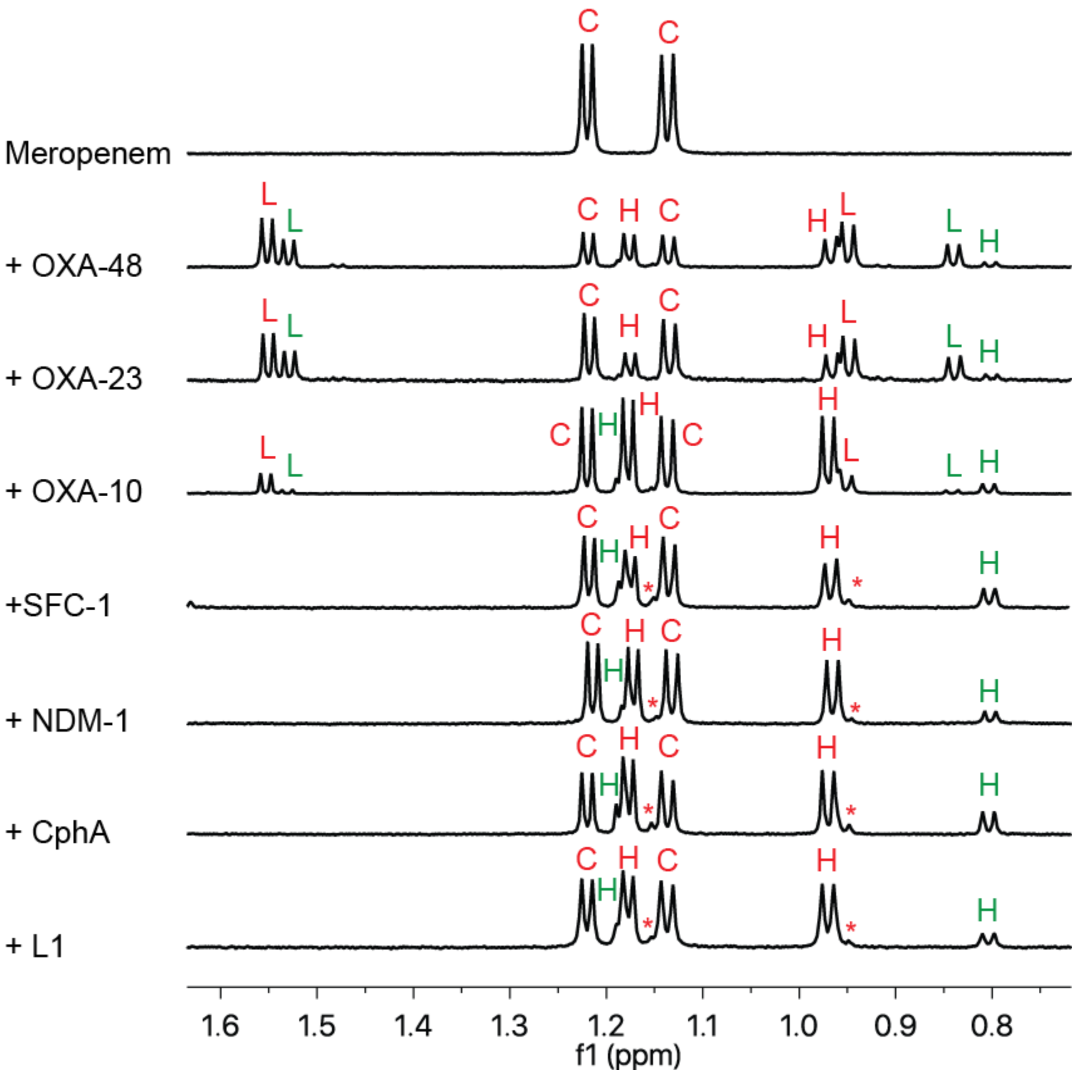

Figure S24. Product profile of meropenem with carbapenemases. ^{1}H-NMR spectra (600 MHz) showing the products formed from meropenem (1 mM) with OXA-48 (5 µM), OXA-23 (5 µM), OXA-10 (10 µM), SFC-1 (250 nM), NDM-1 (5 µM), CphA (250 nM), and L1 (100 nM). Reactions were carried out in 50 mM sodium phosphate, pH 7.5, 10 % D_2O. The major carbapenem (C), lactone (L), and hydrolysis (H) products are indicated with red letters (see Tables S14-S16 for structures), minor lactone (L) and hydrolysis (H) products are indicated with green letters, and uncharacterised peaks are indicated with asterisks. Signals corresponding to the meropenem-derived lactone products were only observed for OXA-10, OXA-23, and OXA-48. The spectra were acquired after 40 min (OXA-48), 40 min (OXA-23), 1 h (OXA-10), 8 min (SFC-1), 20 min (NDM-1), 7 min (CphA), and 12 min (L1). Note that the ratios of stereoisomers observed are time-dependent (see Figure S20).

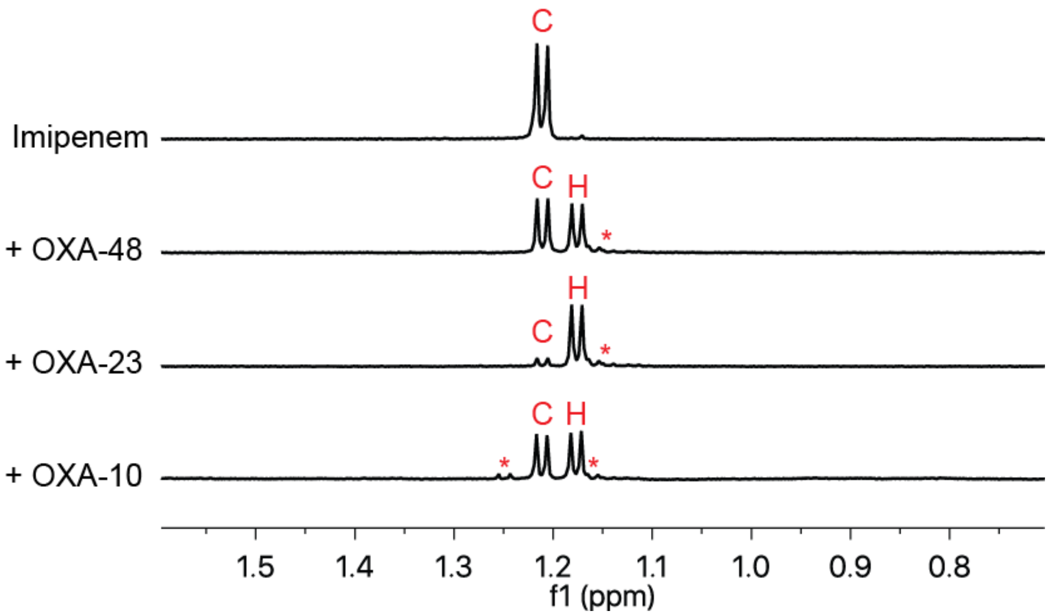

Figure S25. Product profile of imipenem with class D β-lactamases. ^{1}H-NMR spectra (600 MHz) showing the products formed from imipenem (1 mM) with OXA-48 (125 nM), OXA-23 (500 nM), and OXA-10 (60 µM). Reactions were carried out in 50 mM sodium phosphate, pH 7.5, 10 % D_2O. The major carbapenem (C) and hydrolysis (H) products are indicated with red letters (see Tables S17, S18 for structures), and uncharacterised peaks are indicated with asterisks. No signals were observed in the region expected for the corresponding lactone product. The spectra were acquired after 9 min (OXA-48), 5 min (OXA-23), and 25 min (OXA-10). Note that the ratios of stereoisomers observed are time-dependent (see Figure S20).

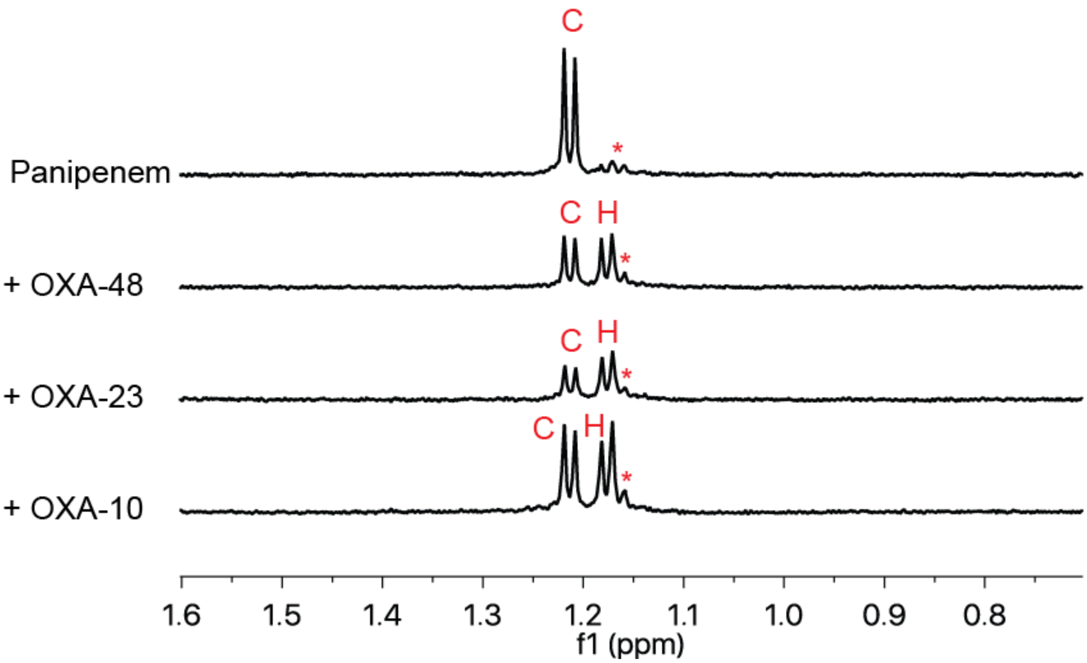

Figure S26. Product profile of panipenem with class D β-lactamases. ^{1}H-NMR spectra (600 MHz) showing the products formed from panipenem (1 mM) with OXA-48 (500 nM), OXA-23 (5 µM), and OXA-10 (10 µM). Reactions were carried out in 50 mM sodium phosphate, pH 7.5, 10 % D_2O. The major carbapenem (C) and hydrolysis (H) products are indicated with red letters (see Tables S19, S20 for structures), and uncharacterised peaks are indicated with asterisks. No signals were observed in the region expected for the corresponding lactone product. The spectra were acquired after 20 min (OXA-48), 5 min (OXA-23), and 32 min (OXA-10). Note that the ratios of stereoisomers are time-dependent (see Figure S20).

Table S8. Chemical shift assignments for biapenem.

Position	^{13}C (ppm)[a]	^{1}H (ppm)
1	45.06	3.34
2	136.94	
3		
4		
5	58.55	4.24
6	61.72	3.46
7	179.13	
8	67.68	4.20
9	18.29	1.23
10		
11	22.68	1.19
12, 14	54.91, 57.08	4.69, 5.00, 5.04
13	47.62	4.92
15, 16	146.2	8.96, 8.98

[a]Spectra are shown in Figures S43-S46.

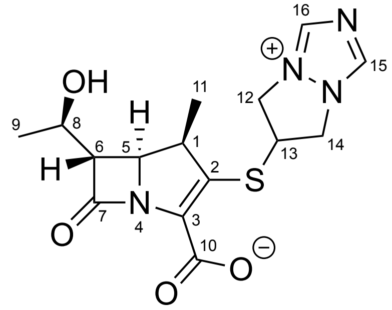

Table S9. Chemical shift assignments for the major biapenem-derived lactone product.

Position	^{13}C (ppm)[a]	^{1}H (ppm)
1	45.65	2.53
2	60.04	4.00
3		
4		
5	70.68	4.58
6	55.31	4.08
7	175.58	
8	75.96	4.99
9	17.94	1.58
10		
11	16.45	1.02
12, 14	56.39	4.54, 5.06
13		
15, 16	146.13	8.96

[a]Representative spectra are shown in Figures S47-S50.

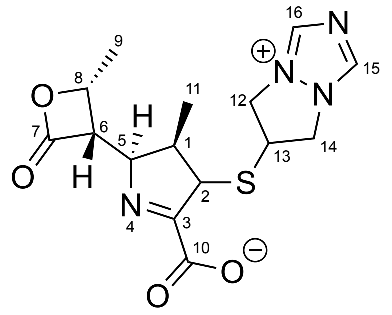

Note that while the stereochemistry at C-2 was not investigated by NOESY, this is likely the (2*S*)-diastereomer, based on a comparison of chemical shifts and coupling constants (2H: J = 3.9 Hz, 1.4 Hz) with the chemical shifts (Table S4) and coupling constants (2H: J = 3.1 Hz, 1.4 Hz) of the ertapenem-derived (2*S*)-lactone product.

Table S10. Chemical shift assignments for the major biapenem-derived hydrolysis product.

Position	^{13}C (ppm)[a]	^{1}H (ppm)
1	46.72	2.48
2	60.11	3.93
3		
4		
5	75.64	4.30
6	58.91	2.54
7	182.98	
8	70.81	3.97
9	23.43	1.19
10		
11	16.37	1.03
12, 14	56.39	4.54, 5.06
13		
15, 16	146.13	8.95

[a]Representative spectra are shown in Figures S47-S50.

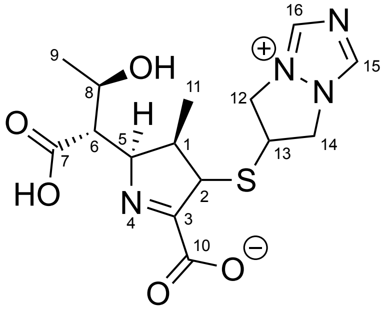

Note that while the stereochemistry at C-2 was not investigated by NOESY, this is likely the (2*S*)-diastereomer, based on a comparison of chemical shifts and coupling constants (2H: J = 3.8 Hz, 1.3 Hz) with the chemical shifts (Table S2) and coupling constants (2H: J = 3.5 Hz, 1.3 Hz) of the ertapenem-derived (2*S*)-hydrolysis product.

Table S11. Chemical shift assignments for doripenem.

Position	^{13}C (ppm)[a]	^{1}H (ppm)
1	45.13	3.31
2	142.03	
3	135.11	
4		
5	58.46	4.16
6	61.24	3.39
7	179.12	
8	67.72	4.18
9	22.72	1.22
10		
11	18.45	1.15
12	55.09	3.27, 3.56
13	42.21	3.93
14	35.65	1.63, 2.62
15	61.87	3.77
16	46.24	3.33, 3.41

[a]Spectra are shown in Figures S51-S54.

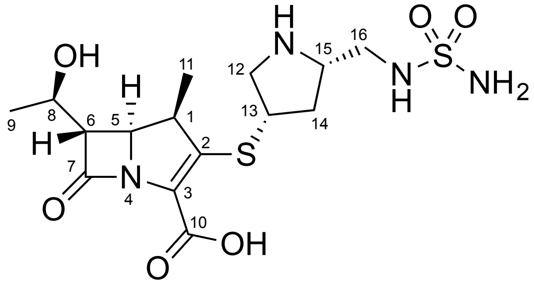

Table S12. Chemical shift assignments for the major doripenem-derived lactone product.

Position	^{13}C (ppm)[a]	^{1}H (ppm)
1	45.67	2.52
2	59.78	3.86
3		
4		
5	70.59	4.56
6	55.40	4.04
7	175.53	
8	75.99	4.99
9	17.86	1.57
10		
11	16.61	0.97
12	54.20	3.22, 3.54
13	43.32	3.66
14	37.94	1.63, 2.55
15	61.85	3.75
16	46.43	3.30, 3.38

[a]Representative spectra are shown in Figures S55-S58.

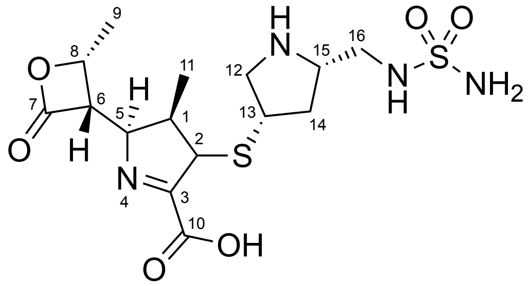

Note that while the stereochemistry at C-2 was not investigated by NOESY, this is likely the (2*S*)-diastereomer, based on a comparison of chemical shifts and coupling constants (2H: 3.3 Hz, 1.4 Hz) with the chemical shifts (Table S4) and coupling constants (2H: J = 3.1 Hz, 1.4 Hz) of the ertapenem-derived (2*S*)-lactone product.

Table S13. Chemical shift assignments for the major doripenem-derived hydrolysis product.

Position	^{13}C (ppm)[a]	^{1}H (ppm)
1	46.81	2.48
2	59.98	3.81
3	175.29	
4		
5	75.93	4.26
6	58.76	2.54
7	182.94	
8	71.06	3.97
9	23.41	1.19
10		
11	16.69	0.99
12	54.26	3.25, 3.66
13	43.24	3.67
14	37.87	1.65, 2.57
15	62.13	3.78
16	46.28	3.32, 3.41

[a]Representative spectra are shown in Figures S55-S58.

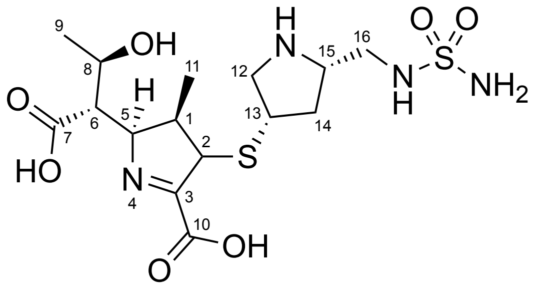

Note that while the stereochemistry at C-2 was not investigated by NOESY, this is likely the (2*S*)-diastereomer, based on a comparison of chemical shifts and coupling constants (2H: J = 3.0 Hz, 1.1 Hz) with the chemical shifts (Table S2) and coupling constants (2H: J = 3.5 Hz, 1.3 Hz) of the ertapenem-derived (2*S*)-hydrolysis product.

Table S14. Chemical shift assignments for meropenem.

Position	^{13}C (ppm)[a]	^{1}H (ppm)
1	45.30	3.34
2	142.12	
3	134.80	
4		
5	58.74	4.16
6	61.34	3.39
7	179.13	
8	67.96	4.19
9	22.92	1.23
10		
11	18.59	1.15
12	55.67	3.25, 3.46
13	44.32	3.90
14	37.01	1.73, 2.88
15	61.19	4.48
16	172.81	
17, 18	38.40, 39.31	2.93, 3.01

[a]Spectra are shown in Figures S59-S62.

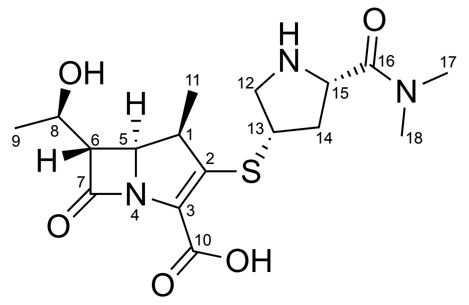

Table S15. Chemical shift assignments for the major meropenem-derived lactone product.

Position	^{13}C (ppm)[a]	^{1}H (ppm)
1	45.75	2.49
2	59.79	3.85
3	176.65	
4		
5	70.77	4.55
6	55.38	4.04
7	175.57	
8	76.56	4.98
9	18.08	1.56
10		
11	16.70	0.96
12	54.57	3.26, 3.56
13	44.71	3.66
14	38.80	1.77, 2.85
15	60.37	4.52
16	172.26	
17, 18	38.75, 39.48	2.91, 3.00

[a]Representative spectra are shown in Figures S63-S66.

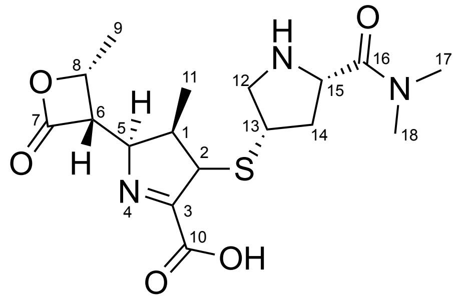

Note that while the stereochemistry at C-2 was not investigated by NOESY, this is likely the (2*S*)-diastereomer, based on a comparison of chemical shifts and coupling constants (2H: *J* = 3.2 Hz, 1.4 Hz) with the chemical shifts (Table S4) and coupling constants (2H: *J* = 3.1 Hz, 1.4 Hz) of the ertapenem-derived (2*S*)-lactone product.

Table S16. Chemical shift assignments for the major meropenem-derived hydrolysis product.

Position	^{13}C (ppm)[a]	^{1}H (ppm)
1	46.90	2.46
2	59.96	3.80
3	175.03	
4		
5	75.76	4.23
6	58.84	2.53
7	182.92	
8	70.95	3.96
9	23.39	1.19
10		
11	16.68	0.98
12	54.78	3.27, 3.61
13	44.67	3.67
14	38.80	1.78, 2.86
15	60.40	4.57
16	172.04	
17, 18	38.71, 39.47	2.91, 3.00

[a]Representative spectra are shown in Figures S63-S66.

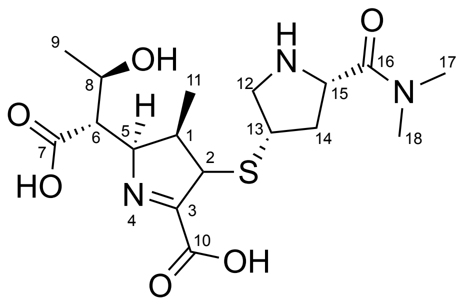

Note that while the stereochemistry at C-2 was not investigated by NOESY, this is likely the (2*S*)-diastereomer, based on a comparison of chemical shifts and coupling constants (2H: *J* = 3.1 Hz, 1.3 Hz) with the chemical shifts (Table S2) and coupling constants (2H: *J* = 3.5 Hz, 1.3 Hz) of the ertapenem-derived (2*S*)-hydrolysis product.

Table S17. Chemical shift assignments for imipenem.

Position	^{13}C (ppm)	^{1}H (ppm)
1	41.48	3.07, 3.19
2	141.01	
3	132.42	
4		
5	55.00	4.15
6	67.68	3.35
7	182.51	
8	67.70	4.17
9	22.77	1.22
10		
11	31.89<br>(34.12)[b]	2.97, 3.11<br>(2.90, 3.05)
12	43.75<br>(49.58)	3.51<br>(3.56)
13	157.33<br>(160.35)	7.76<br>(7.74)

[a]Spectra are shown in Figures S67-S70.
[b]Two sets of signals were observed for the N-formimidoyl sidechain.

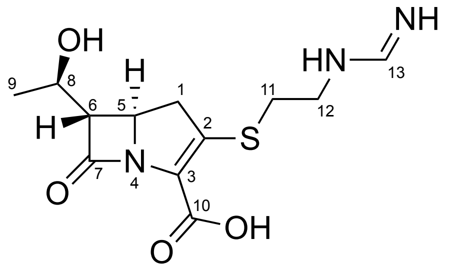

Table S18. Chemical shift assignments for the major imipenem-derived hydrolysis product.

Position	^{13}C (ppm)[a]	^{1}H (ppm)
1	37.40	2.69, 1.68
2	52.68	4.13
3	176.09	
4		
5	72.80	4.24
6	64.49	2.34
7	182.47	
8	70.47	4.12
9	24.11	1.19
10		
11	30.65 (32.92)[b]	2.79 (2.74)
12	43.81 (49.37)	3.47 (3.51)
13	157.30 (160.63)	7.73 (7.81)

[a]Representative spectra are shown in Figures S71-S74.
[b]Two sets of signals were observed for the N-formimidoyl sidechain.

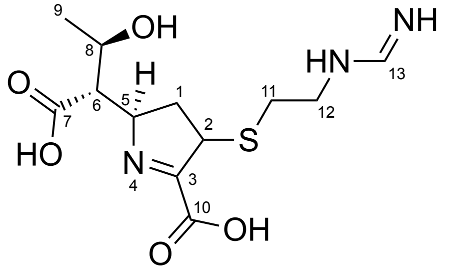

Note that the stereocenter at C-2 is expected to have an (*S*)-configuration based on a comparison of chemical shift assignments to those shown in previous studies of the imipenem-derived hydrolysis products.[18]

Table S19. Chemical shift assignments for panipenem.

Position	^{13}C (ppm)[a]	^{1}H (ppm)
1	42.36	3.26, 3.19
2	139.36	
3	134.30	
4		
5	67.91	4.23
6	68.02	3.44
7	182.35	
8		4.24
9	23.02	1.29
10		
11	56.64 (58.57)[b]	3.91, 3.47 (4.06, 3.64)
12	44.61 (44.98)	4.04 (4.01)
13	34.25 (33.92)	2.46, 2.09 (2.50, 2.13)
14	51.23 (49.94)	3.86, 3.75 (3.67, 3.58)
15	165.97	
16	21.22	2.27

[a]Spectra are shown in Figures S75-S78.
[b]Two sets of peaks were observed for the pyrrolidine ring.

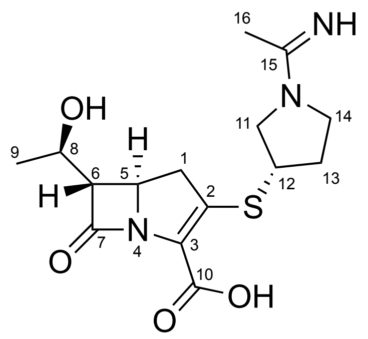

Table S20. Chemical shift assignments for the major panipenem-derived hydrolysis product.

Position	^{13}C (ppm)[a]	^{1}H (ppm)
1	38.66	1.75, 2.81
2	52.42	4.24
3		
4		
5	73.15	4.30
6	64.67	2.41
7		
8	70.55	4.18
9	24.12	1.26
10		
11	56.49	3.34, 3.88
12	44.25	3.67
13	34.70 (34.42)[b]	2.04, 2.43 (2.09, 2.47)
14	51.46 (49.56)	3.68, 3.81 (3.52, 3.62)
15	165.67	
16	21.15	2.27

[a]Representative spectra are shown in Figures S79-S82.
[b]Two sets of peaks were observed for the pyrrolidine ring.

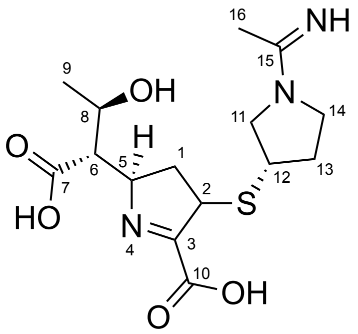

Note that the stereocenter at C-2 is expected to have an (*S*)-configuration based on a comparison of chemical shift assignments to those shown in previous studies of the imipenem-derived hydrolysis products.[18]

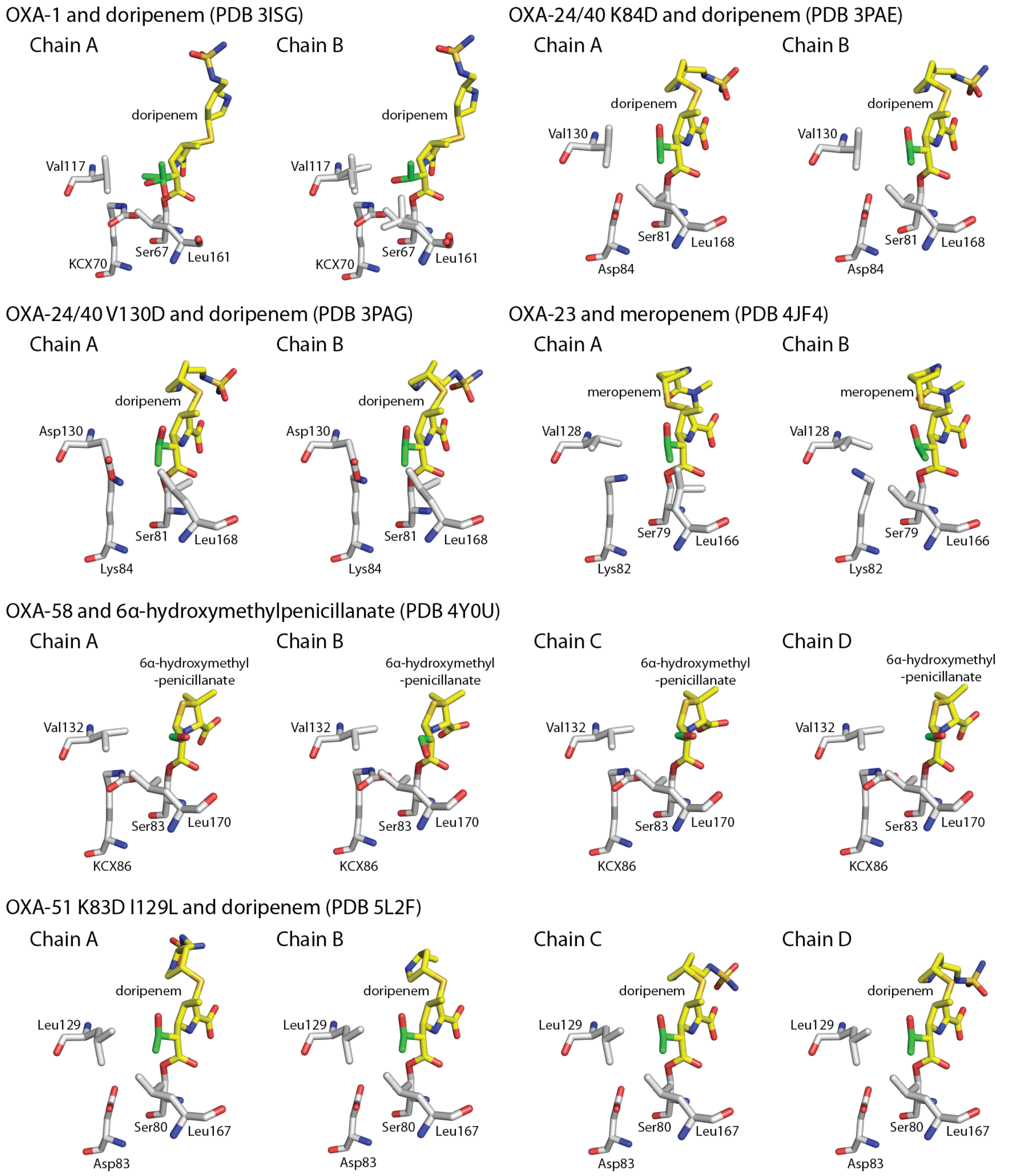

Figure S27. Views from crystal structures of OXA enzyme complexes derived by reaction with carbapenems. Views from the active sites of crystal structures of OXA-1 with doripenem,[5] OXA-24/40 K84D with doripenem,[19] OXA-24/40 V130D with doripenem,[19] OXA-23 with meropenem,[20] OXA-58 with 6α-hydroxymethylpenicillanate,[21] and OXA-51 K83D, I129L with doripenem.[22] Note the orientation of the hydroxyl group of the bound carbapenem/penicillanate (yellow sticks) relative to the ester carbonyl of the covalent link with the nucleophilic serine residue (white sticks). The carbons of the hydroxyethyl (or hydroxymethyl) side chain of the

carbapenem (or penicillinate) complex are shown in green. Note also the position of the carbamylated lysine residue (KCX) relative to the carbapenem/penicillanate hydroxyl group in the OXA-1/doripenem and OXA-58/6α-hydroxymethylpenicillanate structures. As can be seen in these structures, the 1β-methyl substituent of the carbapenems is oriented so as to interact sterically with the hydroxyethyl side chain.

Table S21. Cartesian coordinates and charges calculated for the doripenem (1β-Me) acyl-enzyme complex using the RESP method HF/6-31G*, as used for subsequent MD simulations.

doripenem Atom	X	Y	Z	RESP Charge
N	-6.311	2.552	0.439	-1.181961
C	-5.718	2.539	-0.884	0.482560
C	-5.263	1.174	-1.392	-0.147977
O	-4.333	0.627	-0.462	-0.310855
C	-6.683	3.182	-1.860	0.416651
O	-6.875	2.787	-2.964	-0.507074
C	-3.232	-0.432	3.903	-0.361217
C	-1.278	-2.661	2.040	-0.418167
N	7.962	2.414	0.016	-0.877754
O	-5.790	-0.838	0.346	-0.615833
O	-1.737	-2.331	-2.970	-0.601334
O	5.904	3.479	1.079	-0.578483
O	5.849	2.490	-1.186	-0.578483
O	0.425	-2.062	-2.690	-0.508815
O	-3.625	1.387	2.361	-0.725566
C	-4.704	-0.351	0.338	0.745821
C	3.766	-2.711	-0.900	0.275807
C	6.075	-0.221	0.214	-0.053808
C	3.541	-0.667	0.290	-0.243603
N	-2.167	-1.207	-0.596	-0.598822
N	4.968	-1.917	-1.148	-1.094118
N	6.030	1.063	0.915	-0.694320
S	1.366	-2.493	0.470	-0.399382
C	-0.675	-2.012	-2.258	0.699478
C	-0.988	-1.536	-0.861	0.313000
C	-3.928	0.036	2.631	0.309795
C	-0.998	-1.268	1.463	0.139133
C	4.812	-0.576	-0.570	0.668567
C	2.703	-1.695	-0.470	0.045052
C	-3.630	-0.769	1.342	-0.216047
C	-2.218	-0.624	0.754	0.317648
C	-0.002	-1.309	0.272	-0.149510
S	6.327	2.469	0.160	1.216147
H	-5.607	2.399	1.133	0.439132
H	-6.993	1.823	0.537	0.439132
H	-4.852	3.197	-0.844	0.005872
H	-4.737	1.266	-2.329	0.120353
H	-6.095	0.501	-1.518	0.120353
H	-7.201	4.061	-1.468	0.027242
H	-3.573	0.175	4.738	0.095595
H	-3.485	-1.460	4.133	0.095595
H	-2.156	-0.335	3.844	0.095595
H	-1.635	-3.348	1.279	0.137016
H	-2.022	-2.621	2.822	0.137016
H	-0.381	-3.082	2.474	0.137016
H	8.430	2.619	0.877	0.413426
H	8.273	2.997	-0.736	0.413426
H	-3.740	1.903	3.148	0.463042
H	3.465	-3.266	-1.779	0.063108
H	3.951	-3.428	-0.105	0.063108
H	6.931	-0.207	-0.446	0.110060
H	6.261	-0.985	0.958	0.110060
H	3.047	0.292	0.403	0.089277
H	3.771	-1.048	1.282	0.089277
H	5.173	-1.865	-2.123	0.430343
H	5.326	1.181	1.614	0.359158
H	-4.997	-0.065	2.796	0.037180
H	-0.609	-0.625	2.241	0.030167
H	4.665	0.168	-1.345	-0.051651
H	2.267	-1.253	-1.354	0.065968
H	-3.849	-1.808	1.553	0.101939
H	-2.028	0.435	0.633	0.019990
H	-2.530	-2.135	-2.477	0.434939
H	0.435	-0.324	0.144	0.140736

Table S22. Cartesian coordinates and charges calculated for the doripenem (1β-H) acyl-enzyme complex using the RESP method HF/6-31G*, as used for subsequent MD simulations.

doripenem-protonated				RESP
Atom	X	Y	Z	Charge
N	-7.010	-2.001	-0.033	-1.192228
C	-6.554	-1.660	1.299	0.490890
C	-5.857	-0.309	1.438	-0.148876
O	-4.737	-0.277	0.557	-0.339410
C	-7.726	-1.759	2.257	0.400353
O	-7.923	-0.997	3.148	-0.503252
C	-3.008	-0.861	-3.742	-0.245295
N	8.447	-1.767	0.594	-0.879666
O	-5.768	1.017	-0.922	-0.609450
O	-1.595	3.009	2.093	-0.588902
O	6.475	-3.379	0.512	-0.572946
O	6.479	-1.383	1.970	-0.572946
O	0.543	2.536	1.912	-0.507325
O	-4.045	-2.035	-1.893	-0.728838
C	-4.814	0.397	-0.572	0.736755
C	3.670	2.768	-0.559	0.271010
C	6.196	0.246	-0.602	-0.085806
C	3.627	0.393	-0.537	-0.238950
N	-2.155	1.067	0.356	-0.689319
N	4.982	2.305	-0.112	-1.098078
N	6.273	-1.215	-0.562	-0.688926
S	1.223	1.702	-1.309	-0.443388
C	-0.575	2.372	1.558	0.685769
C	-0.950	1.382	0.484	0.244535
C	-3.942	-0.783	-2.539	0.296353
C	-0.965	0.001	-1.402	-0.420699
C	4.964	0.848	0.072	0.664960
C	2.721	1.598	-0.281	0.046984
C	-3.578	0.257	-1.458	-0.300498
C	-2.305	-0.002	-0.640	0.773666
C	0.025	0.656	-0.419	0.128393
S	6.830	-2.013	0.737	1.198859
H	-6.230	-2.219	-0.622	0.444500
H	-7.499	-1.235	-0.456	0.444500
H	-5.857	-2.438	1.601	0.005778
H	-5.462	-0.179	2.433	0.127505
H	-6.524	0.507	1.213	0.127505
H	-8.390	-2.606	2.069	0.033471
H	-3.452	-1.501	-4.499	0.064850
H	-2.862	0.116	-4.190	0.064850
H	-2.044	-1.278	-3.483	0.064850
H	8.862	-2.346	-0.110	0.415825
H	8.901	-1.860	1.482	0.415825
H	-4.239	-2.709	-2.532	0.459948
H	3.368	3.670	-0.042	0.068463
H	3.697	2.994	-1.622	0.068463
H	7.096	0.636	-0.148	0.121237
H	6.214	0.539	-1.644	0.121237
H	3.259	-0.524	-0.091	0.084659
H	3.725	0.233	-1.608	0.084659
H	5.263	2.768	0.726	0.433571
H	5.536	-1.727	-1.000	0.361682
H	-4.919	-0.487	-2.907	0.041484
H	-0.642	-0.986	-1.701	0.145773
H	4.982	0.589	1.125	-0.041732
H	2.413	1.631	0.753	0.064640
H	-3.486	1.215	-1.960	0.105885
H	-2.430	-0.925	-0.091	-0.076386
H	-2.412	2.680	1.725	0.438982
H	0.570	-0.084	0.154	0.078473
H	-1.038	0.613	-2.293	0.145773

Table S23. Cartesian coordinates and charges calculated for the carbamylated lysine (KCX) using the RESP method HF/6-31G*, as used for subsequent MD simulations.

carbamyllysine<br>Atom	X	Y	Z	RESP<br>Charge
N	-2.078	-1.72	-0.342	-1.07936
C	-2.514	-0.438	0.191	0.365783
C	-1.595	0.701	-0.282	-0.406189
C	-0.17	0.595	0.267	0.261469
C	0.71	1.786	-0.109	-0.259857
C	2.184	1.567	0.255	0.465934
N	2.809	0.52	-0.515	-0.87078
C	-3.908	-0.181	-0.337	0.537326
O	-4.82	0.229	0.311	-0.568047
C	3.083	-0.766	0.057	1.0769
O	3.971	-1.395	-0.518	-0.858684
O	2.381	-1.078	1.026	-0.858684
H	-2.46	-2.476	0.193	0.393748
H	-1.082	-1.81	-0.297	0.393748
H	-2.567	-0.414	1.278	-0.028042
H	-2.042	1.643	0.029	0.096128
H	-1.572	0.704	-1.371	0.096128
H	-0.204	0.506	1.35	0.024758
H	0.323	-0.301	-0.08	0.024758
H	0.34	2.688	0.385	0.015124
H	0.645	1.965	-1.182	0.015124
H	2.26	1.343	1.314	-0.079913
H	2.716	2.503	0.081	-0.079913
H	-4.026	-0.394	-1.405	-0.025117
H	3.554	0.812	-1.106	0.347659

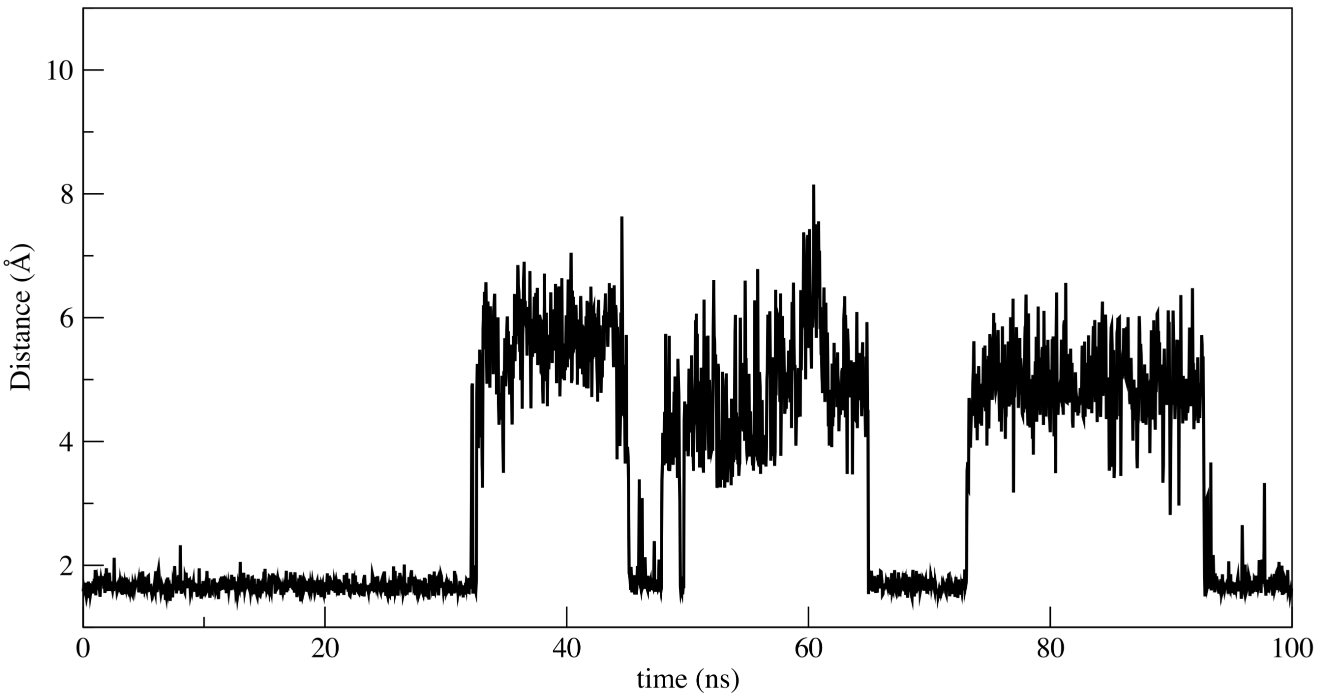

Figure S28. Plot showing the variations in distance between the OXA-1 carbamylated lysine and the hydroxyethyl side chain of the doripenem (1β-Me) acyl-enzyme complex during the MD simulation. The distance indicated is that between an oxygen of the carbamylated lysine and the hydroxyl proton of the doripenem hydroxyethyl side chain (see Figure 2C). Note that the conformation of the carbamylated lysine was also observed to change over the duration of the simulation.

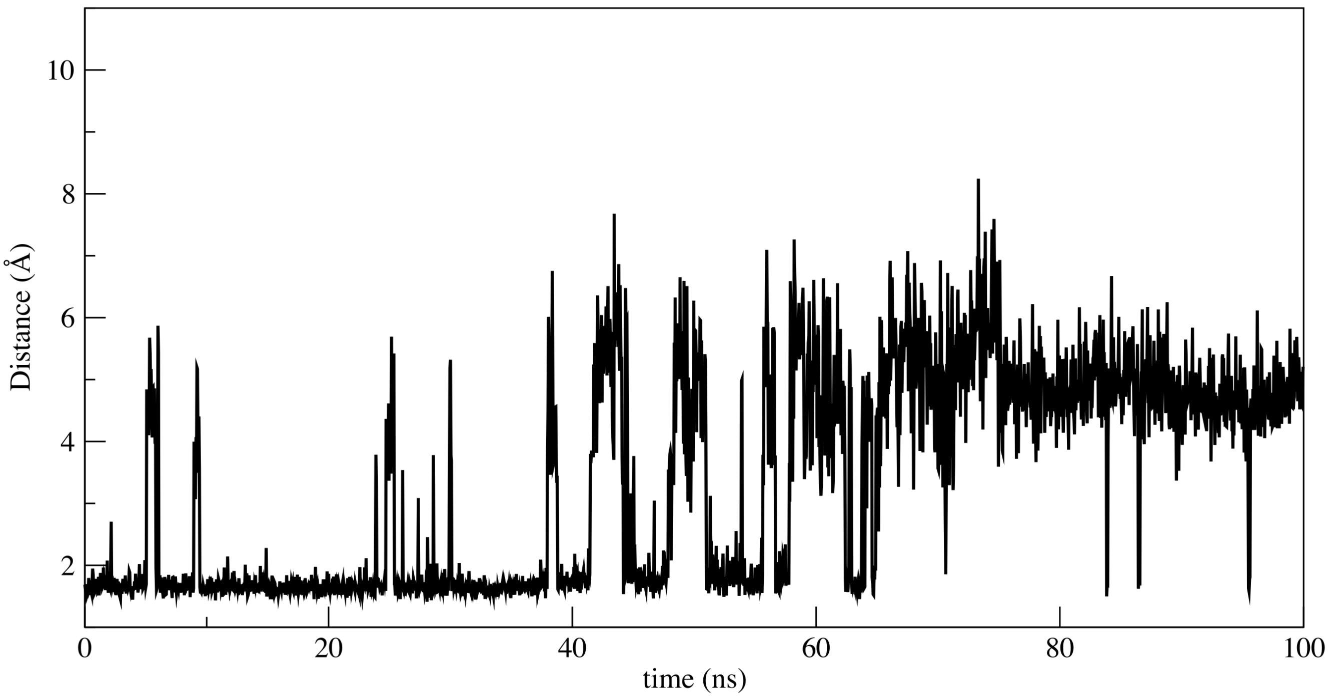

Figure S29. Plot showing the distance between the OXA-1 carbamylated lysine and the hydroxyethyl side chain of the doripenem (1β-H) acyl-enzyme complex over 100 ns of MDS. Note that the 1β-H system is highly dynamic (relative to that observed for the system with a 1β-methyl substituent; Figure S28) and that the hydroxyethyl sidechain samples a 'longer distance' secondary orientation early in the simulation. This position, in which the hydroxyethyl oxygen is further from the carbamylated lysine, appears to stabilize towards the end of the simulation. The distance indicated is that between an oxygen of the carbamylated lysine and the hydroxyl proton of the doripenem hydroxyethyl side chain (see Figure 2C).

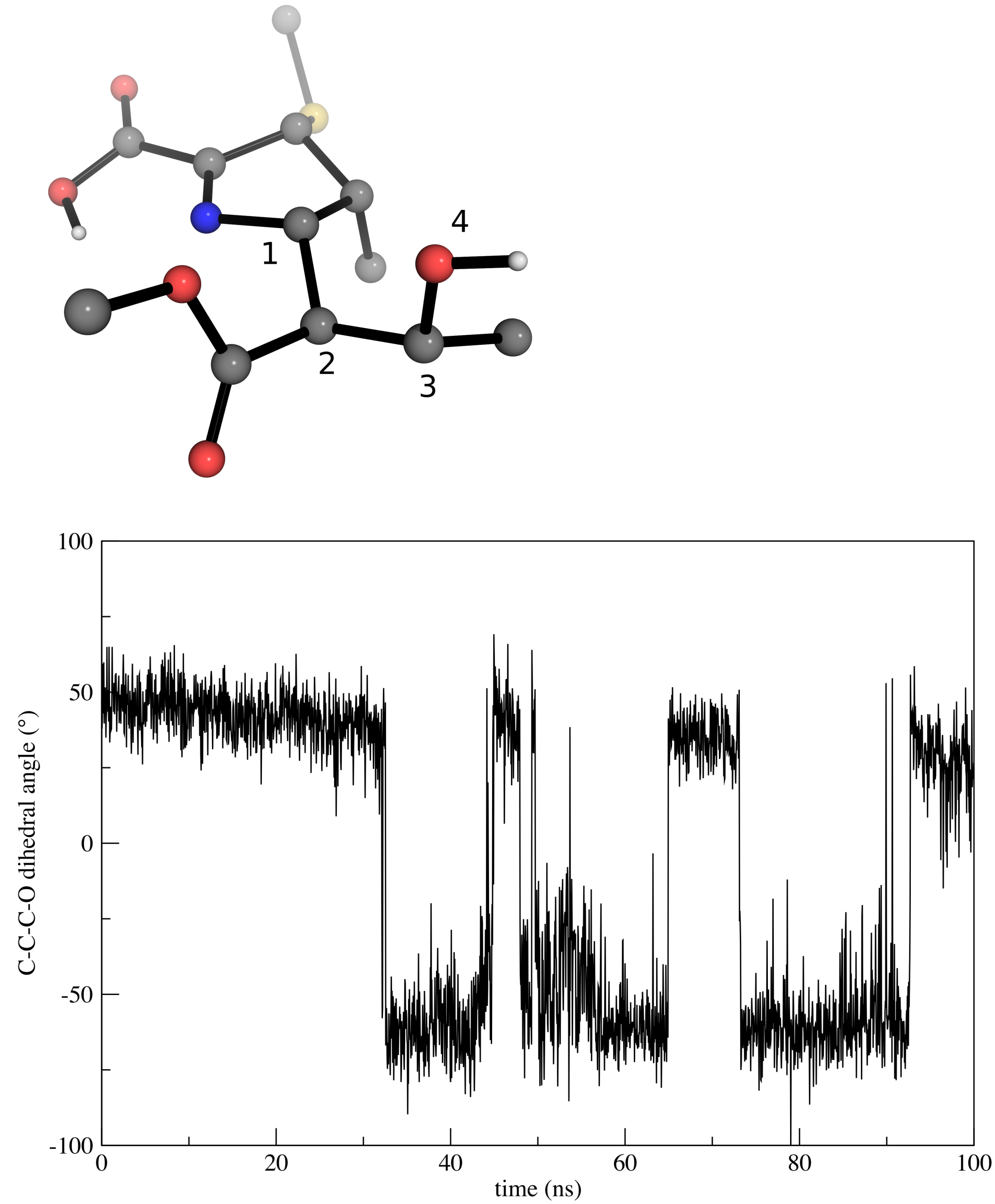

Figure S30. Dihedral angle of the doripenem (1β-Me) acyl-enzyme complex hydroxyethyl sidechain C-C-C-O (atoms numbered 1-4) in the above structure over 100 ns. The atoms on which the dihedral angle is based are indicated on the structure shown above the plot. The doripenem sulfide sidechain and the nucleophilic serine 'backbone' are both represented as methyl groups.

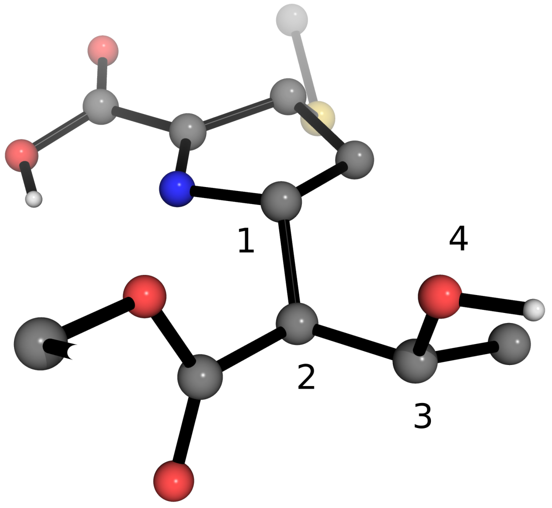

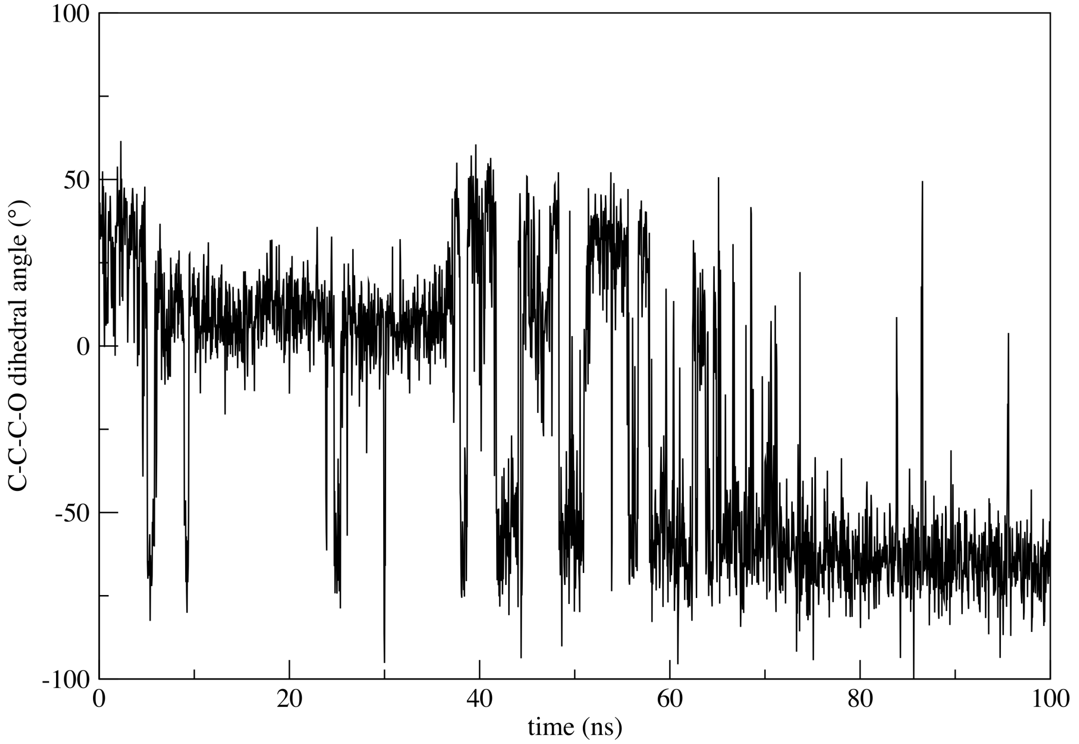

Figure S31. Dihedral angle of the doripenem (1β-H) acyl-enzyme complex hydroxyethyl sidechain C-C-C-O (atoms numbered 1-4) in the above structure over 100 ns. The atoms on which the dihedral angle is based are indicated on the structure shown above the plot. The doripenem sulfide sidechain and the nucleophilic serine 'backbone' are both represented as methyl groups.

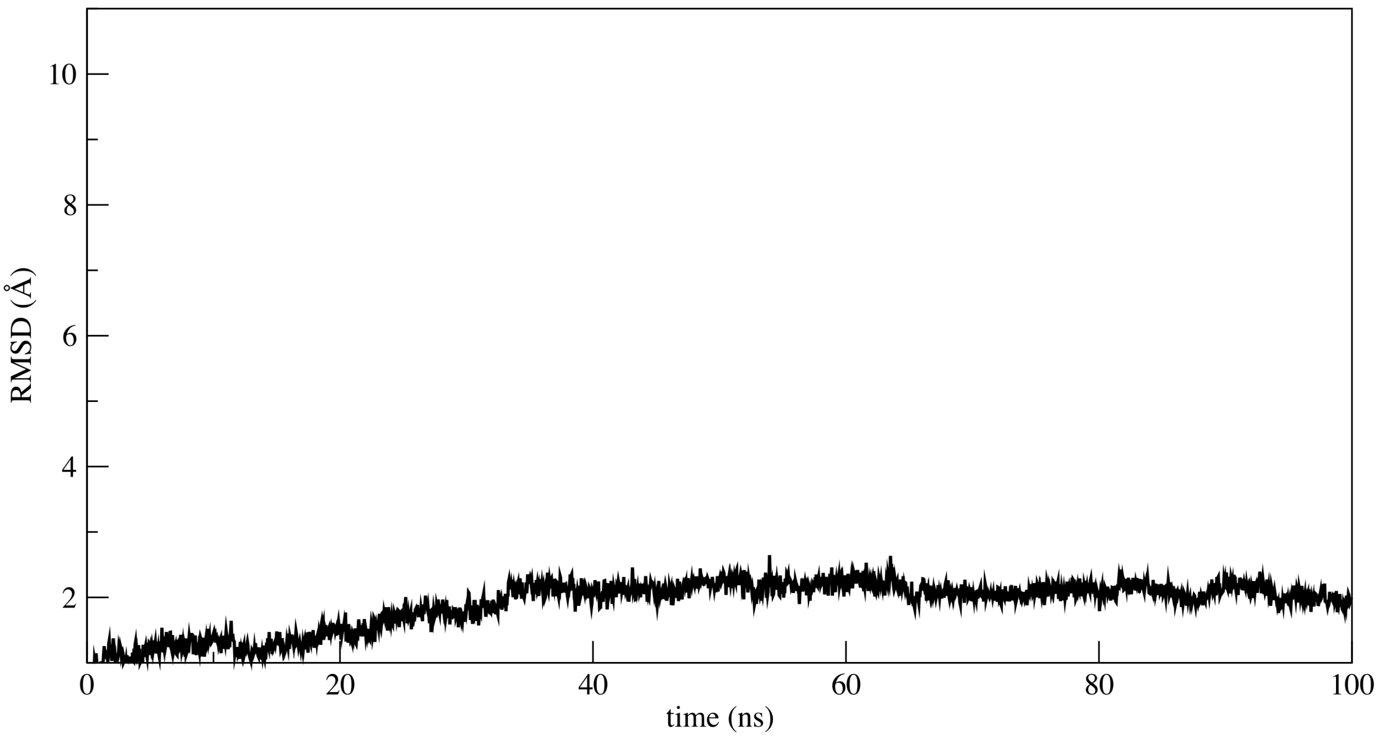

Figure S32. Plot of the root-mean-square-deviation of protein Cα atoms for the OXA-1:1β-Me doripenem acyl-enzyme complex over 100 ns of MD simulations (1.889 ± 0.38).

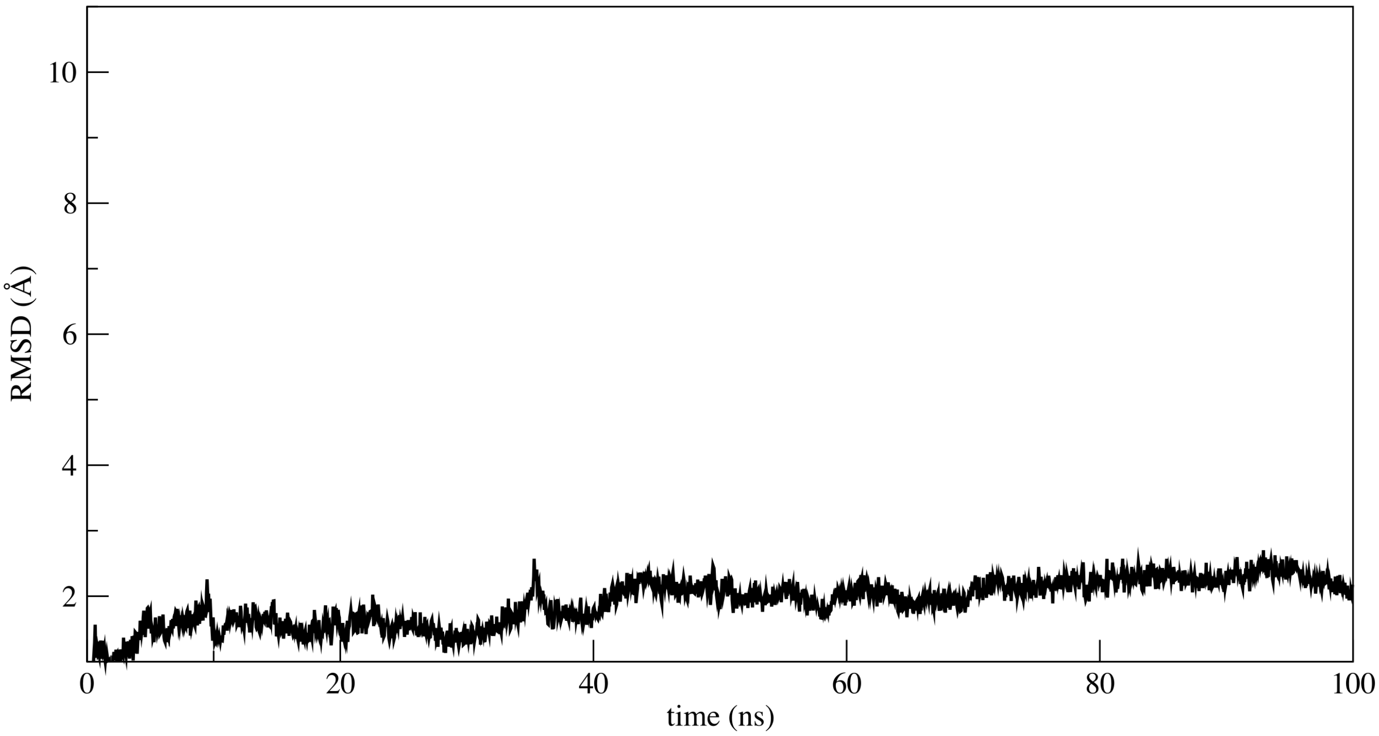

Figure S33. Plot of the root-mean-square-deviation of protein Cα atoms for the OXA-1:1β-H doripenem acyl-enzyme complex over 100 ns of MD simulations (1.897 ± 0.36).

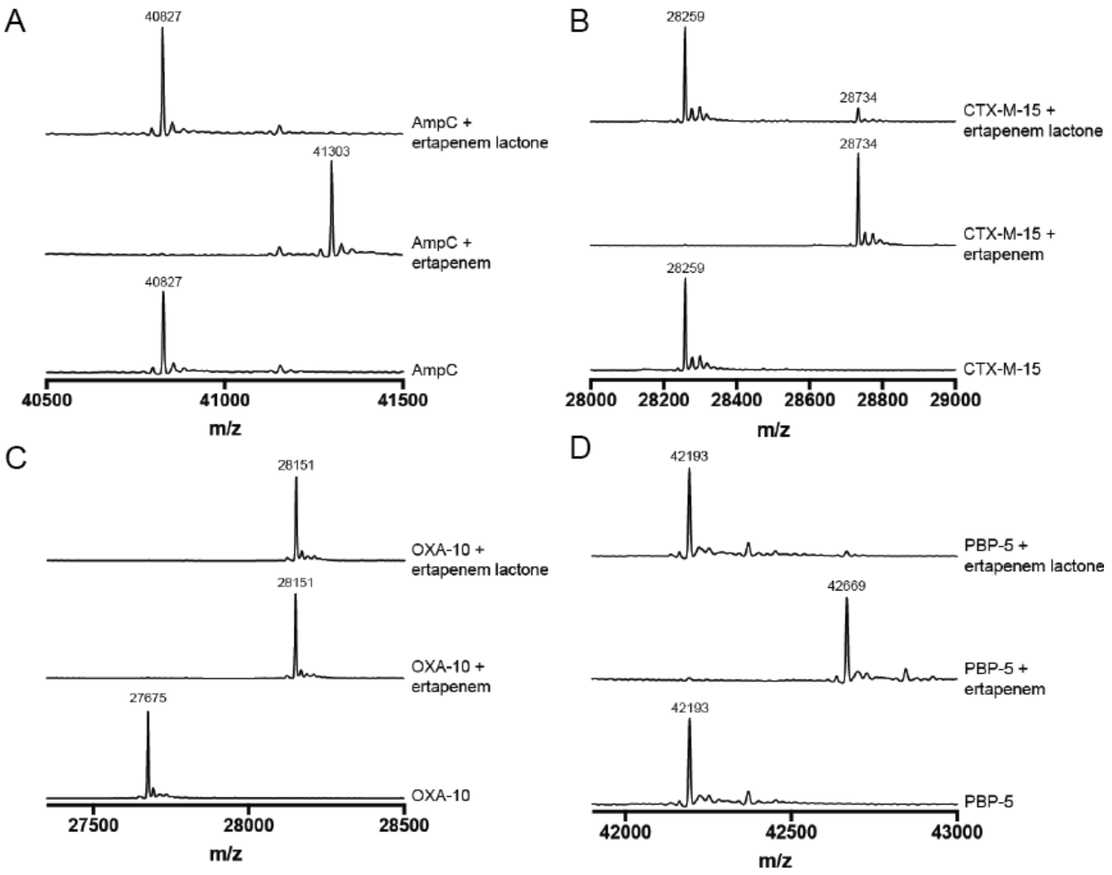

Figure S34. Extent of acylation of non-carbapenemase SBLs and PBPs by ertapenem lactone. The acylation of (A) AmpC, (B) CTX-M-15, (C) OXA-10, and (D) PBP-5 by ertapenem and ertapenem lactone was investigated by ESI-MS under denaturing conditions. A mixture of protein (1.2 μM) and 100 μM of ertapenem or ertapenem lactone was prepared in 50 mM sodium phosphate, pH 7.5, incubated for 30 min, and analyzed. Mass spectra were deconvoluted via MaxEnt 1 processing in MassLynx V4.0 (Waters). Note that we did not acquire mass spectra for the SBLs which catalyzed the hydrolysis of the carbapenem-derived β-lactones (e.g., OXA-23, OXA-48, SFC-1; Figure 3A); however, the observed hydrolysis implies that an acyl-enzyme complex is formed.

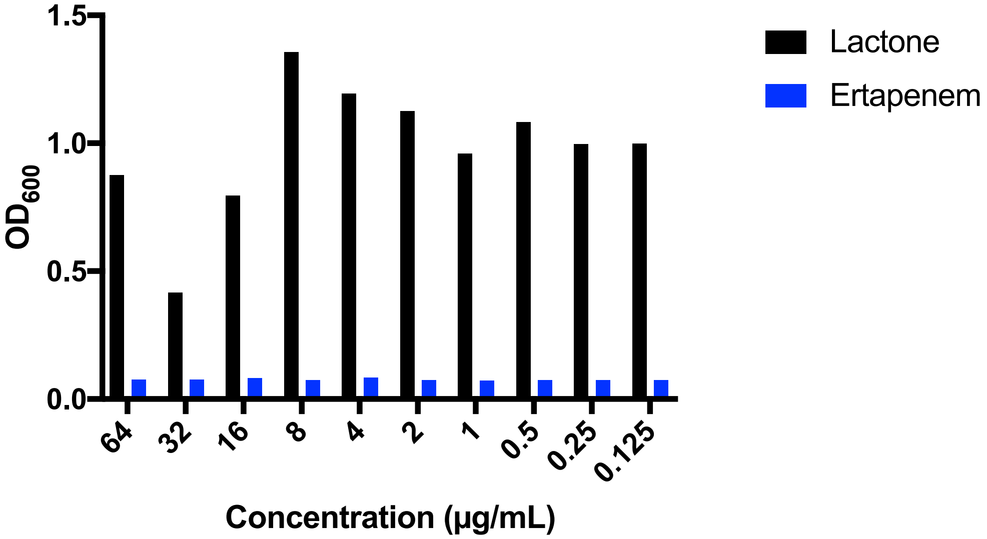

Figure S35. Antibiotic activity testing of the ertapenem-derived lactone. Serial dilutions of ertapenem and the ertapenem-derived lactone were tested for antibiotic activity against *Escherichia coli* BW25113. In 96-well plate format, a suspension of *E. coli* in saline (20 µL, OD_{600} = 0.01) was added to 180 µL calcium-supplemented Mueller-Hinton broth supplemented with the appropriate concentration of ertapenem or ertapenem-derived lactone. The plate was incubated at 37 °C overnight, and the optical density at 600 nm was measured using a PHERAstar FS microplate reader (BMG LabTech). While no *E. coli* growth was observed at any of the ertapenem concentrations tested, the concentrations of the ertapenem-derived lactone tested were insufficient to inhibit the visible growth of *E. coli*.

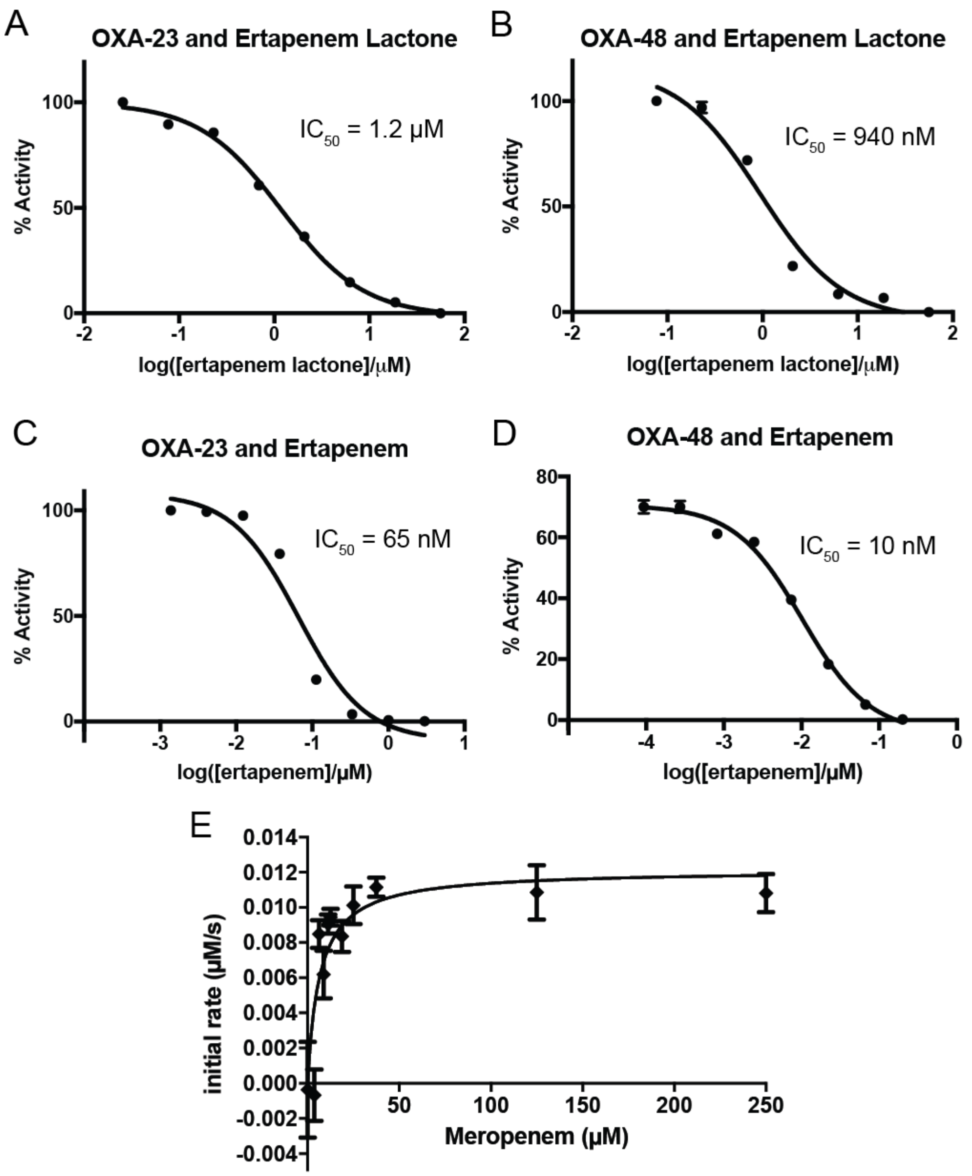

Figure S36. OXA enzyme inhibition by ertapenem and the ertapenem-derived lactone. The inhibitory activities of (A, B) the ertapenem-derived lactone and (C, D) ertapenem were tested against OXA-23 and OXA-48. The IC_{50} curves are based on eight serial dilutions tested in triplicate, and non-linear regression analysis was performed using Prism 7 (GraphPad). Assays were based on the hydrolysis of the fluorogenic substrate FC-5, as described in the Experimental section.[3] (E) Kinetic analyses were performed with OXA-48 and meropenem to test for potential substrate and/or product inhibition. Based on these data, these modes of inhibition did not appear to have a prominent impact on the kinetics of meropenem degradation by OXA-48. These assays were performed in Greiner UV-Star 96-well plates, employing 100 nM enzyme and the indicated concentrations of meropenem. Substrate degradation was monitored at 295 nm. These assays were otherwise performed as described above for the enzymatic FC-5 hydrolysis assays (in the Experimental section).

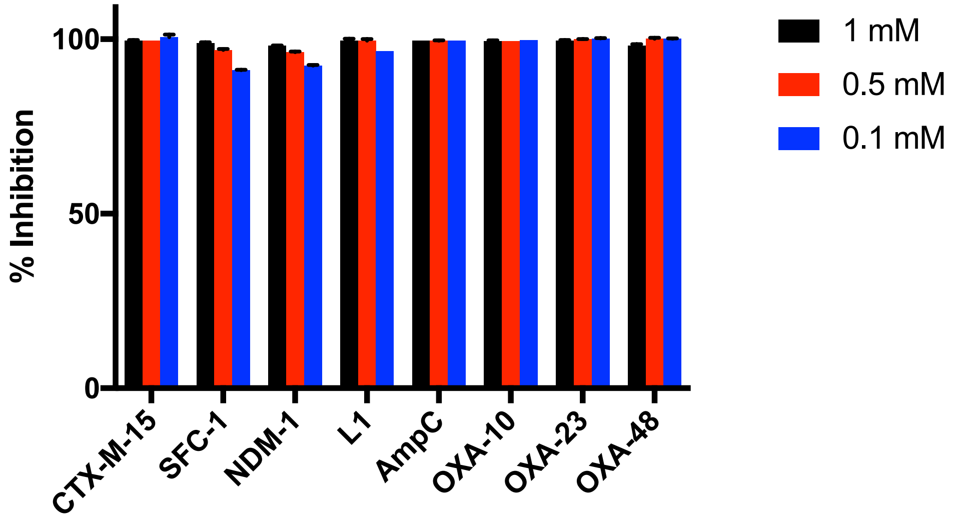

Figure S37. Impact of ertapenem on the activity of SBLs and MBLs. The hydrolysis of FC-5 (5 μM) by SBLs (CTX-M-15, SFC-1, AmpC, OXA-10, OXA-23, and OXA-48) and MBLs (NDM-1, L1) was tested in the presence of ertapenem (1 mM, 0.5 mM, or 0.1 mM). The assay conditions are described in more detail in the Experimental section.

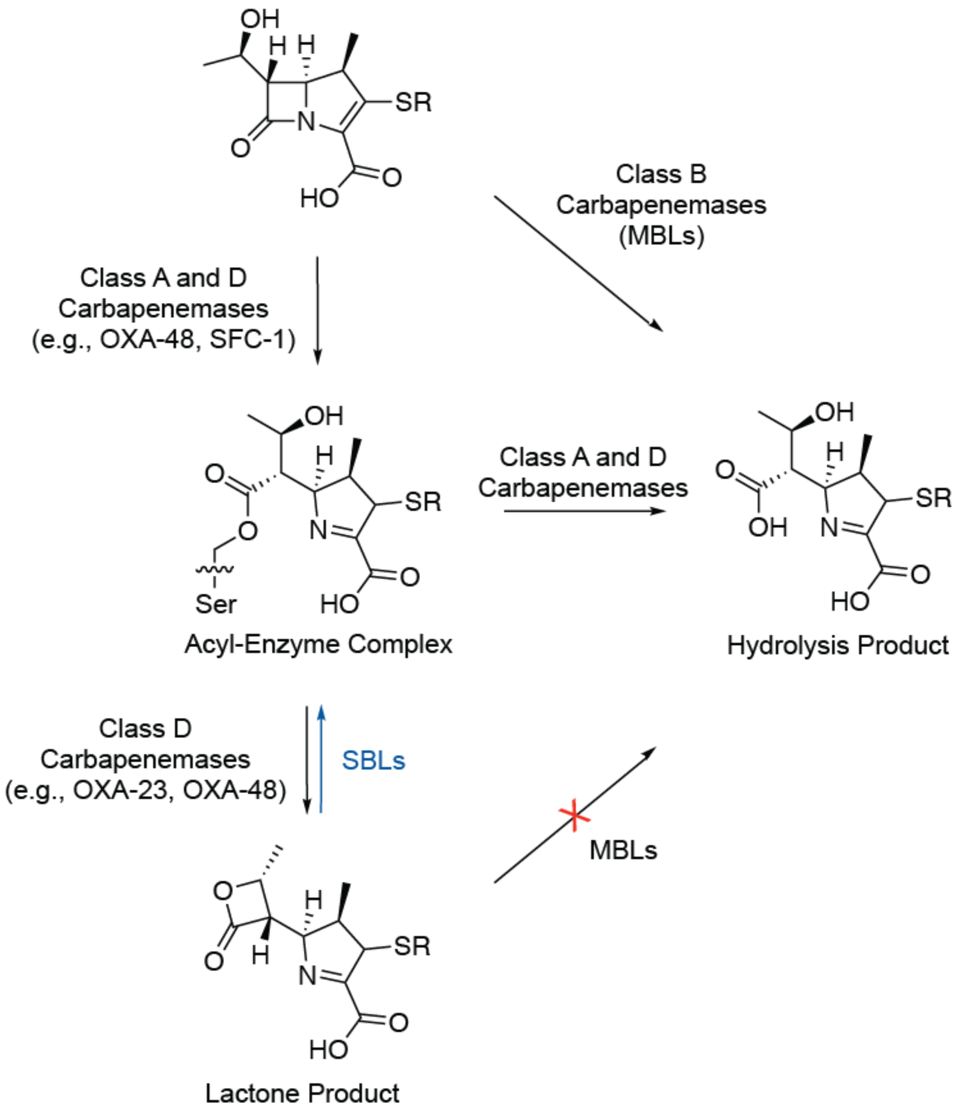

Figure S38. Interactions of carbapenems and carbapenem-derived lactones with β-lactamases. Carbapenems may be directly hydrolyzed by metallo-β-lactamases (MBLs; class B β-lactamases) with carbapenemase activity, and by serine β-lactamases (SBLs; shown here for class A and class D β-lactamases) with carbapenemase activity via an acyl-enzyme intermediate. We provide evidence that, in addition to the hydrolysis product, class D carbapenemases form a β-lactone product from carbapenems with a 1β-methyl group. These β-lactones both inhibit and re-acylate some SBLs (most likely re-forming the acyl-enzyme complex); while they inhibit MBLs, they are not observed to be hydrolyzed by MBLs under our assay conditions.

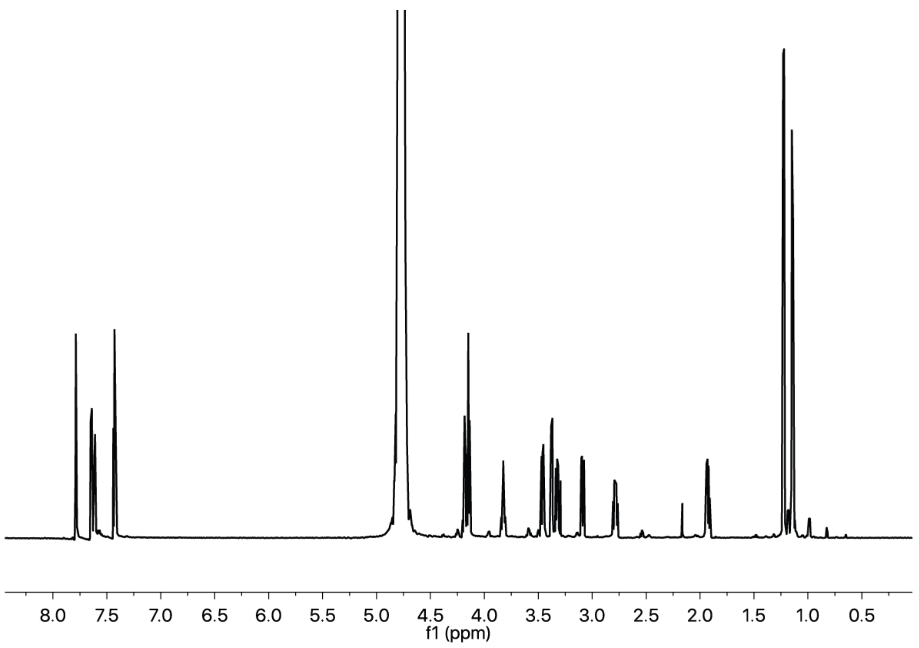

Figure S39. ^{1}H NMR spectrum of ertapenem. Sample consisted of 5 mM ertapenem in 50 mM sodium phosphate, pH 7.5, 10 % D_2O, and the spectrum was measured at a field strength of 700 MHz.

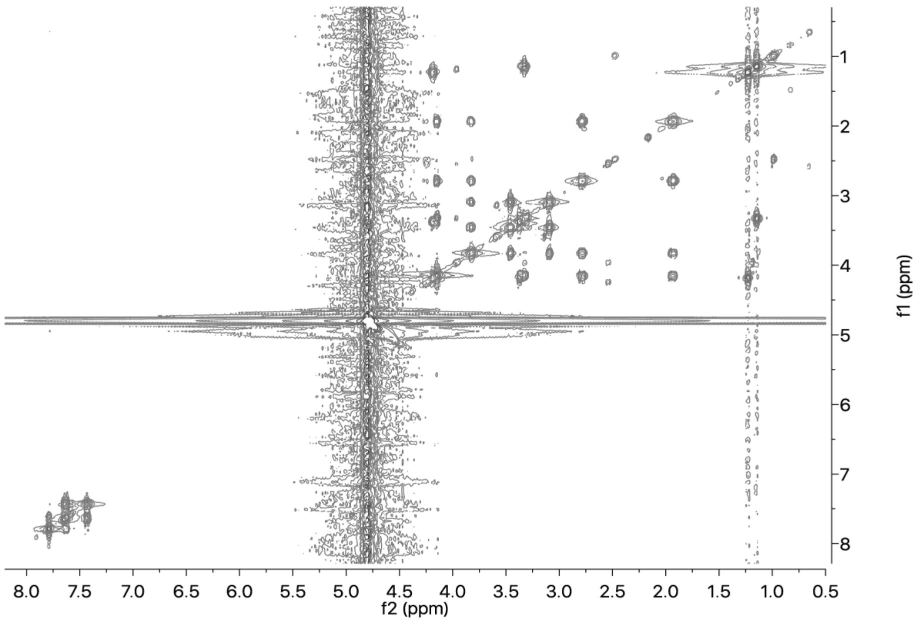

Figure S40. COSY spectrum of ertapenem. Sample consisted of 5 mM ertapenem in 50 mM sodium phosphate, pH 7.5, 10 % D_2O, and the spectrum was measured at a field strength of 700 MHz.

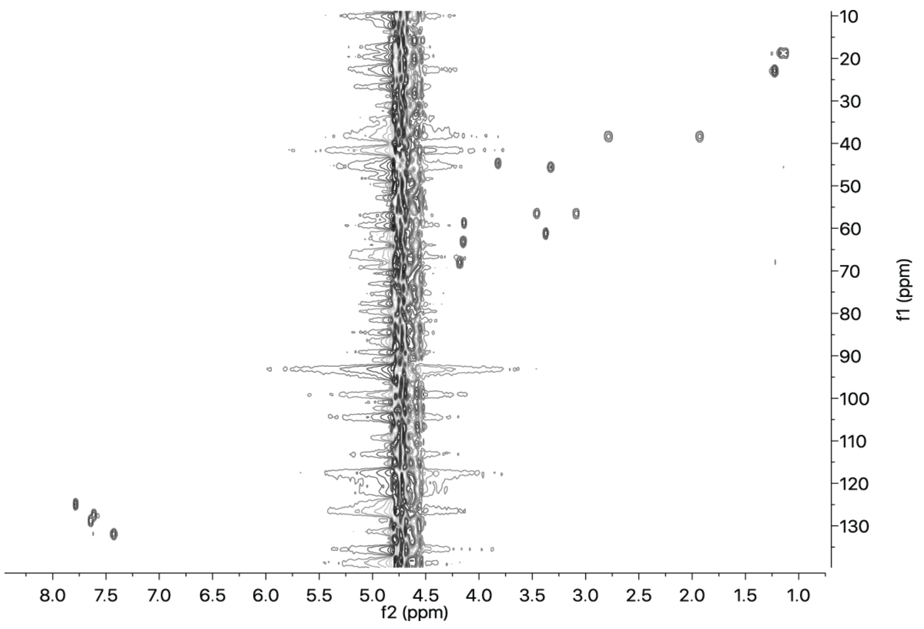

Figure S41. ^{1}H-^{13}C HSQC spectrum of ertapenem. Sample consisted of 5 mM ertapenem in 50 mM sodium phosphate, pH 7.5, 10 % D_2O, and the spectrum was measured at a field strength of 700 MHz.

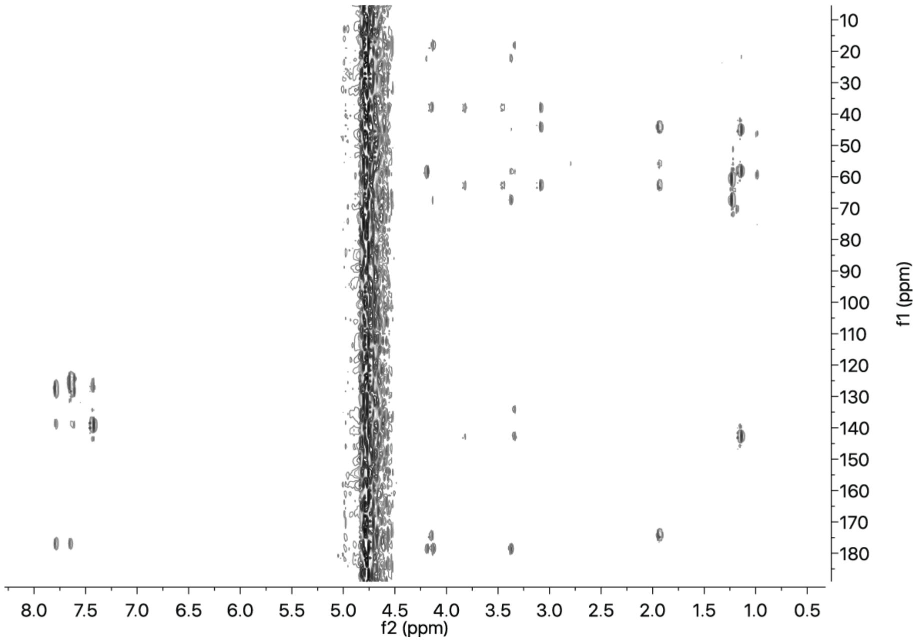

Figure S42. ^{1}H-^{13}C HMBC spectrum of ertapenem. Sample consisted of 5 mM ertapenem in 50 mM sodium phosphate, pH 7.5, 10 % D_2O, and the spectrum was measured at a field strength of 700 MHz.

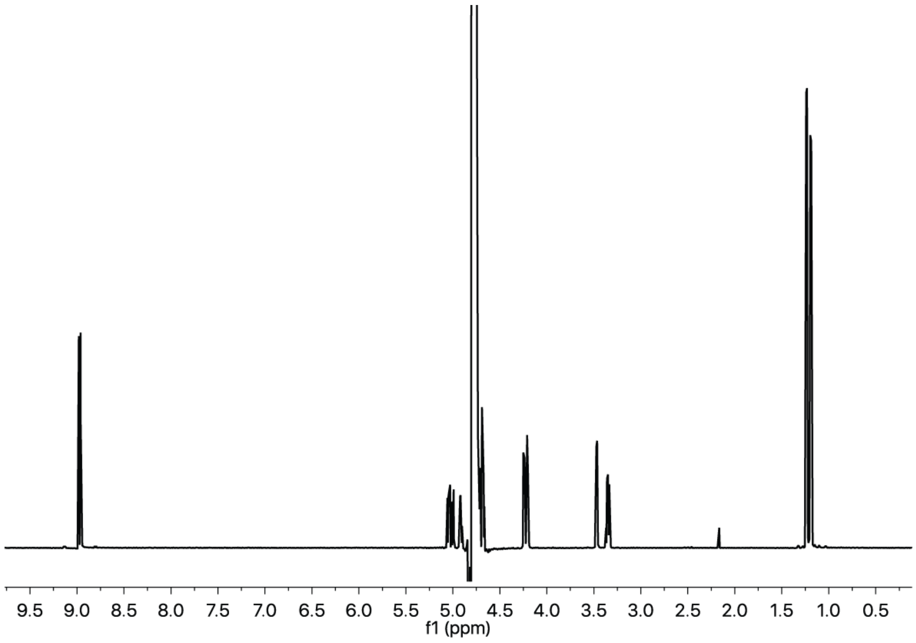

Figure S43. ¹H NMR spectrum of biapenem. Sample consisted of 5 mM biapenem in 50 mM sodium phosphate, pH 7.5, 10 % D_2O, and the spectrum was measured at a field strength of 700 MHz.

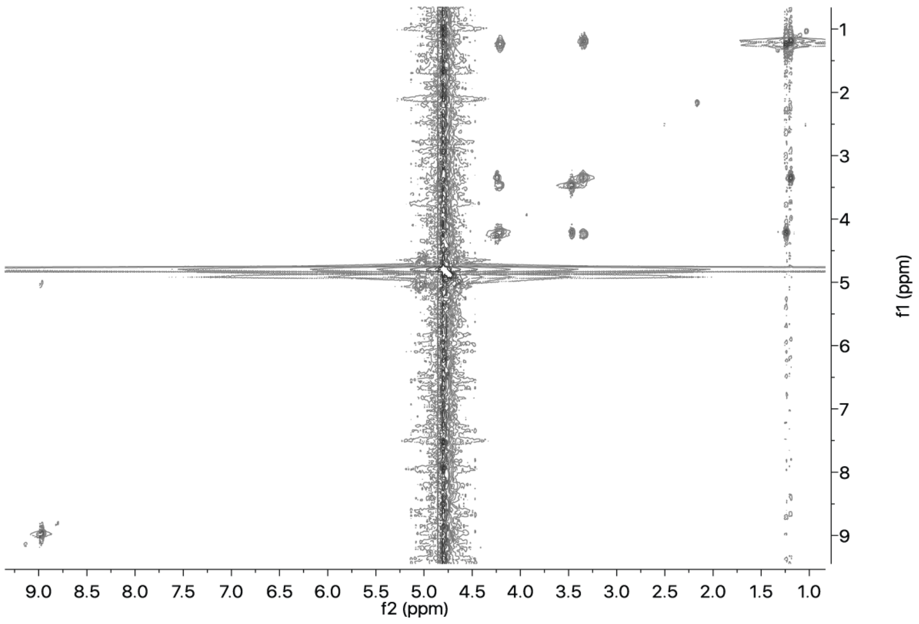

Figure S44. COSY spectrum of biapenem. Sample consisted of 5 mM biapenem in 50 mM sodium phosphate, pH 7.5, 10 % D_2O, and the spectrum was measured at a field strength of 700 MHz.

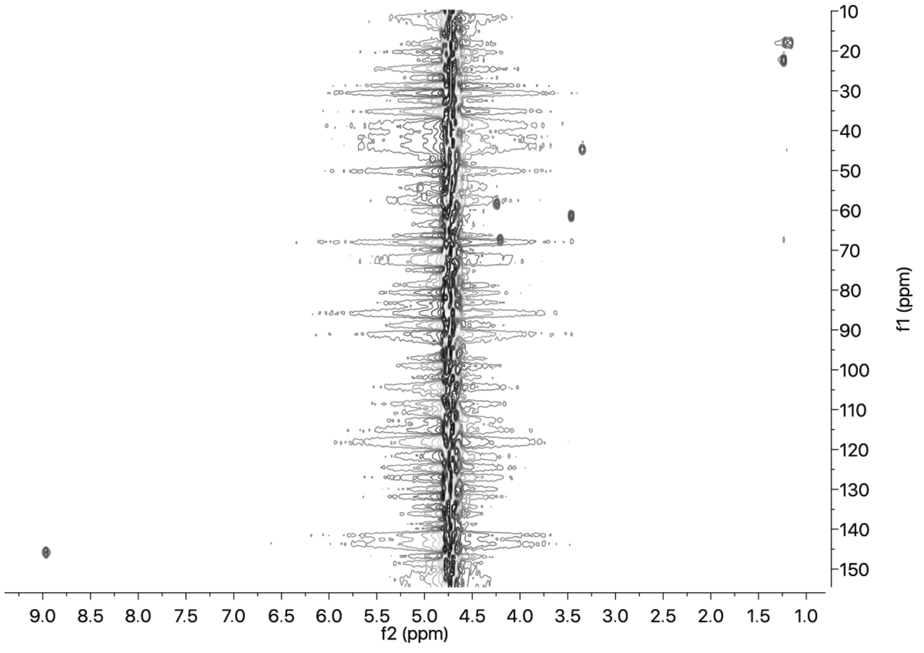

Figure S45. ^{1}H-^{13}C HSQC spectrum of biapenem. Sample consisted of 5 mM biapenem in 50 mM sodium phosphate, pH 7.5, 10 % D_2O, and the spectrum was measured at a field strength of 700 MHz.

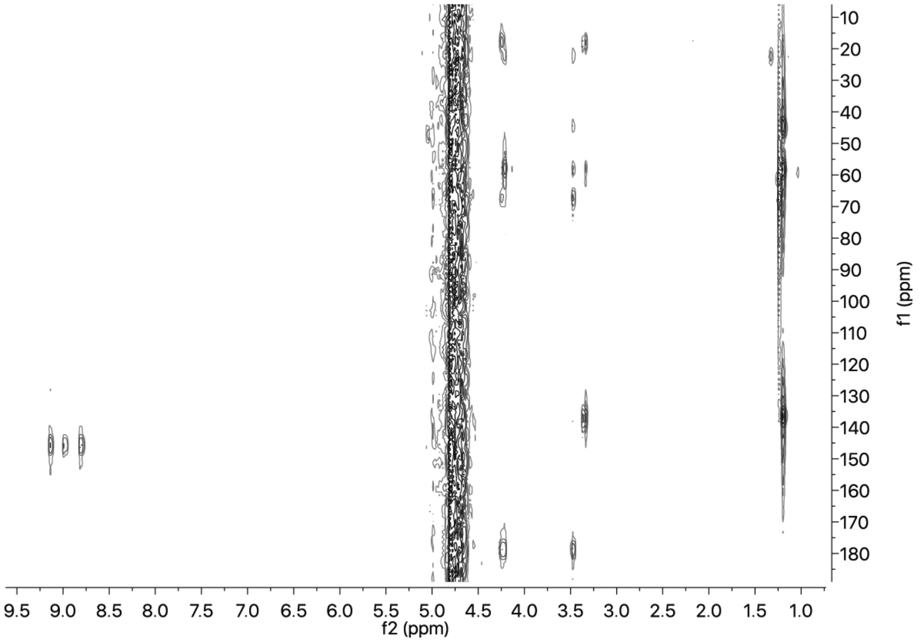

Figure S46. ^{1}H-^{13}C HMBC spectrum of biapenem. Sample consisted of 5 mM biapenem in 50 mM sodium phosphate, pH 7.5, 10 % D_2O, and the spectrum was measured at a field strength of 700 MHz.

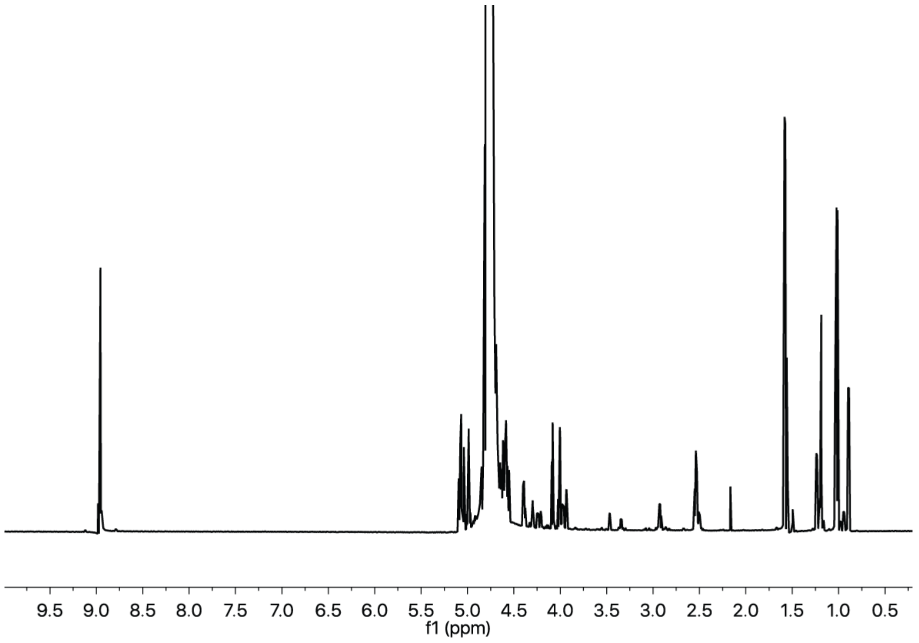

Figure S47. ^{1}H NMR spectrum of the mixture of biapenem and OXA-48. Sample consisted of 2 mM biapenem and 2.5 µM OXA-48 in 50 mM sodium phosphate, pH 7.5, 10 % D_2O, and the spectrum was measured at a field strength of 700 MHz.

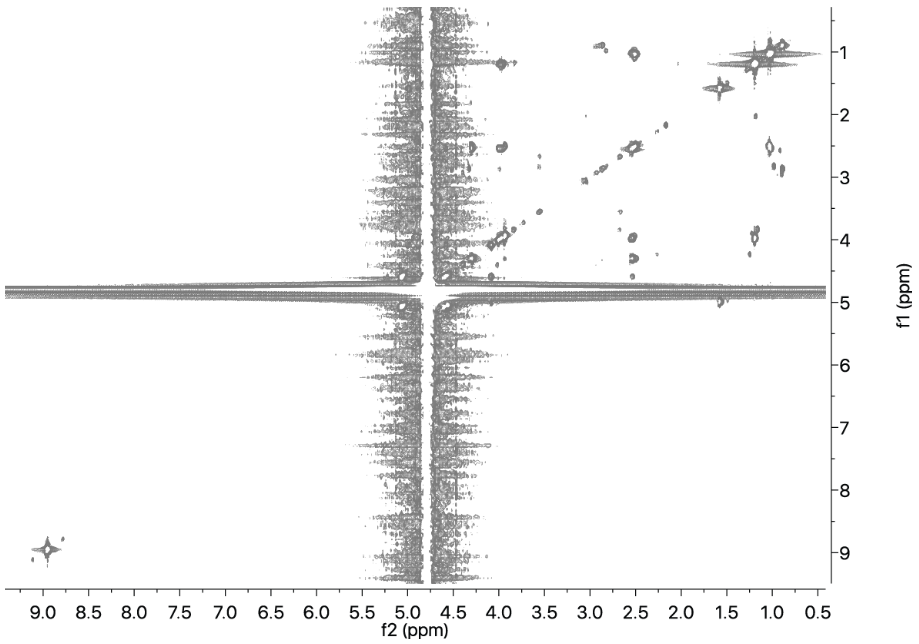

Figure S48. COSY spectrum of the mixture of biapenem and OXA-48. Sample consisted of 2 mM biapenem and 2.5 µM OXA-48 in 50 mM sodium phosphate, pH 7.5, 10 % D_2O, and the spectrum was measured at a field strength of 700 MHz.

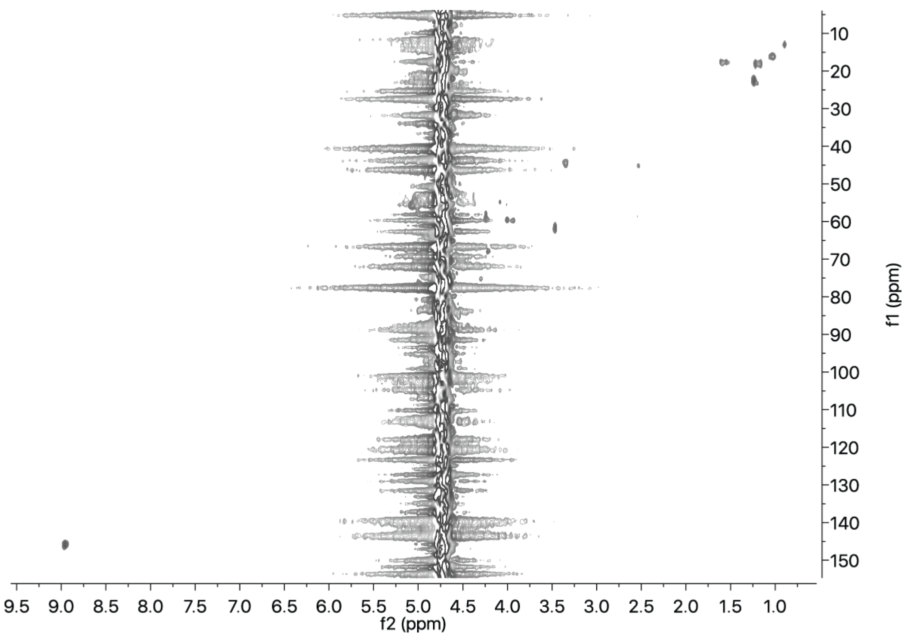

Figure S49. ^{1}H-^{13}C HSQC spectrum of the mixture of biapenem and OXA-48. Sample consisted of 2 mM biapenem and 2.5 μM OXA-48 in 50 mM sodium phosphate, pH 7.5, 10 % D_2O, and the spectrum was measured at a field strength of 700 MHz.

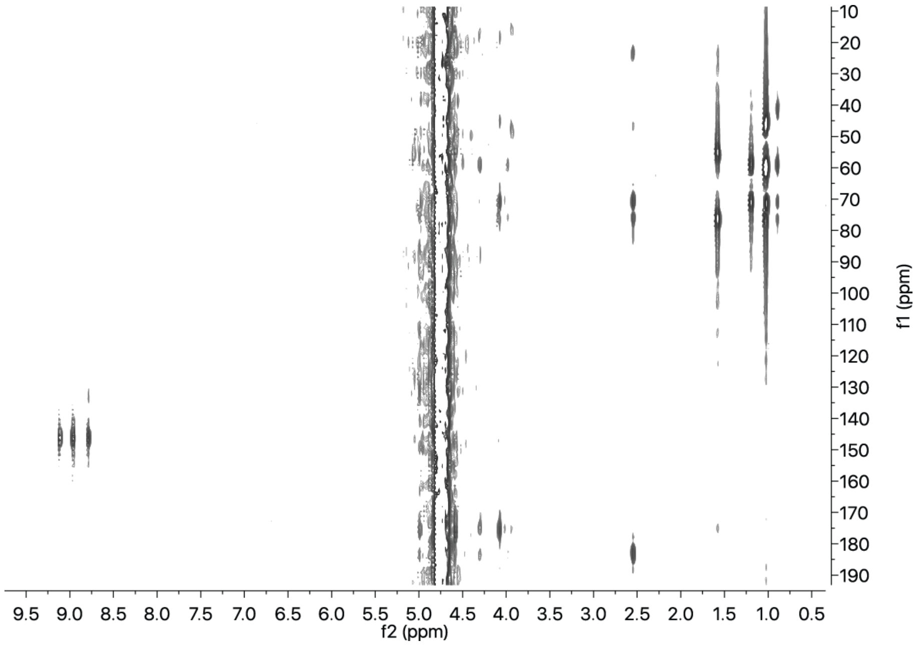

Figure S50. ^{1}H-^{13}C HMBC spectrum of the mixture of biapenem and OXA-48. Sample consisted of 2 mM biapenem and 2.5 μM OXA-48 in 50 mM sodium phosphate, pH 7.5, 10 % D_2O, and the spectrum was measured at a field strength of 700 MHz.

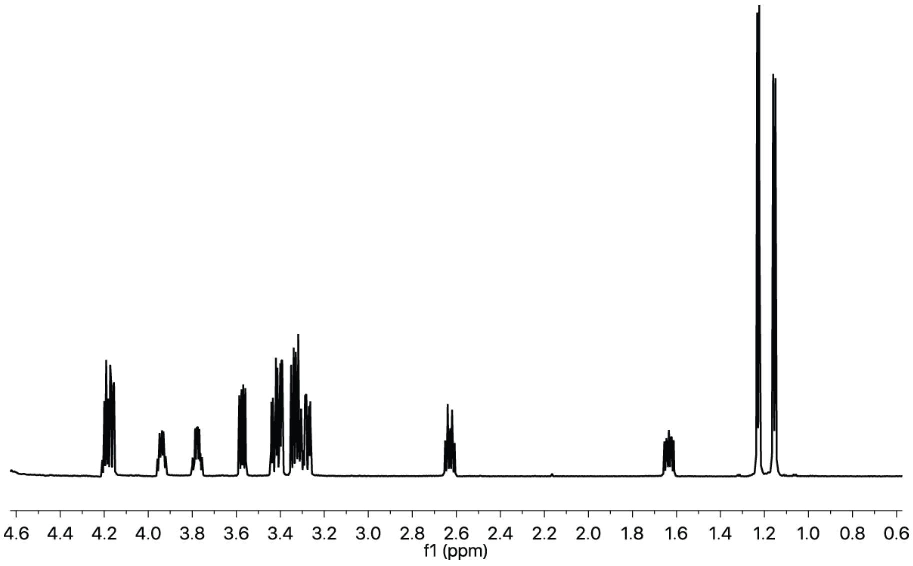

Figure S51. ^{1}H NMR spectrum of doripenem. Sample consisted of 5 mM doripenem in 50 mM sodium phosphate, pH 7.5, 10 % D_2O, and the spectrum was measured at a field strength of 700 MHz.

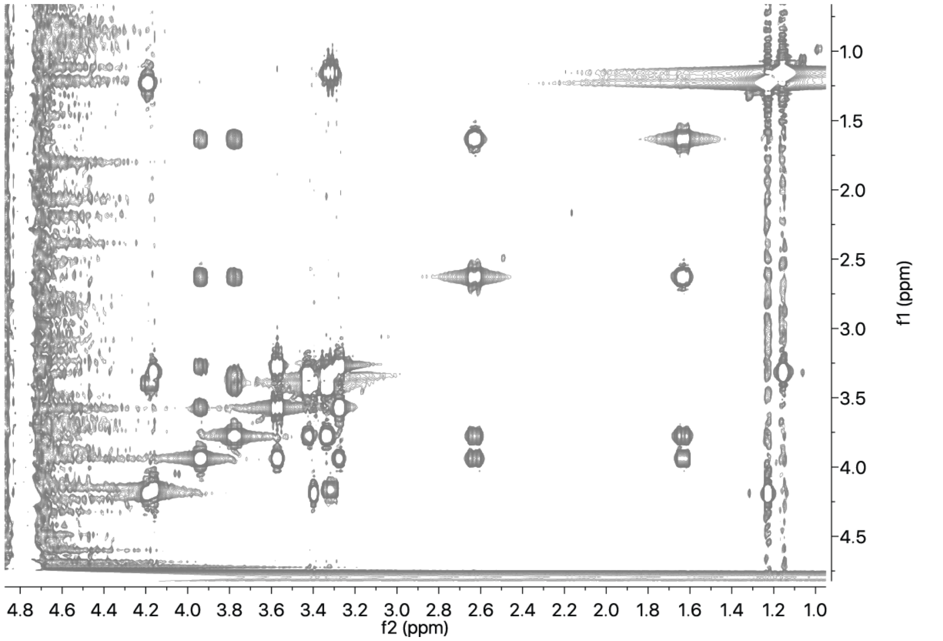

Figure S52. COSY spectrum of doripenem. Sample consisted of 5 mM doripenem in 50 mM sodium phosphate, pH 7.5, 10 % D_2O, and the spectrum was measured at a field strength of 700 MHz.

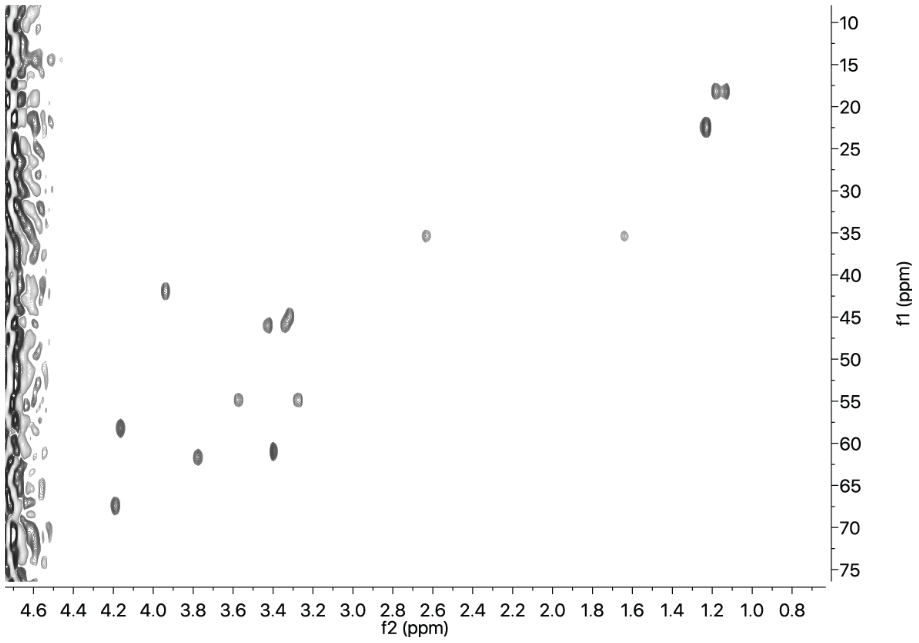

Figure S53. ^{1}H-^{13}C HSQC spectrum of doripenem. Sample consisted of 5 mM doripenem in 50 mM sodium phosphate, pH 7.5, 10 % D_2O, and the spectrum was measured at a field strength of 700 MHz.

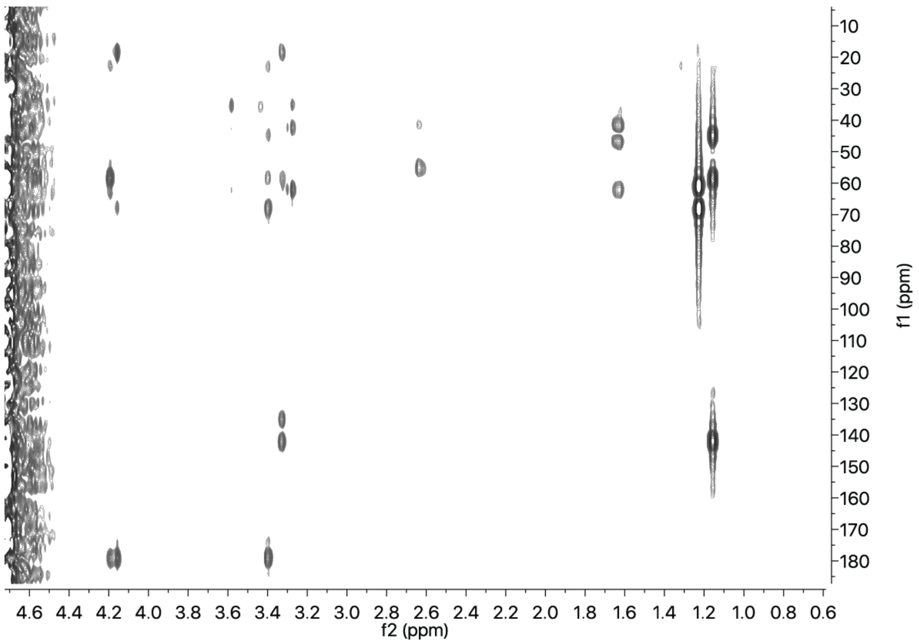

Figure S54. ^{1}H-^{13}C HMBC spectrum of doripenem. Sample consisted of 5 mM doripenem in 50 mM sodium phosphate, pH 7.5, 10 % D_2O, and the spectrum was measured at a field strength of 700 MHz.

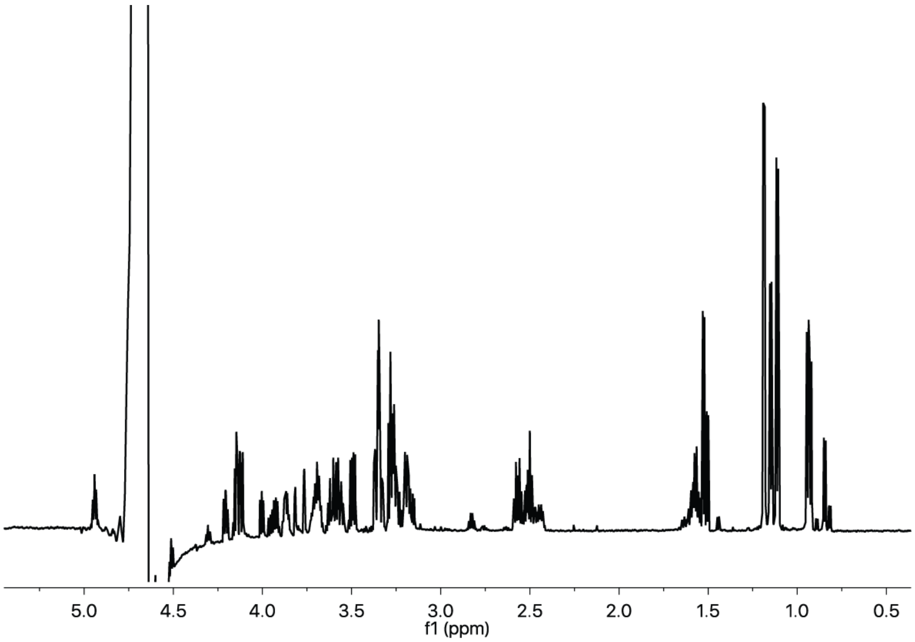

Figure S55. ^{1}H NMR spectrum of the mixture of doripenem and OXA-48. Sample consisted of 2 mM doripenem and 2.8 μM OXA-48 in 50 mM sodium phosphate, pH 7.5, 10 % D_2O, and the spectrum was measured at a field strength of 700 MHz.

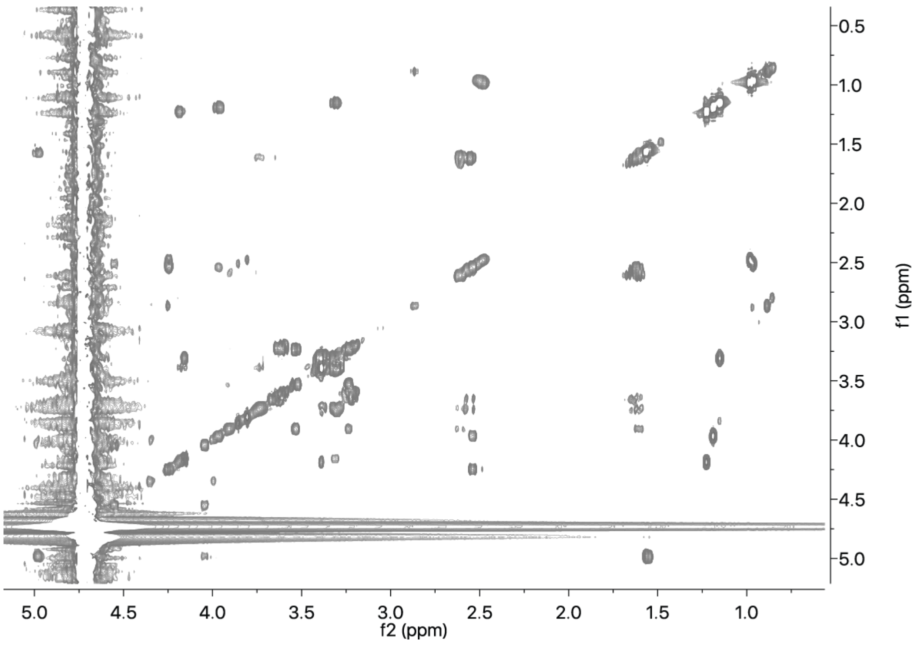

Figure S56. COSY spectrum of the mixture of doripenem and OXA-48. Sample consisted of 2 mM doripenem and 2.8 μM OXA-48 in 50 mM sodium phosphate, pH 7.5, 10 % D_2O, and the spectrum was measured at a field strength of 700 MHz.

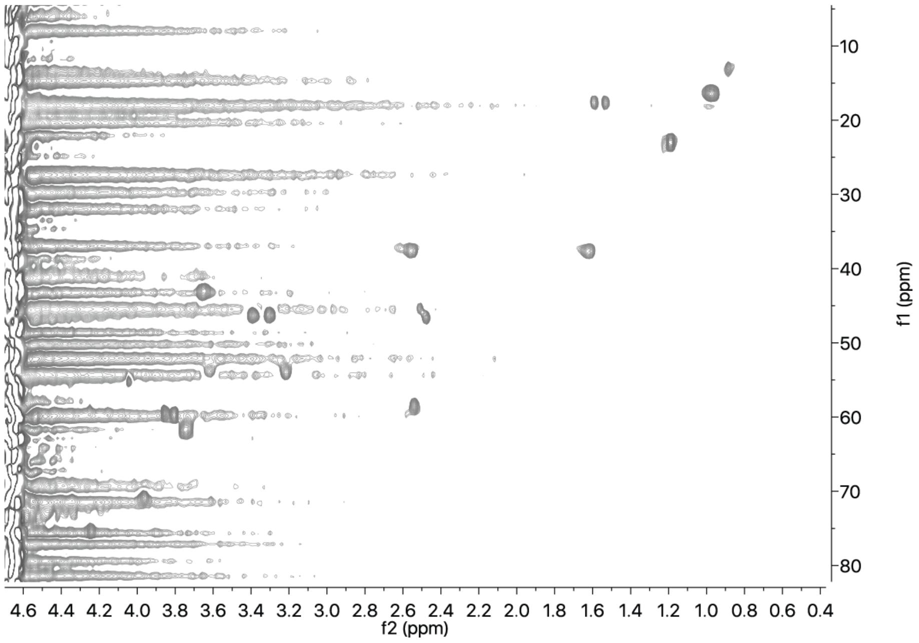

Figure S57. ^{1}H-^{13}C HSQC spectrum of the mixture of doripenem and OXA-48. Sample consisted of 2 mM doripenem and 2.8 μM OXA-48 in 50 mM sodium phosphate, pH 7.5, 10 % D_2O, and the spectrum was measured at a field strength of 700 MHz.

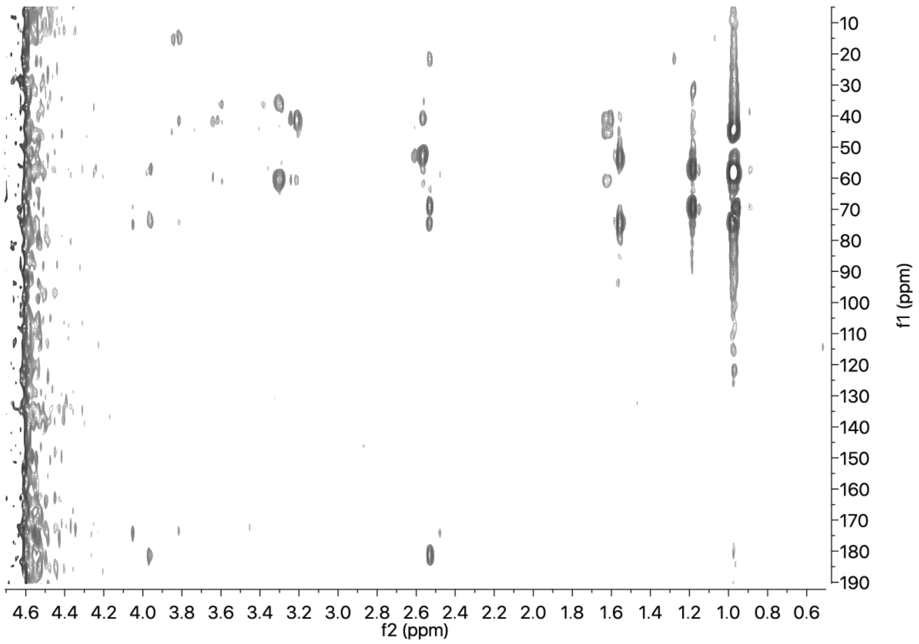

Figure S58. ^{1}H-^{13}C HMBC spectrum of the mixture of doripenem and OXA-48. Sample consisted of 2 mM doripenem and 2.8 μM OXA-48 in 50 mM sodium phosphate, pH 7.5, 10 % D_2O, and the spectrum was measured at a field strength of 700 MHz.

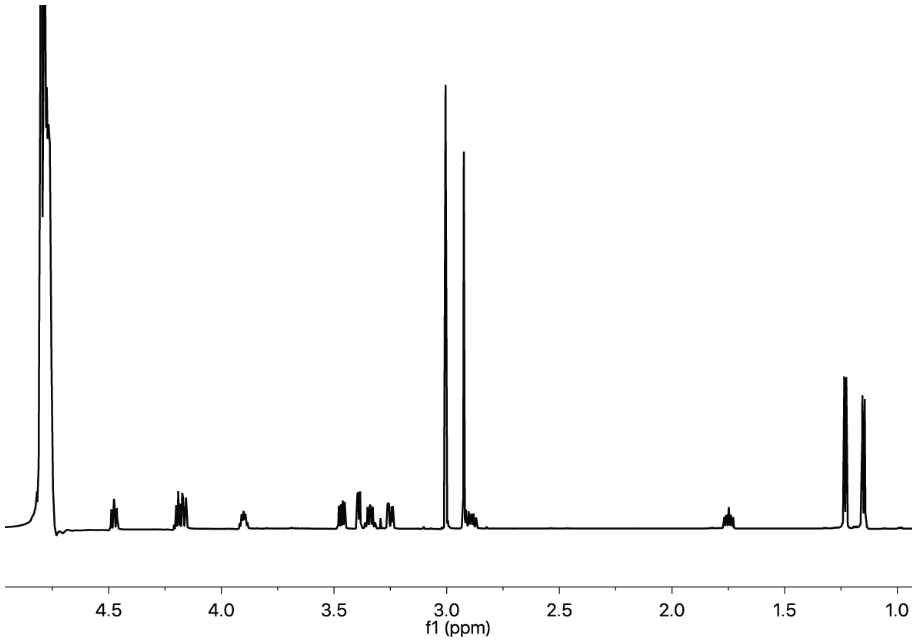

Figure S59. ^{1}H NMR spectrum of meropenem. Sample consisted of 9 mM meropenem in 50 mM sodium phosphate, pH 7.5, 10 % D_2O, and the spectrum was measured at a field strength of 700 MHz.

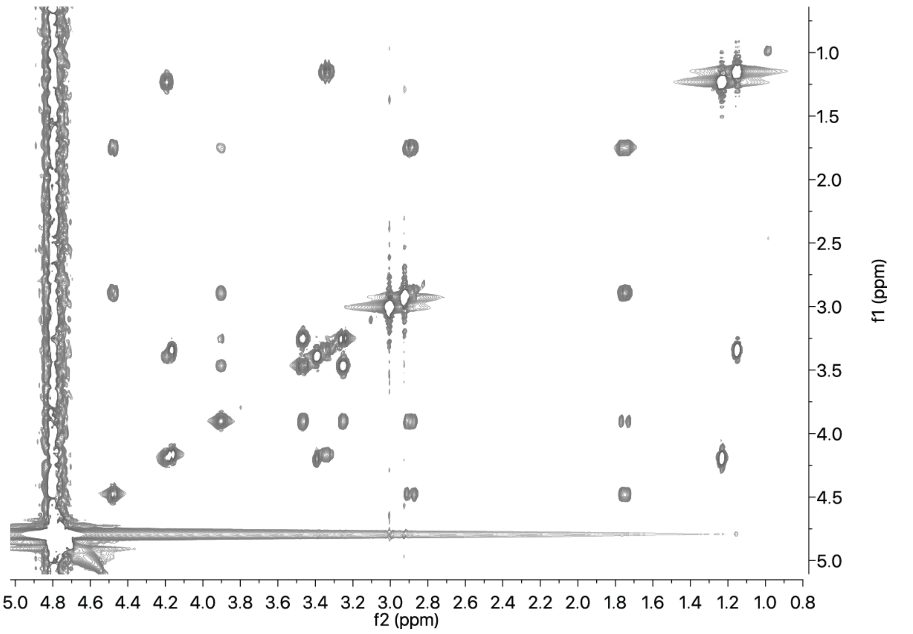

Figure S60. COSY spectrum of meropenem. Sample consisted of 9 mM meropenem in 50 mM sodium phosphate, pH 7.5, 10 % D_2O, and the spectrum was measured at a field strength of 700 MHz.

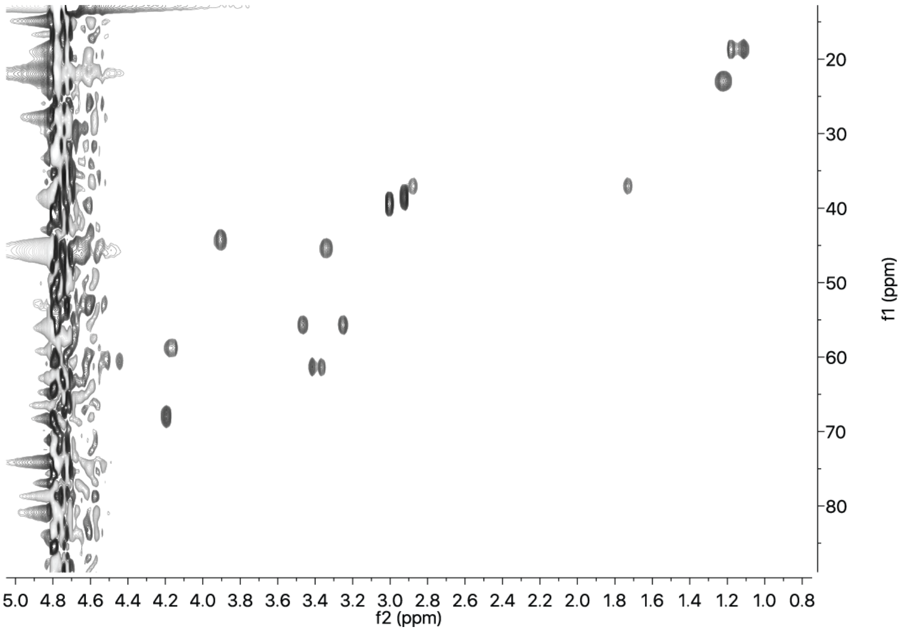

Figure S61. ^1H-^{13}C HSQC spectrum of meropenem. Sample consisted of 9 mM meropenem in 50 mM sodium phosphate, pH 7.5, 10 % D_2O, and the spectrum was measured at a field strength of 700 MHz.

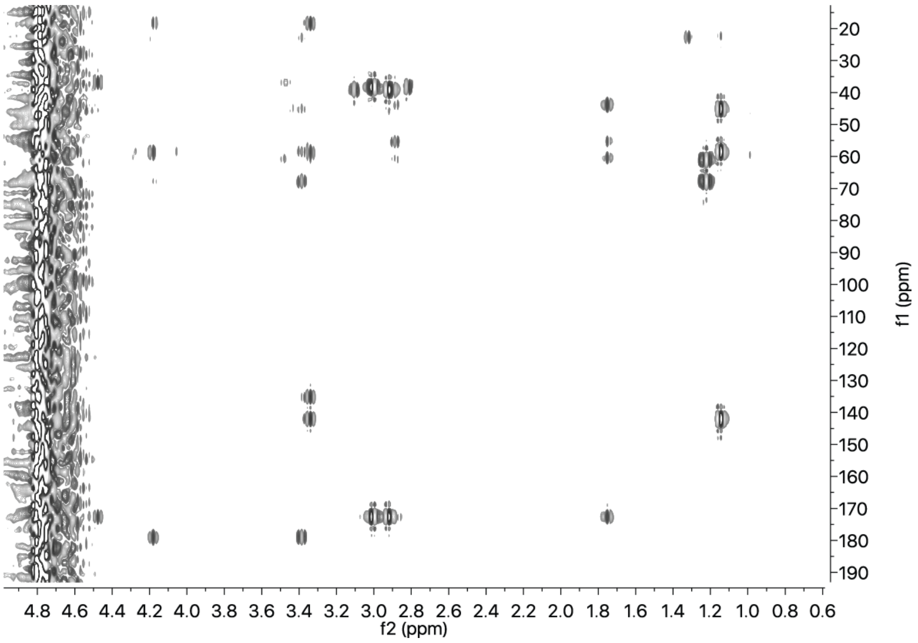

Figure S62. ^1H-^{13}C HMBC spectrum of meropenem. Sample consisted of 9 mM meropenem in 50 mM sodium phosphate, pH 7.5, 10 % D_2O, and the spectrum was measured at a field strength of 700 MHz.

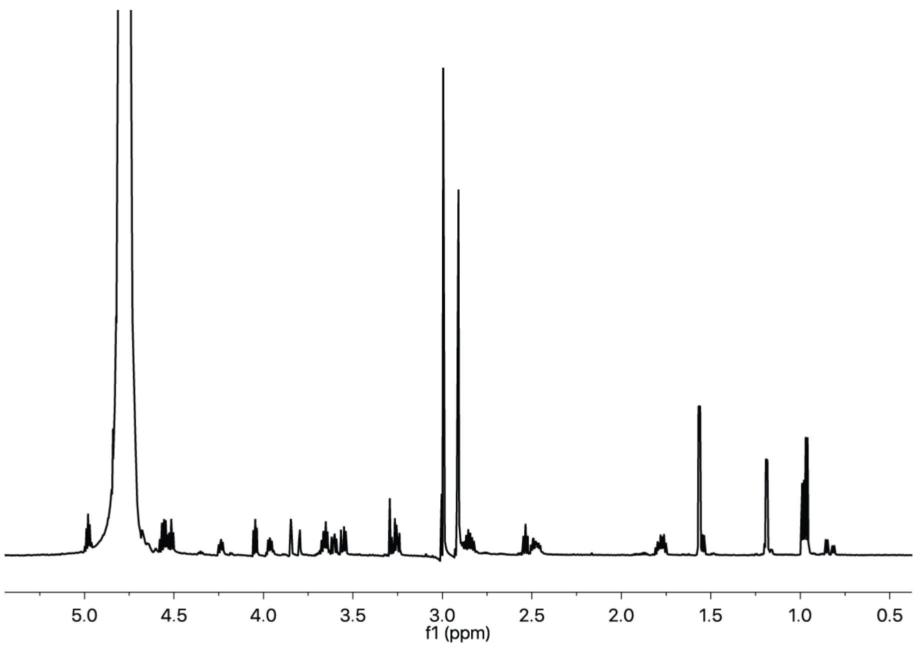

Figure S63. ^{1}H NMR spectrum of the mixture of meropenem and OXA-48. Sample consisted of 2 mM meropenem and 2.5 µM OXA-48 in 50 mM sodium phosphate, pH 7.5, 10 % D_2O, and the spectrum was measured at a field strength of 700 MHz.

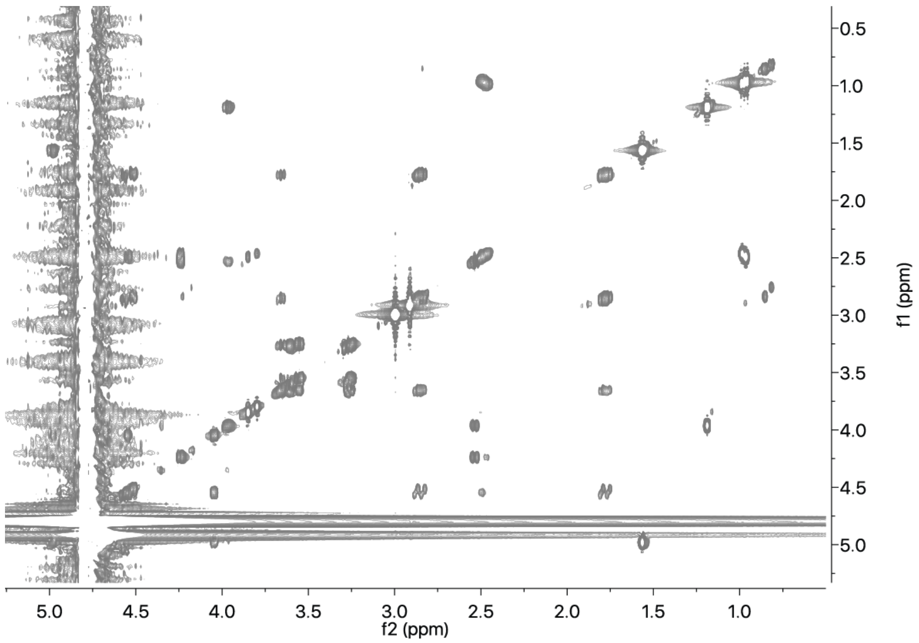

Figure S64. COSY spectrum of the mixture of meropenem and OXA-48. Sample consisted of 2 mM meropenem and 2.5 µM OXA-48 in 50 mM sodium phosphate, pH 7.5, 10 % D_2O, and the spectrum was measured at a field strength of 700 MHz.

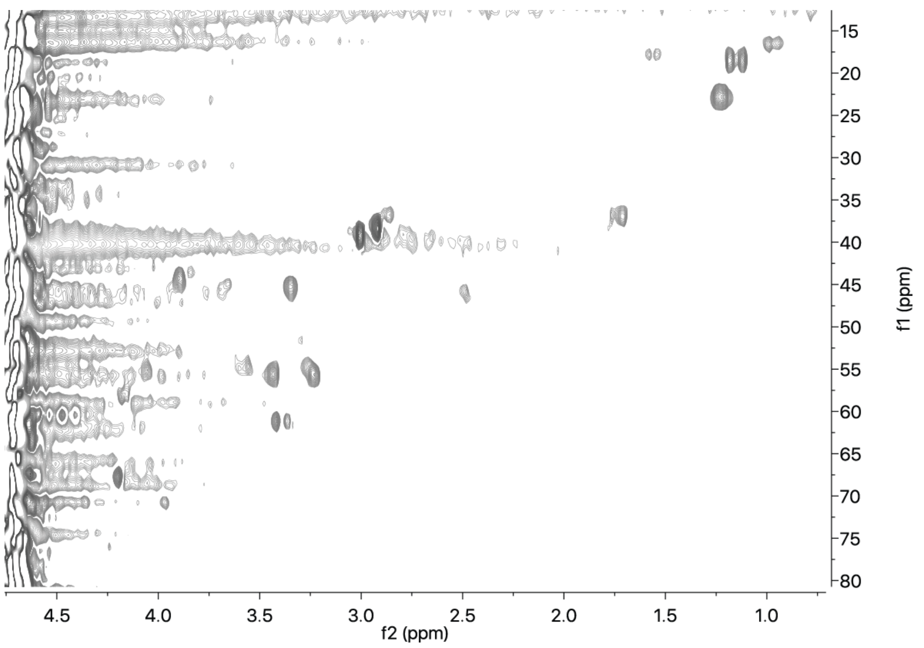

Figure S65. ^{1}H-^{13}C HSQC spectrum of the mixture of meropenem and OXA-48. Sample consisted of 2 mM meropenem and 2.5 µM OXA-48 in 50 mM sodium phosphate, pH 7.5, 10 % D_2O, and the spectrum was measured at a field strength of 700 MHz.

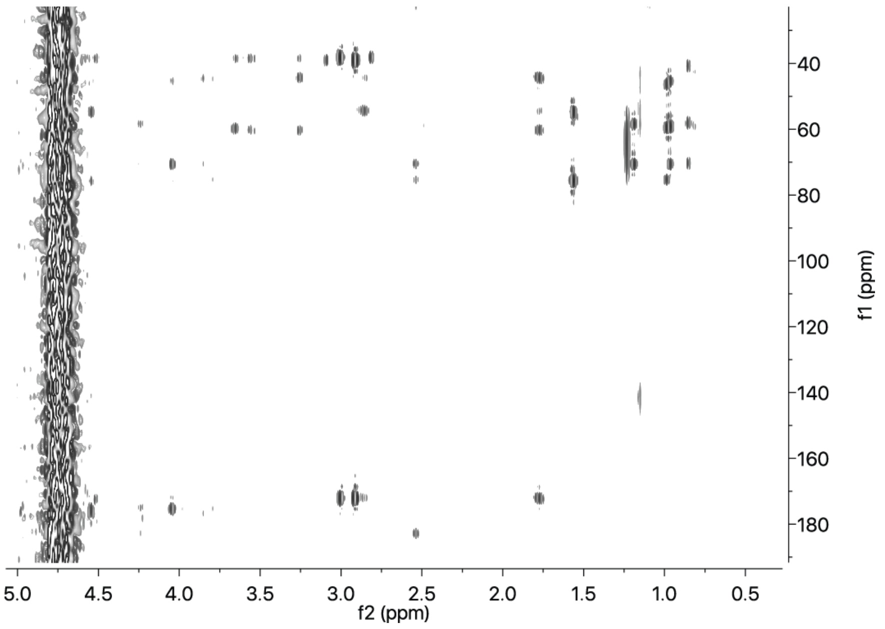

Figure S66. ^{1}H-^{13}C HMBC spectrum of the mixture of meropenem and OXA-48. Sample consisted of 2 mM meropenem and 2.5 µM OXA-48 in 50 mM sodium phosphate, pH 7.5, 10 % D_2O, and the spectrum was measured at a field strength of 700 MHz.

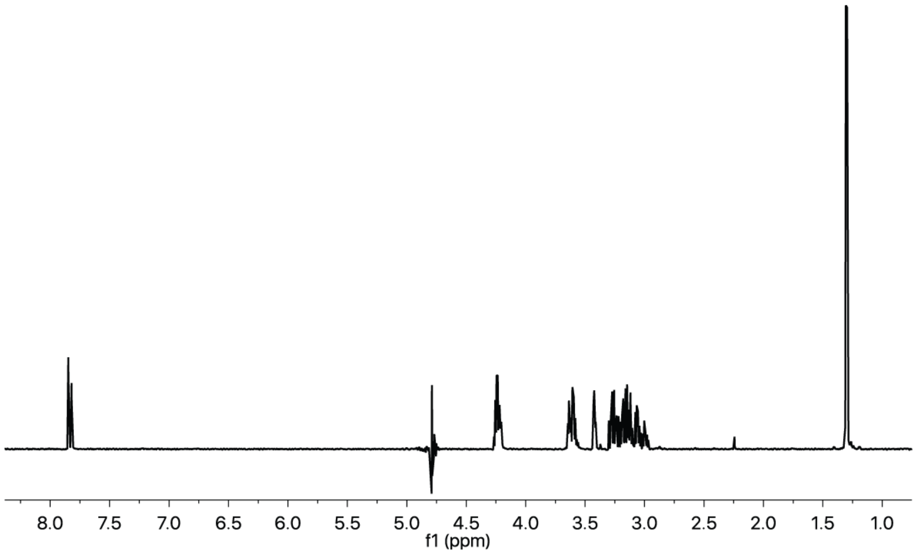

Figure S67. ^{1}H NMR spectrum of imipenem. Sample consisted of 5 mM imipenem in 50 mM sodium phosphate, pH 7.5, 10 % D_2O, and the spectrum was measured at a field strength of 700 MHz.

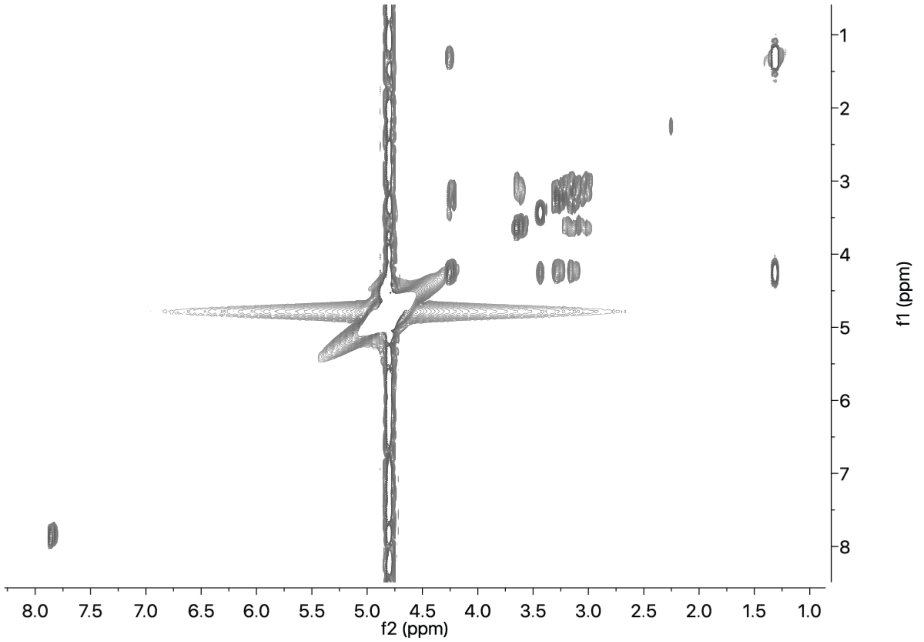

Figure S68. COSY spectrum of imipenem. Sample consisted of 5 mM imipenem in 50 mM sodium phosphate, pH 7.5, 10 % D_2O, and the spectrum was measured at a field strength of 700 MHz.

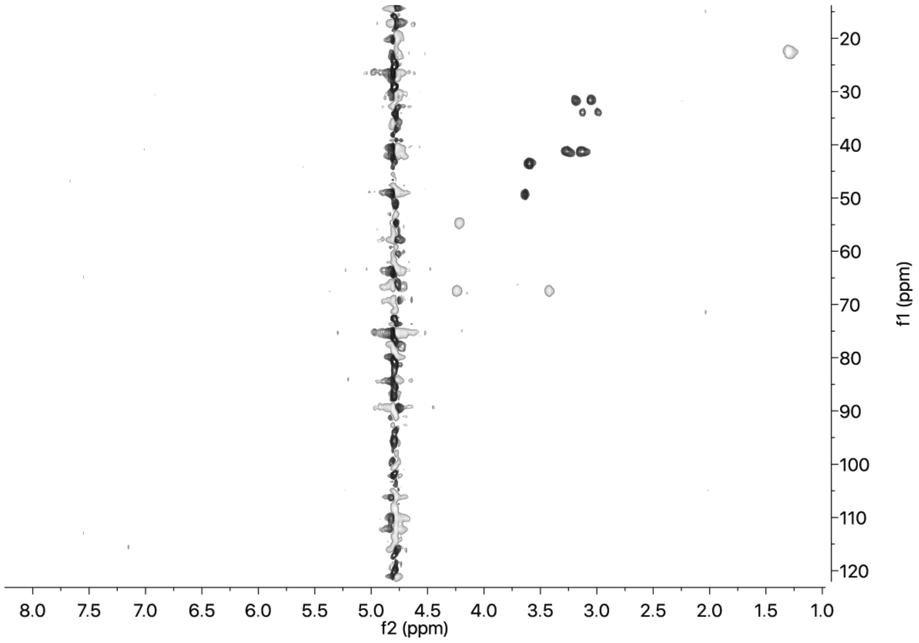

Figure S69. ^{1}H-^{13}C HSQC spectrum of imipenem. Sample consisted of 5 mM imipenem in 50 mM sodium phosphate, pH 7.5, 10 % D_2O, and the spectrum was measured at a field strength of 700 MHz.

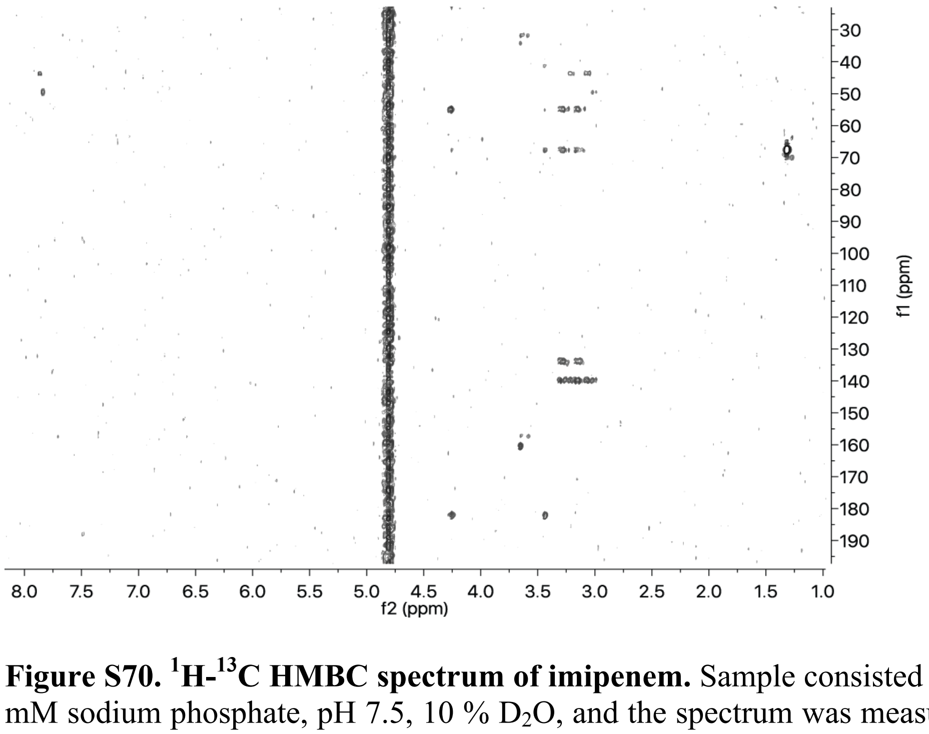

Figure S70. ^{1}H-^{13}C HMBC spectrum of imipenem. Sample consisted of 5 mM imipenem in 50 mM sodium phosphate, pH 7.5, 10 % D_2O, and the spectrum was measured at a field strength of 700 MHz.

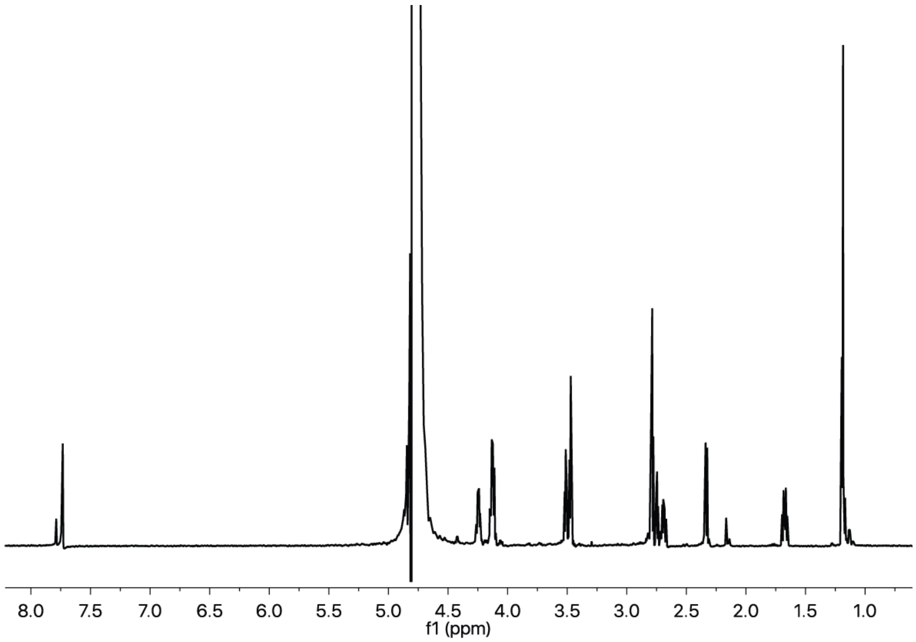

Figure S71. ^{1}H NMR spectrum of the mixture of imipenem and OXA-48. Sample consisted of 2 mM imipenem and 2.5 μM OXA-48 in 50 mM sodium phosphate, pH 7.5, 10 % D_2O, and the spectrum was measured at a field strength of 700 MHz.

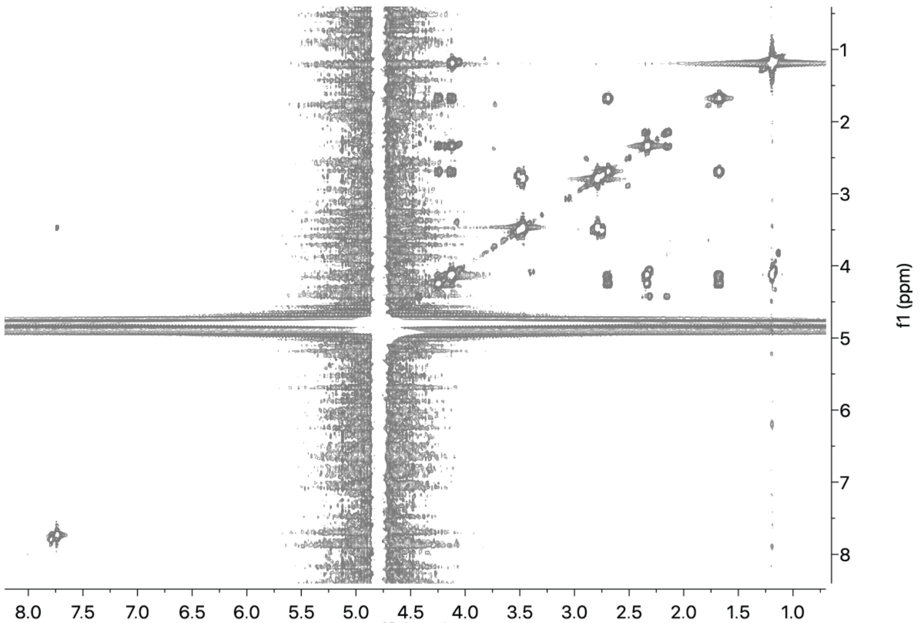

Figure S72. COSY spectrum of the mixture of imipenem and OXA-48. Sample consisted of 2 mM imipenem and 2.5 μM OXA-48 in 50 mM sodium phosphate, pH 7.5, 10 % D_2O, and the spectrum was measured at a field strength of 700 MHz.

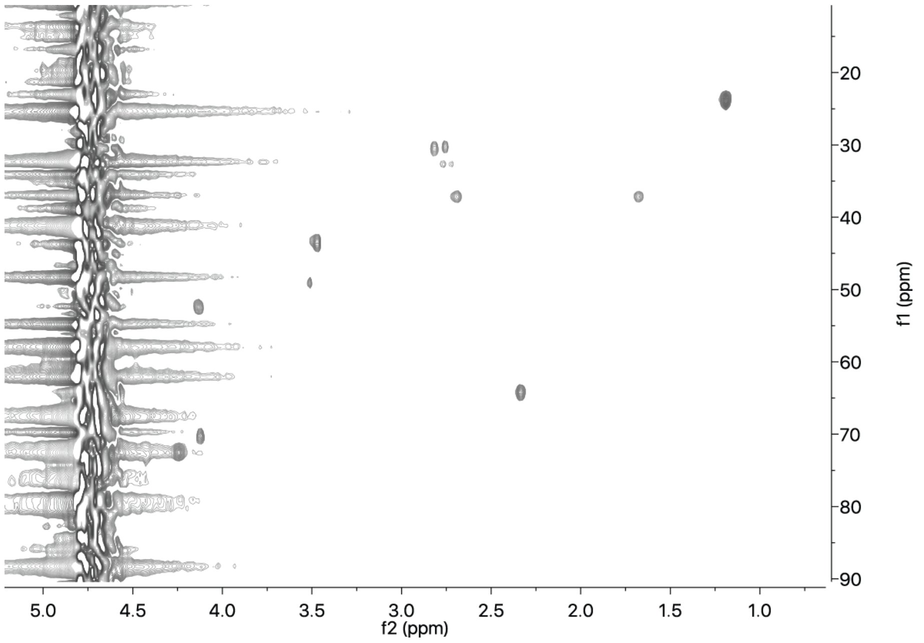

Figure S73. ^{1}H-^{13}C HSQC spectrum of the mixture of imipenem and OXA-48. Sample consisted of 2 mM imipenem and 2.5 µM OXA-48 in 50 mM sodium phosphate, pH 7.5, 10 % D_2O, and the spectrum was measured at a field strength of 700 MHz.

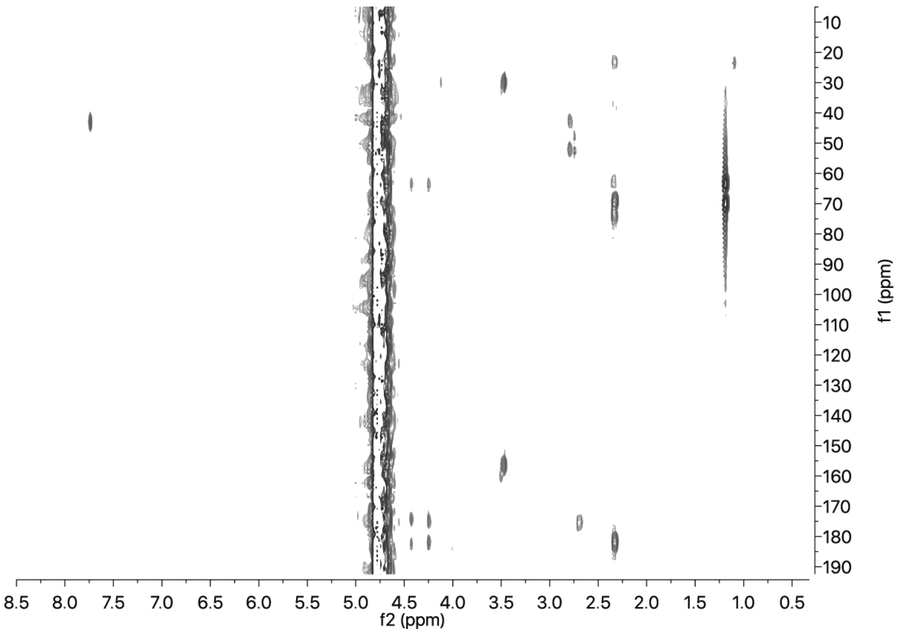

Figure S74. ^{1}H-^{13}C HMBC spectrum of the mixture of imipenem and OXA-48. Sample consisted of 2 mM imipenem and 2.5 µM OXA-48 in 50 mM sodium phosphate, pH 7.5, 10 % D_2O, and the spectrum was measured at a field strength of 700 MHz.

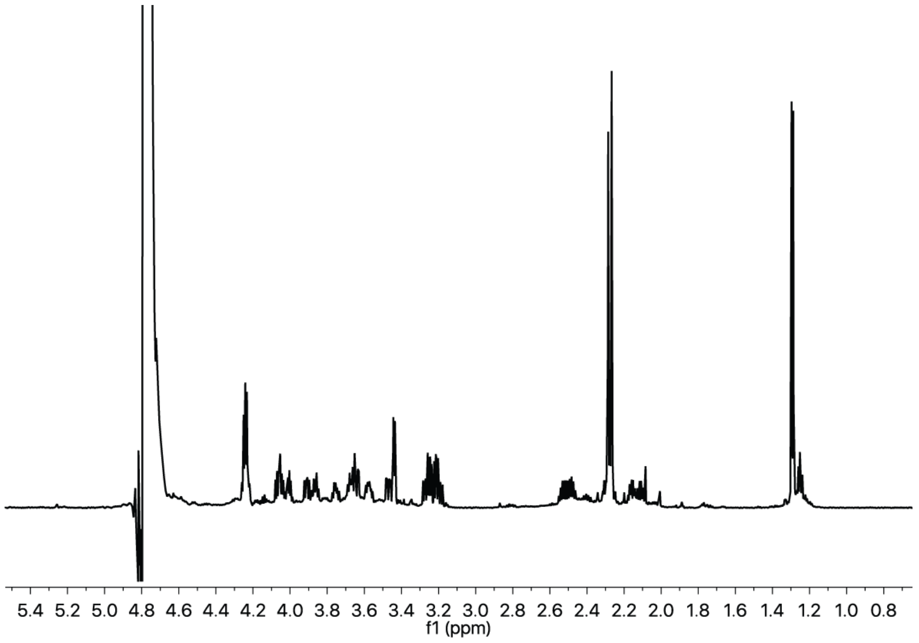

Figure S75. ^{1}H NMR spectrum of panipenem. Sample consisted of 2 mM panipenem in 50 mM sodium phosphate, pH 7.5, 10 % D_2O, and the spectrum was measured at a field strength of 700 MHz.

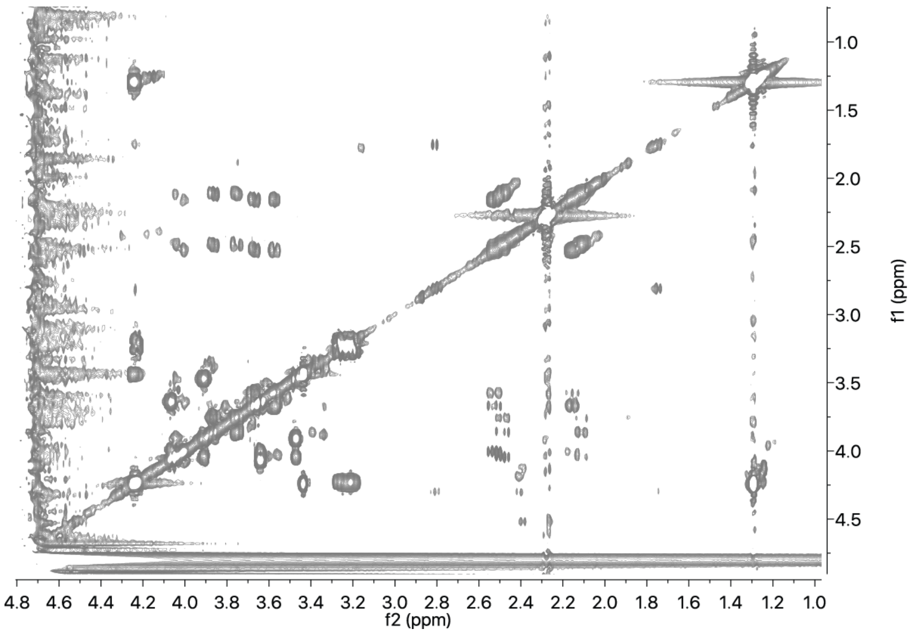

Figure S76. COSY spectrum of panipenem. Sample consisted of 2 mM panipenem in 50 mM sodium phosphate, pH 7.5, 10 % D_2O, and the spectrum was measured at a field strength of 700 MHz.

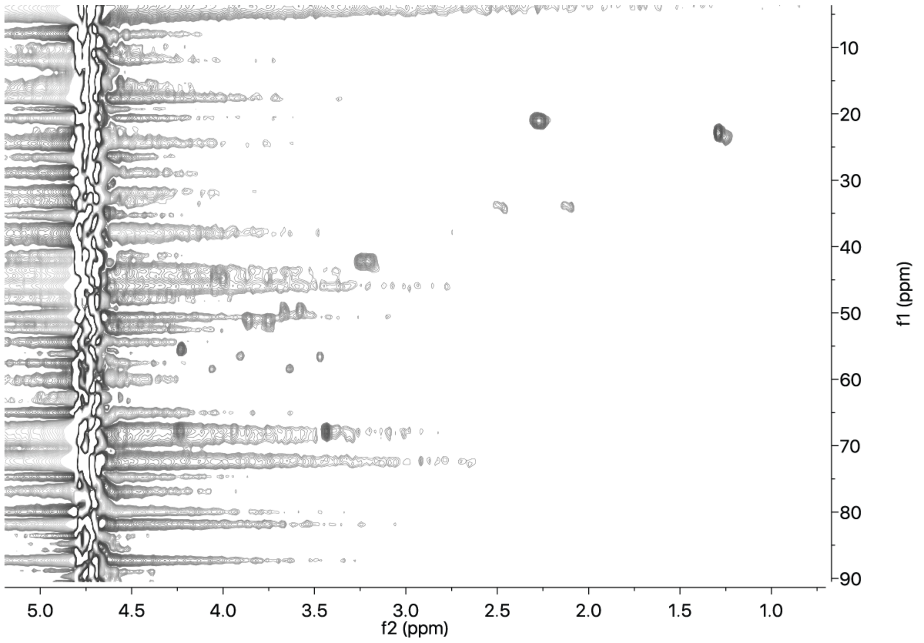

Figure S77. ^{1}H-^{13}C HSQC spectrum of panipenem. Sample consisted of 2 mM panipenem in 50 mM sodium phosphate, pH 7.5, 10 % D_2O, and the spectrum was measured at a field strength of 700 MHz.

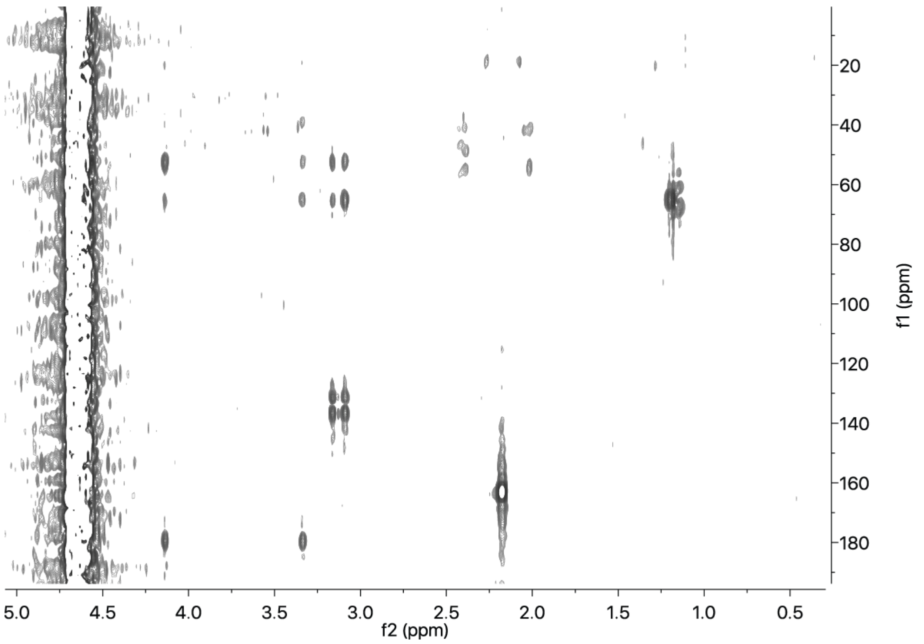

Figure S78. ^{1}H-^{13}C HMBC spectrum of panipenem. Sample consisted of 2 mM panipenem in 50 mM sodium phosphate, pH 7.5, 10 % D_2O, and the spectrum was measured at a field strength of 700 MHz.

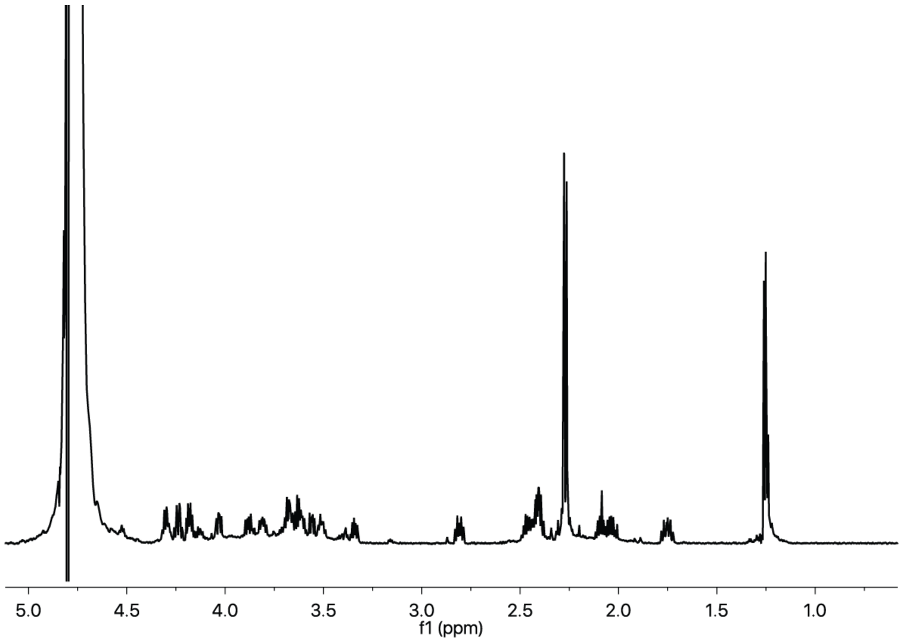

Figure S79. ^{1}H NMR spectrum of the mixture of panipenem and OXA-48. Sample consisted of 2 mM panipenem and 5 µM OXA-48 in 50 mM sodium phosphate, pH 7.5, 10 % D_2O, and the spectrum was measured at a field strength of 700 MHz.

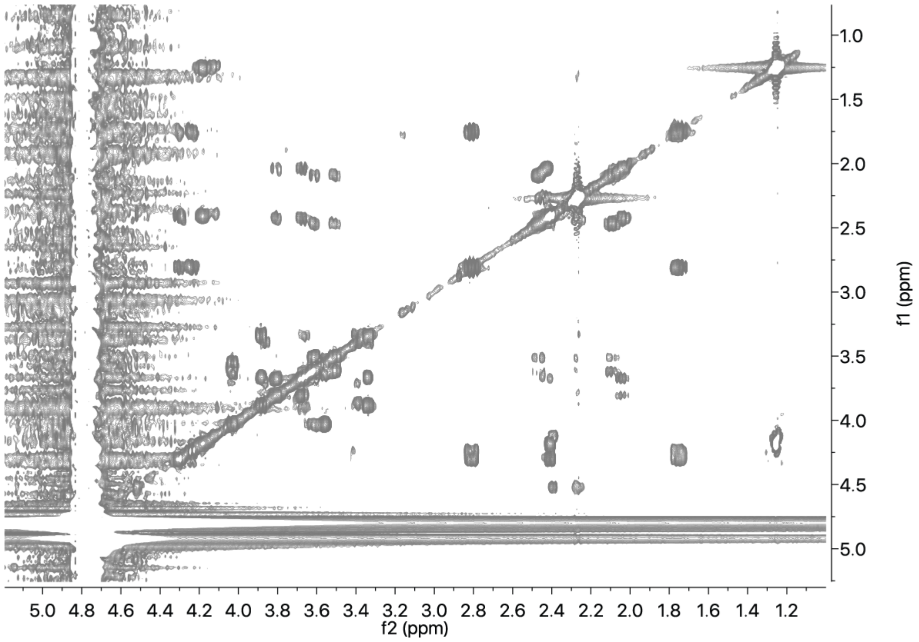

Figure S80. COSY spectrum of the mixture of panipenem and OXA-48. Sample consisted of 2 mM panipenem and 5 µM OXA-48 in 50 mM sodium phosphate, pH 7.5, 10 % D_2O, and the spectrum was measured at a field strength of 700 MHz.

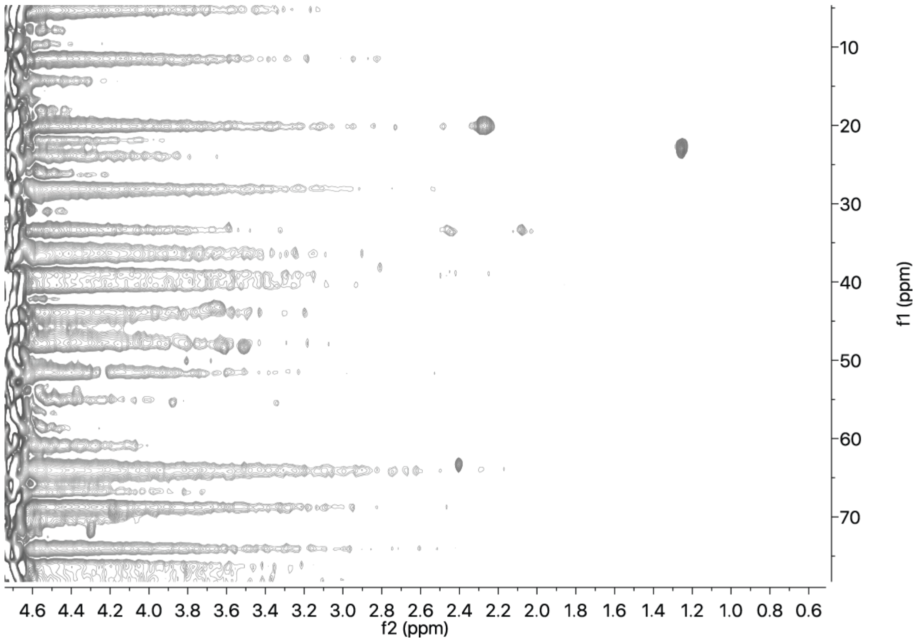

Figure S81. ^{1}H-^{13}C HSQC spectrum of the mixture of panipenem and OXA-48. Sample consisted of 2 mM panipenem and 5 µM OXA-48 in 50 mM sodium phosphate, pH 7.5, 10 % D_2O, and the spectrum was measured at a field strength of 700 MHz. Note that the solubility of panipenem was poor compared to the other carbapenems tested.

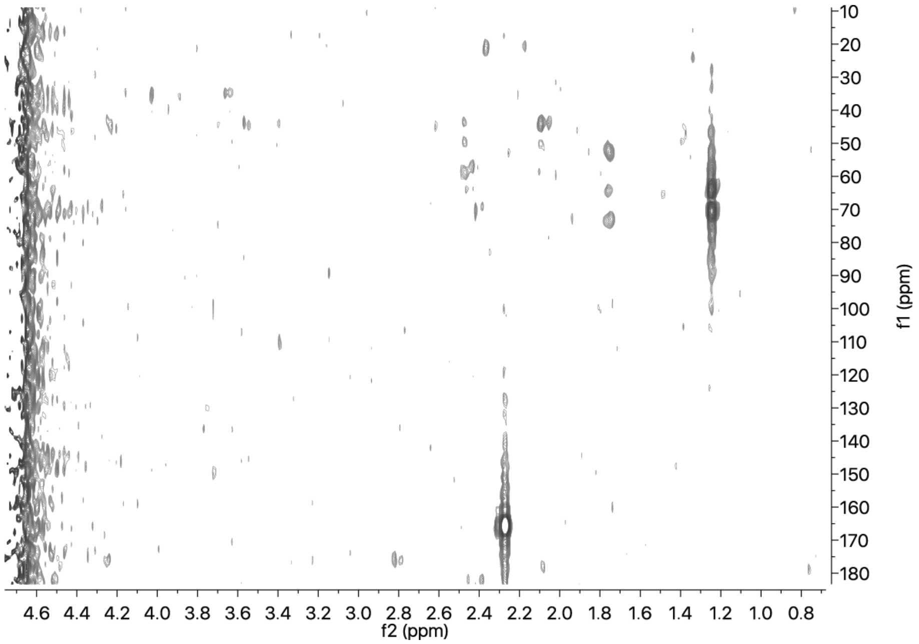

Figure S82. ^{1}H-^{13}C HMBC spectrum of the mixture of panipenem and OXA-48. Sample consisted of 2 mM panipenem and 5 µM OXA-48 in 50 mM sodium phosphate, pH 7.5, 10 % D_2O, and the spectrum was measured at a field strength of 700 MHz. Note that the solubility of panipenem was poor compared to the other carbapenems tested.

References

[1] S. T. Cahill *et al.*, *Antimicrob Agents Chemother* **2017**, *61*, doi: 10.1128/AAC.02260-16

[2] S. Baurin *et al.*, *Biochemistry* **2009**, *48*, 11252.; F. Fonseca, A. C. Sarmento, I. Henriques, B. Samyn, J. van Beeumen, P. Domingues, M. R. Domingues, M. J. Saavedra, A. Correia, *Antimicrob Agents Chemother* **2007**, *51*, 4512.; A. Makena, S. S. van Berkel, C. Lejeune, R. J. Owens, A. Verma, R. Salimraj, J. Spencer, J. Brem, C. J. Schofield, *ChemMedChem* **2013**, *8*, 1923.; K. Calvopiña, K. D. Umland, A. M. Rydzik, P. Hinchliffe, J. Brem, J. Spencer, C. J. Schofield, M. B. Avison, *Antimicrob Agents Chemother* **2016**, *60*, 4170.; C. Bebrone, C. Anne, K. De Vriendt, B. Devreese, G. M. Rossolini, J. Van Beeumen, J. M. Frère, M. Galleni, *J Biol Chem* **2005**, *280*, 28195.; S. R. Inglis, M. Strieker, A. M. Rydzik, A. Dessen, C. J. Schofield, *Anal Biochem* **2012**, *420*, 41.

[3] S. S. van Berkel, J. Brem, A. M. Rydzik, R. Salimraj, R. Cain, A. Verma, R. J. Owens, C. W. Fishwick, J. Spencer, C. J. Schofield, *J Med Chem* **2013**, *56*, 6945.

[4] K. Calvopiña *et al.*, *Mol Microbiol* **2017**, ; J. Brem, R. Cain, S. Cahill, M. A. McDonough, I. J. Clifton, J. C. Jiménez-Castellanos, M. B. Avison, J. Spencer, C. W. Fishwick, C. J. Schofield, *Nat Commun* **2016**, *7*, 12406.

[5] K. D. Schneider, M. E. Karpen, R. A. Bonomo, D. A. Leonard, R. A. Powers, *Biochemistry* **2009**, *48*, 11840.

[6] D. A. Case, T. A. Darden, T. E. Cheatham III, C. L. Simmerling, J. Wang, R. E. Duke, R. Luo, R. C. Walker, W. Zhang, K. M. Merz, *AMBER 12; University of California: San Francisco, 2012*, p. 1.

[7] W. L. Jorgensen, J. Chandrasekhar, J. D. Madura, R. W. Impey, M. L. Klein, *J. Chem. Phys.* **1983**, *79*, 926.

[8] C. I. Bayly, P. Cieplak, W. Cornell, P. A. Kollman, *J. Phys. Chem.* **1993**, *97*, 10269.

[9] H. Wang, F. Dommert, C. Holm, *J. Chem. Phys.* **2010**, *133*, 034117.

[10] J. C. Phillips, R. Braun, W. Wang, J. Gumbart, E. Tajkhorshid, E. Villa, C. Chipot, R. D. Skeel, L. Kale, K. Schulten, *J. Comput. Chem.* **2005**, *26*, 1781.

[11] E. R. Johnson, S. Keinan, P. Mori-Sanchez, J. Contreras-Garcia, A. J. Cohen, W. Yang, *J. Am. Chem. Soc.* **2010**, *132*, 6498.; A. Otero-de-la-Roza, E. R. Johnson, J. Contreras-García, *Phys. Chem. Chem. Phys.* **2012**, *14*, 12165.; G. Saleh, C. Gatti, L. Lo Presti, J. Contreras-García, *Chem. Eur. J.* **2012**, *18*, 15523.

[12] B. D. Morris, R. R. Smyth, S. P. Foster, M. P. Hoffmann, W. L. Roelofs, S. Franke, W. Francke, *J Nat Prod* **2005**, *68*, 26.

[13] E. Saepudin, P. Harrison, *Can J Chem* **1995**, *73*, 1.

[14] A. A. Tymiak, C. A. Culver, M. F. Malley, J. Z. Gougoutas, *J Org Chem* **1985**, *50*, 5491.

[15] W. Eisenreich, E. Kupfer, W. Weber, A. Bacher, *J Biol Chem* **1997**, *272*, 867.

[16] Y. Pu, F. M. Martin, J. C. Vederas, *The Journal of Organic Chemistry* **1991**, *56*, 1280.

[17] N. Ollivier, J. B. Behr, O. El-Mahdi, A. Blanpain, O. Melnyk, *Org Lett* **2005**, *7*, 2647.

[18] R. W. Ratcliffe, K. J. Wildonger, L. Di Michele, A. W. Douglas, R. Hajdu, R. T. Goegelman, J. P. Springer, J. Hirshfield, *J. Org. Chem.* **1989**, *54*, 653.; M. N. Lisa *et al.*, *Nat Commun* **2017**, *8*, 538.

[19] K. D. Schneider, C. J. Ortega, N. A. Renck, R. A. Bonomo, R. A. Powers, D. A. Leonard, *J Mol Biol* **2011**, *406*, 583.

[20] C. A. Smith, N. T. Antunes, N. K. Stewart, M. Toth, M. Kumarasiri, M. Chang, S. Mobashery, S. B. Vakulenko, *Chem Biol* **2013**, *20*, 1107.

[21] S. Pratap, M. Katiki, P. Gill, P. Kumar, D. Golemi-Kotra, *Antimicrob Agents Chemother* **2015**, *60*, 75.
[22] C. M. June, T. J. Muckenthaler, E. C. Schroder, Z. L. Klamer, Z. Wawrzak, R. A. Powers, A. Szarecka, D. A. Leonard, *Protein Sci* **2016**, *25*, 2152.
